# Lysine Possesses the Optimal Chain Length for Histone Lysine Methyltransferase Catalysis

**DOI:** 10.1038/s41598-017-16128-4

**Published:** 2017-11-23

**Authors:** Abbas H. K. Al Temimi, Y. Vijayendar Reddy, Paul B. White, Hong Guo, Ping Qian, Jasmin Mecinović

**Affiliations:** 1 Institute for Molecules and Materials, Radboud University, Nijmegen, The Netherlands; 2 Department of Biochemistry and Cellular and Molecular Biology, University of Tennessee, Knoxville, USA; 3 UT/ORNL Center for Molecular Biophysics, Oak Ridge National Laboratory, Oak Ridge, USA; 4 Chemistry and Material Science Faculty, Shandong Agricultural University, Taian, P.R. China

## Abstract

Histone lysine methyltransferases (KMTs) represent an important class of epigenetic enzymes that play essential roles in regulation of gene expression in humans. Members of the KMT family catalyze the transfer of the methyl group from *S*-adenosylmethionine (SAM) to lysine residues in histone tails and core histones. Here we report combined MALDI-TOF MS experiments, NMR analyses and quantum mechanical/molecular dynamics studies on human KMT-catalyzed methylation of the most related shorter and longer lysine analogues, namely ornithine and homolysine, in model histone peptides. Our experimental work demonstrates that while lysine is an excellent natural substrate for KMTs, ornithine and homolysine are not. This study reveals that ornithine does not undergo KMT-catalyzed methylation reactions, whereas homolysine can be methylated by representative examples of human KMTs. The results demonstrate that the specificity of KMTs is highly sensitive to the side chain length of the residue to be methylated. The origin for the degree of the observed activities of KMTs on ornithine and homolysine is discussed.

## Introduction

The epigenetics regulation of gene expression is mediated by DNA modification or by numerous posttranslational modifications of the associated histone proteins^1,2^. Among these, histone lysine methylation has been shown to be directly linked with the active or suppressed regions of the human genome, depending on the type of histone, the lysine site and the lysine methylation state^3–5^. In humans, histone lysine methylation is catalyzed by a large family of histone lysine methyltransferases (KMTs), using *S*-adenosylmethionine (SAM) as a cosubstrate^6^. All human KMTs, along with members of the related histone arginine methyltransferases, contain the 130 amino acid-sequence of SET domain^7–13^. DOT1L, the H3K79 methyltransferase, is the only example of KMTs that does not contain the canonical SET domain.

**Figure 1:**
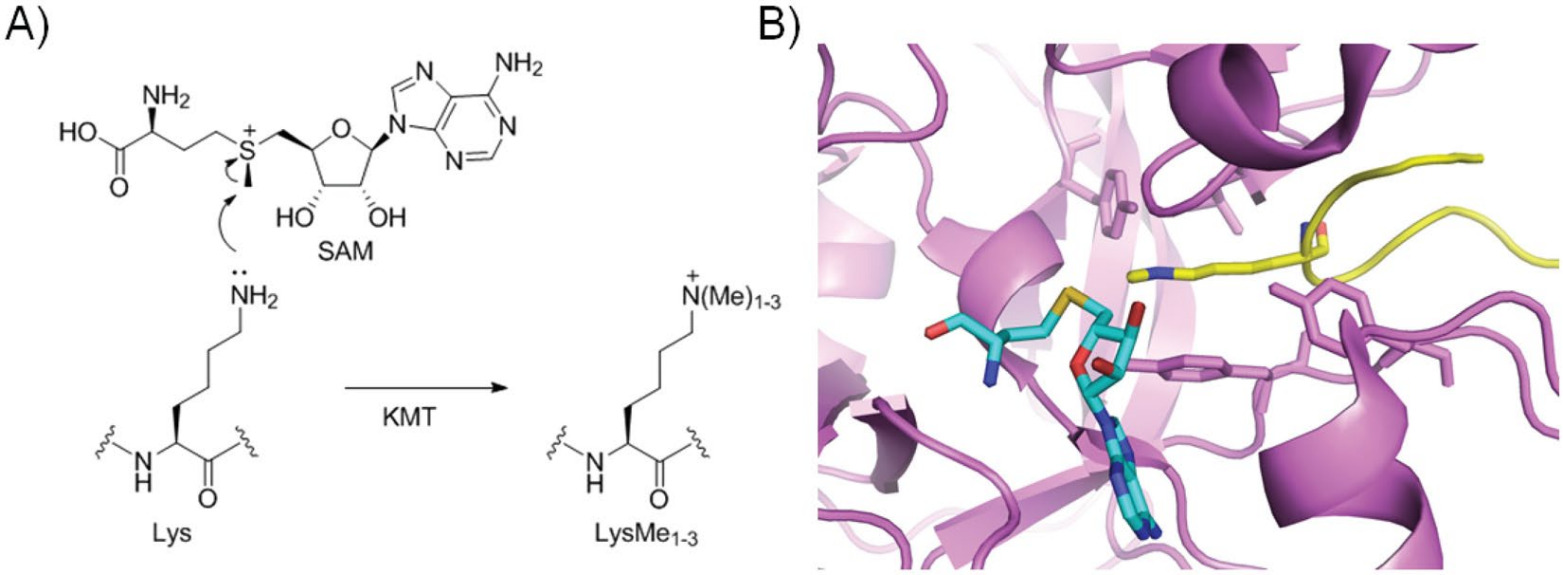
Histone lysine methyltransferase (KMT) catalysis. (**A**) Mechanism of KMT-catalyzed methylation of lysine in the presence of S-adenosylmethionine (SAM); (**B**) View on the crystal structure of SETD7 methyltransferase (magenta) in complex with H3K4me (yellow) and S-adenosylhomocysteine (SAH, cyan) (PDB: 1O9S).

Recent mechanistic and structural studies on KMTs have provided the detailed information for the transfer of the methyl group from SAM to the nucleophilic N^ε^ of lysine *via* the S_N_2 reaction, leading to a methylated lysine product and *S*-adenosylhomocysteine (SAH) (Fig. 1)^7–13^. The histone substrate and the SAM cosubstrate bind to two distinct sites of KMTs that join at the catalytic site of the SET domain (Fig. 1)^7–13^. The lysine residue that undergoes the methylation reaction is anchored in a narrow apolar channel. Previous structural, mutagenesis and enzyme kinetics studies, along with quantum mechanical/molecular mechanical simulations, highlighted that the composition of the active site of KMT directly affects the final methylation state of the methylated lysine products^7,8,10–15^. Several studies have clearly demonstrated that the presence of the conserved active site tyrosine (e.g. Y335 in SETD7, Y1154 in G9a) plays the vital role in the enzymatic activity^11^. The exact role for this tyrosine is still not clear, although it was proposed that it might act as a general base for deprotonation of the positively charged ammonium (and methylammonium) group for the methylation reaction to occur. The presence of additional tyrosine and phenylalanine residues in the active site of KMTs seems to control whether the enzymes catalyze mono-, di- or trimethylation of lysine. These tyrosine residues (e.g. Y245 in SETD7, Y1067 in G9a) form H-bonds with the target lysine in the X-ray structures, and thus may contribute to the proper alignment of the lone pair of the N^ε^ with the electrophilic transferable methyl group of SAM.

**Figure 2:**
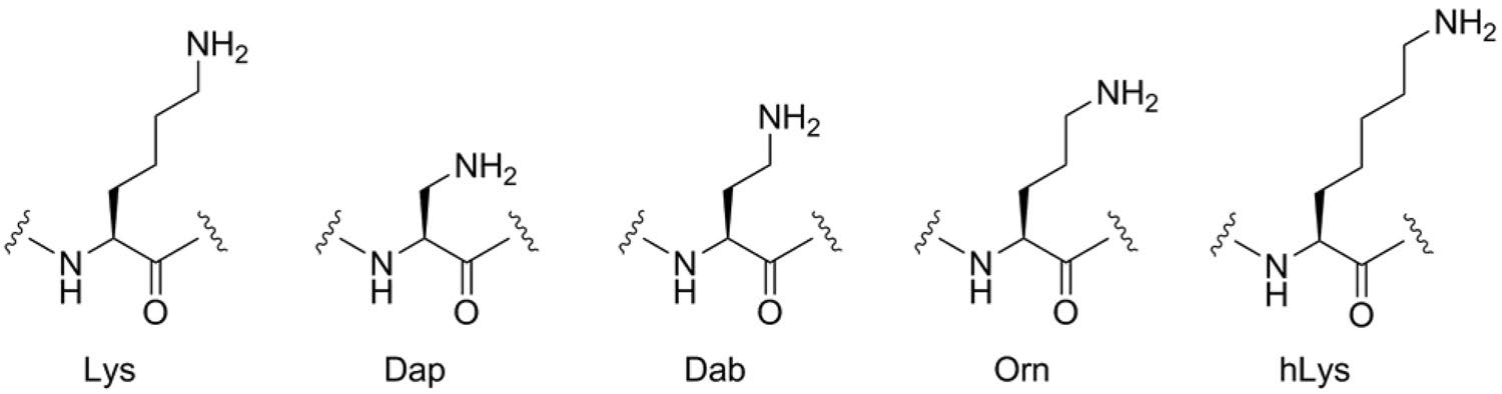
Lysine and its analogues that possess different lengths of the side chain.

Although a large body of biochemical data has provided clear evidence that lysine is the target residue in natural substrates for numerous KMTs, a key question that has not been investigated is whether this biologically important family of enzymes could also have a potential to methylate some simplest lysine analogues with different lengths of the side chain. Herein we report experimental and computational analyses of KMT-catalyzed methylation of natural lysine substrate, along with shorter ornithine and longer homolysine analogues (Fig. 2). Our comparative work highlights that lysine exhibits the perfect length of the side chain for SAM-mediated methyl transfer reactions.

## Results

### Selection of histone lysine methyltransferases and histone peptides

We selected four human histone lysine methyltransferases for the study that examines the effect of changing the side chain length (i.e., from lysine to its analogues) on the efficiency of the KMT catalysis. The KMTs that were selected include: (i) SETD7 that catalyzes monomethylation of H3K4^16^; (ii) G9a that catalyzes mono-, di- and trimethylation of H3K9^17^; (iii) GLP that catalyzes mono-, di- and trimethylation of H3K9^17,18^; and (iv) SETD8 that catalyzes monomethylation of H4K20^19^. These members of KMT family have been structurally and functionally characterized, and all have been recognized as validated targets for epigenetic drug discovery^20,21^. Therefore, we envisioned that the studies on these representative members of KMT family would advance our fundamental understanding of their enzymatic function, and consequently shed light on their inhibition.

Histone peptides that contain lysine, ornithine and homolysine were synthesized using the established solid-phase peptide synthesis protocol. The following histone peptides were synthesized: i) H3K4, H3Orn4 and H3hK4 (residues 1–15) for SETD7 catalysis; ii) H3K9, H3Orn9 and H3hK9 (residues 1–15) and H3K9 (residues 1–14) for G9a/GLP catalysis; and iii) H4K20, H4Orn20 and H4hK20 (residues 13–27) for SETD8 catalysis. We also incorporated shorter Dap and Dab at position 4 of H3 for the evaluation by SETD7. In addition, we synthesized p53 peptides (residues 365–379) that possess K373/Orn373/hK373 for the evaluation by G9a and GLP, and K372/Orn372/hK372 for the evaluation by SETD7. All histone and p53 peptides were obtained in pure form by preparative HPLC (Supplementary Figures S1–S16).

### MALDI-TOF MS assays for KMT-catalyzed methylation

**Figure 3:**
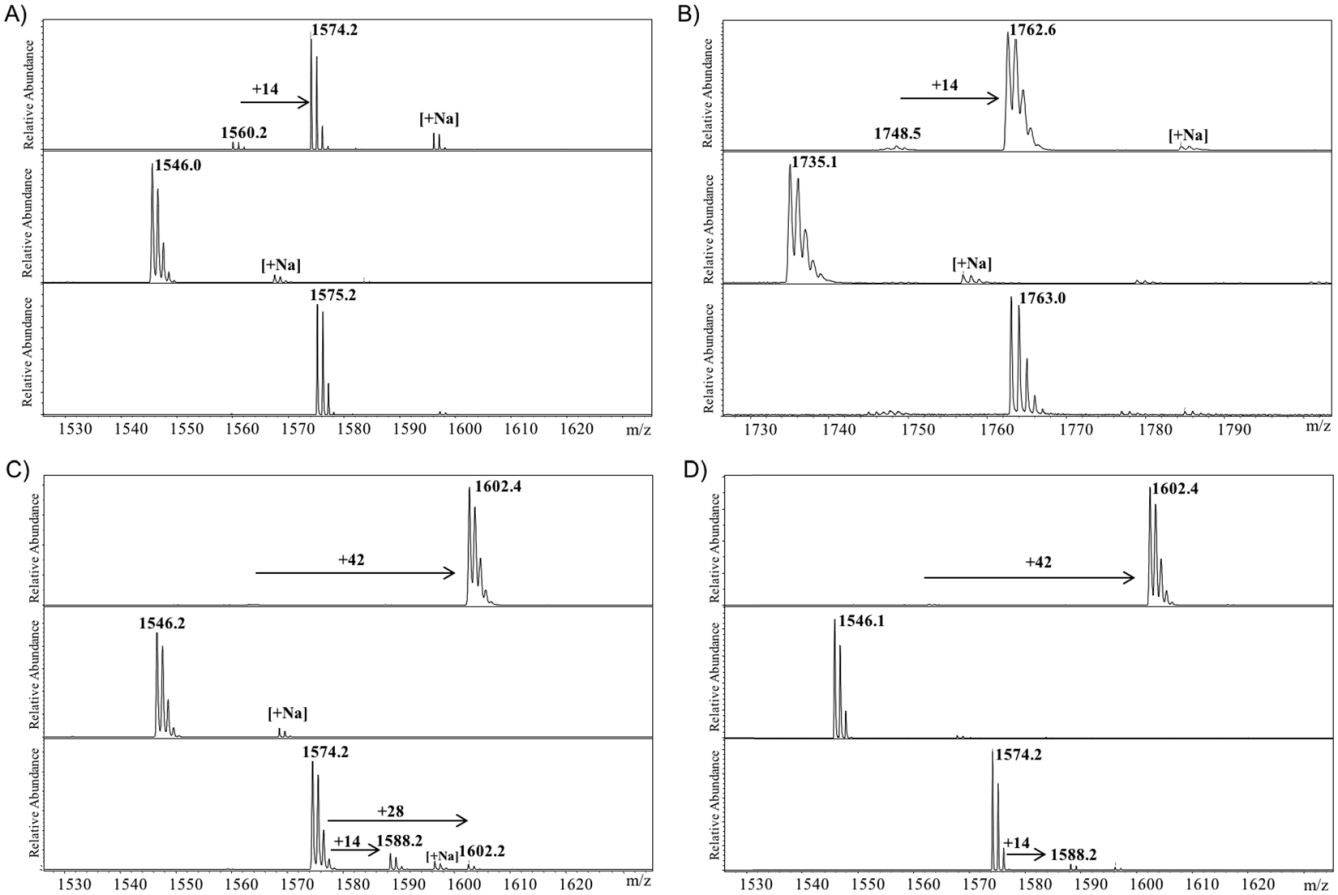
Histone lysine methyltransferase (KMT)-catalyzed methylation of histone peptides in the presence of SAM cosubstrate as monitored by MALDI-TOF MS. (**A**) SETD7 with H3K4 (top), H3Orn4 (middle), H3hLys4 (bottom); (**B**) SETD8 with H4K20 (top), H4Orn20 (middle), H4hLys20 (bottom); (**C**) G9a with H3K9 (top), H3Orn9 (middle), H3hLys9 (bottom); (**D**) GLP with H3K9 (top), H3Orn9 (middle), H3hLys9 (bottom).

We examined histone peptides that possess natural lysine and unnatural lysine analogues as substrates for histone lysine methyltransferases employing MALDI-TOF MS, a well-established technique that enables studies of various posttranslational modifications of proteins/peptides, including those involved in epigenetic processes. First, we verified that all recombinantly expressed wild-type enzymes efficiently catalyze methylation of lysine (Fig. 3, top panels). Human SETD7 was observed to catalyze almost complete (>90%) SAM-dependent monomethylation of H3K4 to afford H3K4me (Fig. 3A, top panel). Similarly, H4K20 (100 μM) underwent virtually quantitative monomethylation in the presence of human SETD8 (2 μM) and SAM (200 μM) in Tris-buffer (50 mM, pH 8.0) after 1 hour at 37 °C (Fig. 3B, top panel).

Recent reports showed some conflicting data that G9a and GLP could catalyze mono- and dimethylation of H3K9 or trimethylation of H3K9^17^. Our MALDI-TOF MS analyses revealed that both G9a (2 μM) and GLP (2 μM) catalyze quantitative trimethylation of H3K9 (100 μM) in the presence of SAM (500 μM) after 1 hour incubation at 37 °C, as exemplified by the mass shift of +42 Da (Fig. 3C and D, top panels). Control experiments in the absence of KMTs showed that no methylation of histone peptides was observed after 1 hour at 37 °C, confirming that methylation reactions are KMT-dependent (Supplementary Figures S17–S20).

We initially screened the entire panel of lysine analogues that bear shorter and longer side chains, i.e. Dap, Dab, Orn, hLys, as potential substrates for SETD7 (Figs 2 and Supplementary Figure S21). None of these lysine analogues were observed to be methylated in the presence of SETD7 (2 μM) and SAM (200 μM) under standard reaction conditions (1 hour at 37 °C) that efficiently yielded H3K4me (see above); only starting histone peptides were observed in MALDI-TOF spectra. We were particularly surprised that no monomethylated products were detected with Orn and hLys, because their side chain lengths are the most similar (though still significantly different) to the one of the natural lysine (Fig. 3A, middle and bottom panels). In line with Orn and hLys, shortening the Lys side chain by two methylenes (Dab) or three methylenes (Dap) results in no methylation too (Supplementary Figure S21). Increased amounts of SETD7 (10 μM), SAM (1 mM) and longer incubation time (6 hours) also did not yield any methylated ornithine and homolysine (Supplementary Figures S22–S23), implying that SETD7 is extremely sensitive to the length of the side chain that undergoes productive enzymatic methylation. Similar to the results with SETD7, we did not observe any SETD8-catalyzed methylation of H4Orn20 and H4hK20 under standard conditions (Fig. 3B, middle and bottom panels). Moreover, no methylation reactions were detected with Orn and hLys when additional SETD8 (10 μM) and SAM (1 mM) were used (Supplementary Figures S24–S25).

Next, we examined H3Orn9 and H3hK9 as potential substrates for G9a and GLP under standard conditions (see above) that led to the formation of fully trimethylated product H3K9me3. We did not detect any formation of mono-, di- or trimethylated ornithine in the presence of G9a/GLP and SAM (Fig. 3C and D, middle panels). On the other hand, we found that G9a and GLP produce traces (<15%) of monomethylated product H3hK9me; with G9a, a trace of dimethylated H3hK9me2 was also observed (Fig. 3C and D, bottom panels). Use of increased amounts of G9a/GLP (10 μM) and SAM (1 mM) did not enable productive methylation reactions with H3Orn9 either within 1 hour or 6 hours (Supplementary Figures S26–S27). However, such conditions resulted in the significant increase in the formation of H3hK9me and H3hK9me2 in the presence of G9a (and to a lesser extent by GLP) after 1 hour at 37 °C (Supplementary Figures S28–S29); possibly due to a slightly increased activity of the batch of recombinantly expressed G9a over GLP. Remarkably, we observed a high degree of the formed H3hK9me2, along with small amounts of monomethylated product H3hK9me and unreacted starting peptide H3hK9, after 6 hour incubation at 37 °C (Supplementary Figures S28–S29). In control experiments, H3hK9 (100 μM) was incubated with SAM (1 mM) for 1 hour and 6 hours at 37 °C; we did not detect any H3hK9me or H3hK9me2, implying that G9a and GLP are required (at 10 μM) for the methylation reactions (Supplementary Figure S30).

In order to verify that ornithine- and homolysine-containing peptides bind to G9a, we also carried out competition experiments between the 14-mer H3K9 (residues 1–14) and 15-mer H3Orn9/H3hK9 (residues 1–15) histone peptides; 14-mer H3K9 is an excellent substrate for G9a, and is trimethylated under standard assay conditions (Supplementary Figure S31). A significant decrease in the methylated product formation for 14-mer H3K9 was observed in the presence of equimolar amounts of H3Orn9; instead of H3K9me3 that was detected in the absence of H3Orn9, we observed a mixture of H3K9, H3K9me and H3K9me2 (Supplementary Figure S31). Similarly, H3hK9 inhibited G9a-catalyzed methylation of 14-mer H3K9, yielding predominantly monomethylated and dimethylated lysine; notably, no H3hK9me was observed (Supplementary Figure S31). Collectively, these results highlight that H3Orn9 and H3hK9 do bind to G9a and act as H3K9 competitive inhibitors of G9a.

Having shown that G9a and GLP have the ability to catalyze methylation of homolysine-containing histone H3hK9, we carried out additional experiments with tumor suppressor p53, another well-established non-histone substrate for KMTs^22–24^. The incubation of p53 peptides (residues 365–379), which possess Lys, Orn and hLys at position 373, in the presence of G9a/GLP and SAM for 3 hours at 37 °C revealed that ornithine is not methylated at all, whereas lysine and homolysine underwent monomethylation and dimethylation reactions (Supplementary Figures S32–S33). The comparision of abundancies of unreacted substrates along with the monomethylated and dimethylated products indicates that lysine is indeed a better substrate than homolysine for G9a/GLP-catalyzed methylations of p53 peptides. In addition, we examined p53 peptides that possess Lys, Orn and hLys at position 372 for SETD7-catalyzed reactions. Although lysine underwent monomethylation reaction in the presence of SETD7 and SAM, we detected only traces (<2%) of monomethylated homolysine and no methylation reaction on ornithine under same assay conditions (Supplementary Figure S34).

Taken together, our MALDI-TOF MS analyses reveal that lysine exhibits the optimal length of the side chain required for productive KMT catalysis. The one-methylene shorter ornithine was not methylated at all by the panel of four human KMTs under various conditions, whereas we observed that G9a and GLP have a potential to accept the one-methylene longer homolysine as a substrate, both when present on histone and p53 peptides.

### NMR assays for KMT-catalyzed methylation

**Figure 4:**
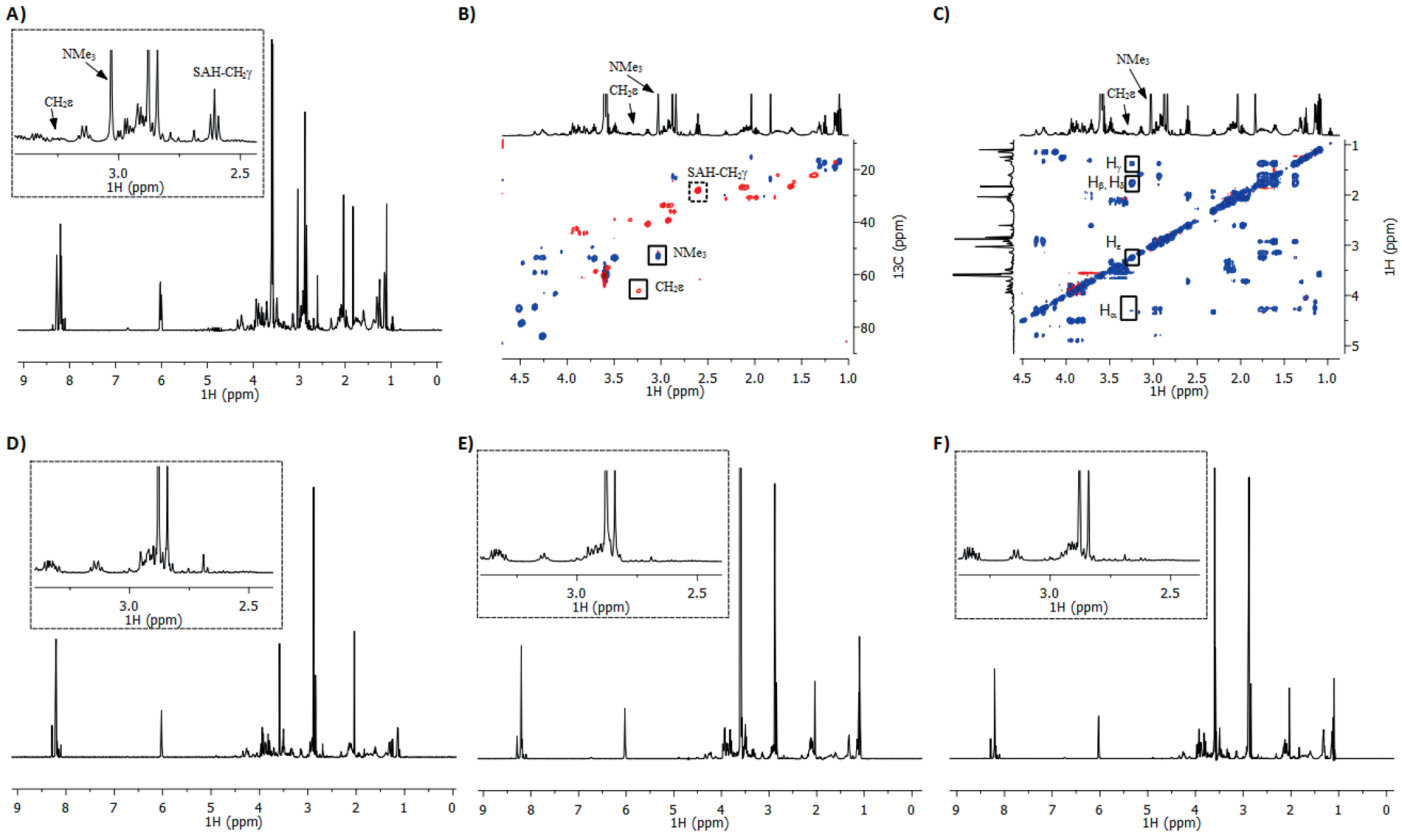
NMR analyses of GLP-catalyzed methylation. (**A**) ^1^H NMR data and the assignment of indicative resonances of the GLP-catalyzed methylation of H3K9 in the presence of SAM after 1 hour at 37 °C. The zoomed area is shown in dotted box. (**B**) ^1^H-^13^C HSQC data with the assignment of important cross-peaks. (**C**) TOCSY data with the assignment of H-H cross-peaks. (**D**) Control experiments showing the incubation of H3K9 and SAM in the absence of GLP. (**E**) ^1^H NMR data showing that GLP does not catalyze methylation of H3Orn9 in the presence of SAM after 1 hour at 37 °C. The zoomed area is shown in dotted box. (**F**) ^1^H NMR data showing that GLP does not catalyze methylation of H3hK9 in the presence of SAM after 1 hour at 37 °C. The zoomed area is shown in dotted box.

Having shown that H3K9 peptide underwent quantitative trimethylation in our MALDI-based assay, we then carried out more detailed NMR spectroscopic analyses on GLP-catalyzed trimethylation of the H3K9 peptide (Fig. 4). Prior to the NMR analyses of enzymatic trimethylation of H3K9, we performed complete analyses of H3K9, SAM and SAH to identify the key changes in the NMR spectra of enzymatic reaction (Supplementary Figures S35–S42). 1D and 2D NMR data reveal that the resonances at 4.31 ppm (CHα), 2.92 ppm (CH_2_ε), 1.79 ppm (CHβ), 1.71 ppm (CHβ), 1.62 ppm (CH_2_δ) and 1.37 ppm (CH_2_γ) ppm correspond to lysine residues of H3K9. The appearance of characteristic new resonances at 3.25 ppm (m) and 3.03 ppm (s) in the ^1^H NMR spectrum of the reaction mixture that contains GLP (8 μM), H3K9 peptide (400 μM) and SAM (2 mM) in Tris-D_11_ buffer (50 mM, pD 8.0) after 1 hour at 37 °C revealed that H3K9 peptide underwent trimethylation of K9 (Fig. 4A). The subsequent analysis of the multiplicity-edited ^1^H- ^13^C HSQC (Heteronuclear Single Quantum Correlation) spectrum revealed that the ^1^H resonance at 3.25 ppm (^13^C: 66.2 ppm) is lysine CH_2_ε, the result that is consistent with the previous report on SETD7 variant-catalyzed trimethylation of H3K4 peptide^25^, and that the singlet at 3.03 ppm is either a CH or CH_3_ group (Fig. 4B). The integral ratio between the peaks at 3.25 ppm and 3.03 ppm were ~2:9, suggesting that the peak at 3.03 ppm represents N(CH_3_)_3_. This was confirmed by HMBC (Heteronuclear Multiple Bond Correlation), which revealed a correlation between the singlet at 3.03 ppm and the ^13^C of CH_2_ε (66.2 ppm), and thus additionally verified the assignment of N(CH_3_)_3_ (Supplementary Figure S43). The TOCSY (Total Correlation Spectroscopy) analysis starting at Hε at 3.25 ppm revealed the Hδ (1.79 ppm), Hγ (1.38 ppm), Hβ (1.79 ppm) and Hα (4.32 ppm) resonances of the rest of the trimethylated lysine chain (Figs 4C and Supplementary Figure S43). To provide unambiguous evidence for the trimethylation of H3K9 by GLP, we also synthesized H3K9me3 and characterized it by 1D and 2D NMR spectroscopy to compare the spectroscopic data with enzymatically produced H3K9me3. The characteristic proton resonances of chemically synthesized H3K9me3 have identical chemical shifts to those seen for enzymatically produced product (Supplementary Figure S44). Comparison of 1D and 2D TOCSY data of enzymatic and chemically synthesized H3K9me3 also showed identical coupling network for the trimethylated lysine residue (Supplementary Figure S45); this was further confirmed by comparing the ^1^H- ^13^C HSQC and HMBC correlations (Supplementary Figures S45–S46).

In addition to monitoring the characteristic changes on the lysine of H3K9, we were also interested in identifying the additional resonances that arise from the enzymatic conversion of cosubstrate SAM to SAH. The new resonances in the anomeric and aliphatic region at 6.01 (d) and 2.61 (t) ppm were assigned as the methine and methylene of SAH by NMR analysis (Fig. 4A). The methylene protons next to sulphur of SAM experience especially large ^1^H and ^13^C upfield chemical shift changes upon demethylation: the CH_2_γ resonance appears at 3.56 ppm (ddd) and 3.36 ppm (ddd) for SAM, whereas SAH-CH_2_γ appears at 2.61 ppm as a triplet. The multiplicity and chemical shifts of SAH that was produced by the enzymatic process are in agreement with the spectroscopic data of the authentic sample of SAH. ^1^H NMR analysis also indicates that the conversion of SAM to SAH is strongly coupled to the GLP-catalyzed trimethylation of H3K9 (Supplementary Figure S47).

We then performed a control experiment to demonstrate that the methylation of lysine is an enzymatic process; under standard conditions but in the absence of GLP, we did not observe indicative resonances for trimethyllysine at 3.25 and 3.03 ppm and also for SAH at 6.01 and 2.61 ppm in the ^1^H NMR spectrum (Fig. 4D); this was further supported by TOCSY and HSQC analyses (Supplementary Figure S48). The conclusions of the control experiment were complemented by the MALDI analysis; in both cases, it was clearly demonstrated that trimethylation of H3K9 and conversion of SAM to SAH are enzymatic processes.

After validating that the H3K9 peptide with the natural target sequence is an excellent substrate for GLP, we examined by NMR if the enzymatic methylation of two unnatural lysine analogues, Orn and hLys, would occur. The absence of characteristic resonances for N(CH_3_)_3_ at ~3.00 ppm, the downfield resonance at ~3.25 ppm that corresponds to CH_2_N, and a triplet for SAH-CH_2_γ at 2.61 ppm, in the ^1^H NMR spectrum of the reaction mixture that contains GLP (8 μM), H3Orn9/H3hK9 peptide (400 μM) and SAM (2 mM) in Tris-D_11_ buffer (50 mM, pD 8.0) after 1 hour at 37 °C implies that ornithine and homolysine are not methylated under standard NMR conditions (Figs 4E–F and Supplementary Figures S49–S50). A longer incubation (6 hours) of H3hK9 in the presence of larger amounts of GLP (15 μM), however, led to the formation of H3hK9me2 and SAH, as indicated by new resonances at 2.79 ppm (NMe_2_), 3.05 ppm (CH_2_ζ) and 2.60 ppm (CH_2_γ of SAH) (Supplementary Figure S51). Additional NMR analysis of the multiplicity-edited HSQC spectrum reveals that the ^1^H resonance at 3.05 ppm (^13^C: 57.7 ppm) is hLys CH_2_ζ; this was further confirmed by HMBC analysis, which shows a correlation between the singlet at 2.79 ppm (NMe_2_) and the ^13^C of CH_2_ζ (57.7 ppm) (Supplementary Figures S52–S53). Our observed chemical shifts for NMe_2_ and CH_2_ζ match very well with the previously reported NMe_2_ and CH_2_ε shifts of LysMe_2_ within 0.3 ppm in ^13^C (observed: CH_2_ζ (60.2), NMe_2_ (45.3); literature^25^: CH_2_ε (60.5), NMe_2_ (45.5)) after adjusting our ^13^C chemical shift reference from TMS (tetramethylsilane) to DSS (4,4-dimethyl-4-silapentane-1-sulfonic acid). Overall, our NMR results indicate that ornithine- and homolysine-containing histone peptides are not methylated in the presence of GLP under standard conditions, whereas H3hK9 is predominantly dimethylated in the presence of larger amounts of GLP and prolonged incubation; these results are compatible with our MALDI-TOF analyses. The comparison of anomeric region in the ^1^H NMR spectrum of enzymatic reaction of H3K9 also reveals that the conversion of SAM to SAH is tightly coupled to the enzymatic trimethylation of H3K9 (Supplementary Figure S54).

### Quantum mechanical/molecular dynamics studies on KMT catalysis

**Figure 5:**
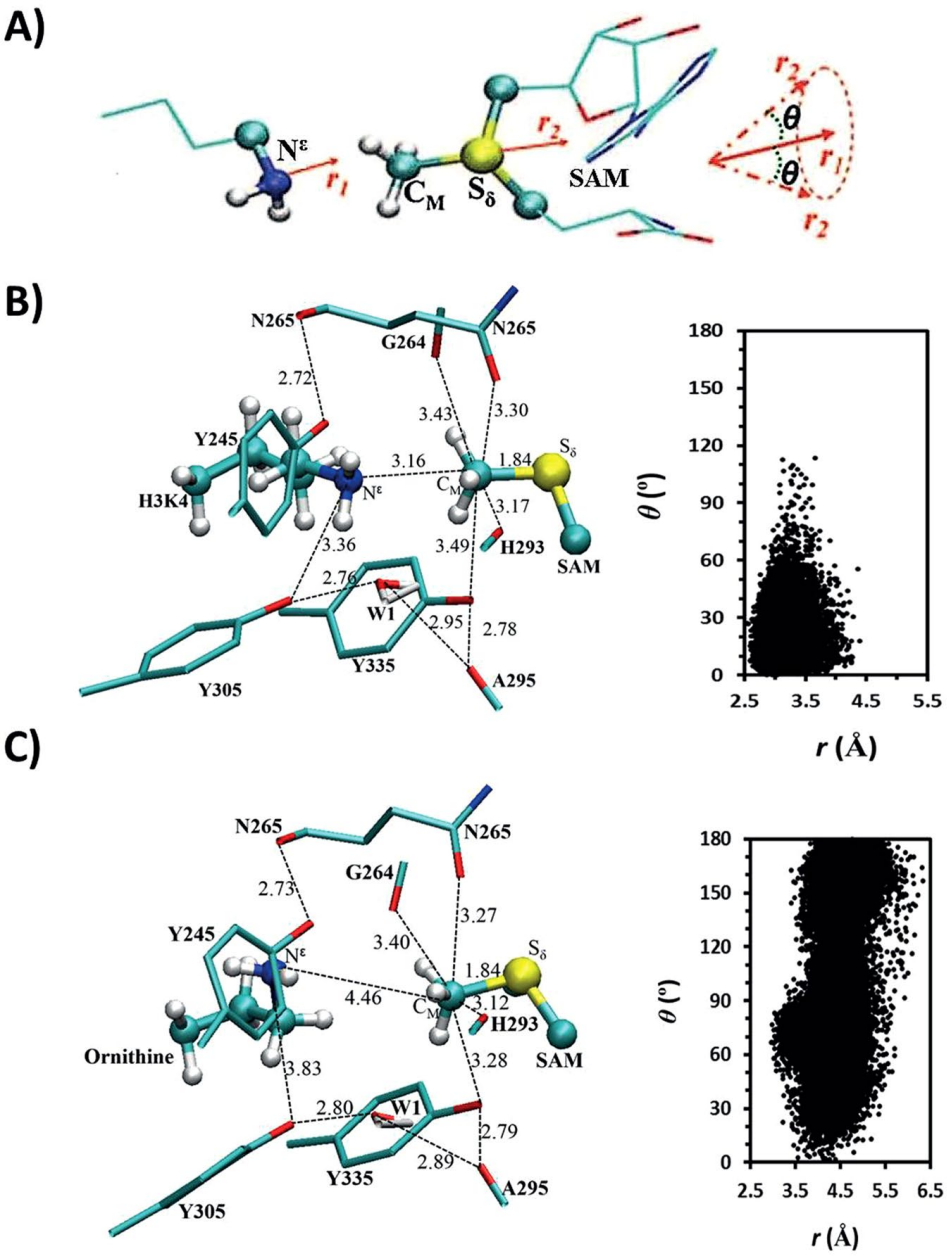
MD results for SETD7. (**A**) The relative orientation of SAM (AdoMet) and lysine in the reactant complex. θ is defined as the angle between the two vectors r_1_ and r_2_. (**B**) Representative active-site structure of the reactant complex of SETD7 for the methylation containing SAM and lysine along with r(C_M_···N_ζ_) and θ distributions obtained from the QM/MM MD simulations. SETD7 is shown in sticks, and SAM and lysine are in balls and sticks. Some average distances from the simulations are also given (in angstroms). (**C**) The active-site structure of the reactant complex of SETD7 containing SAM and ornithine.

**Figure 6:**
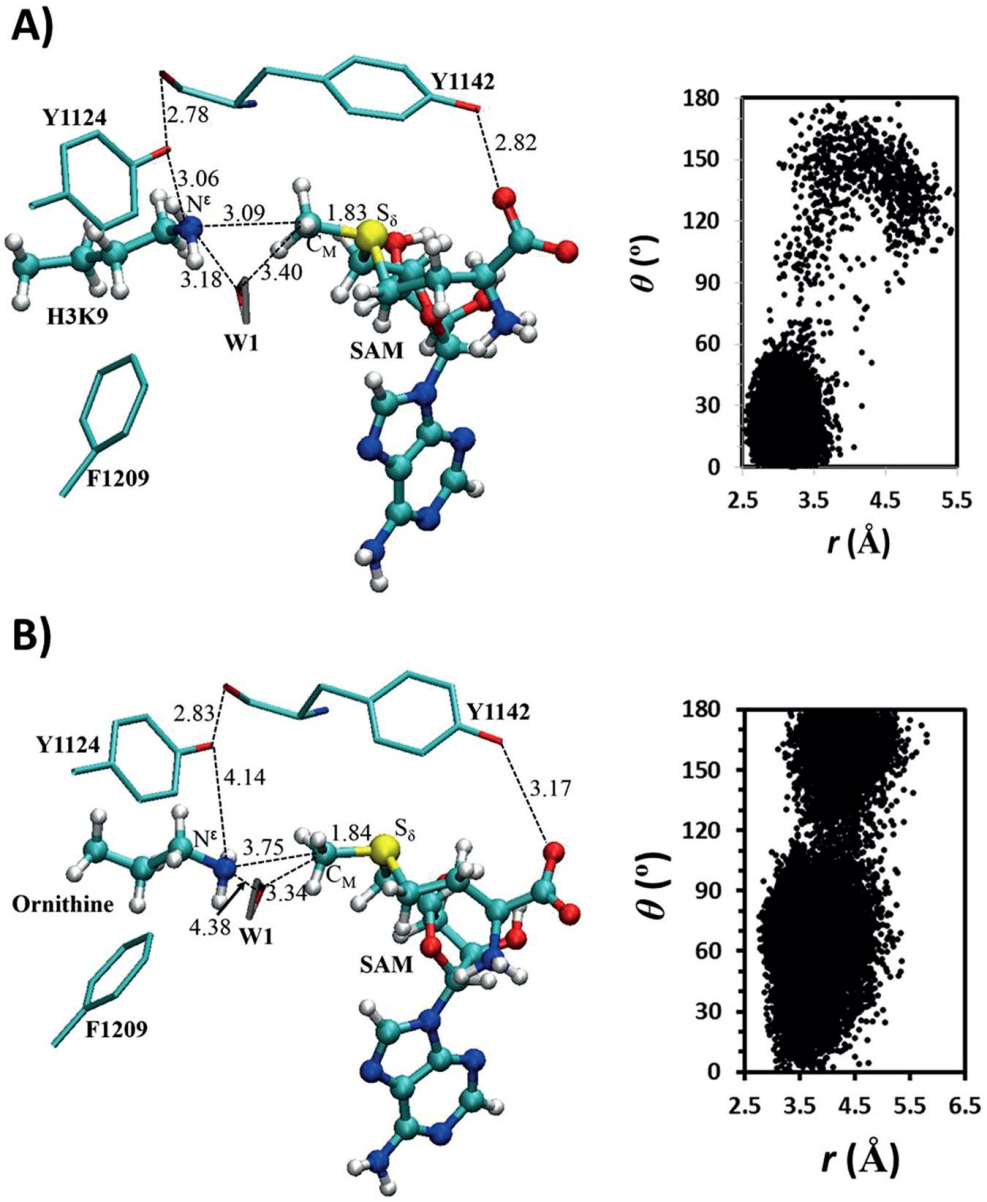
MD results for GLP. (**A**) Representative active-site structure of the reactant complex of GLP containing SAM (AdoMet) and lysine. (**B**) The active-site structure of the reactant complex of GLP containing SAM and ornithine.

To understand the energetic and structural origin of the experimental observations, we also performed quantum mechanical/molecular mechanical (QM/MM) molecular dynamics (MD) and free energy (potential of mean force) simulations based on some existing X-ray structures. The representative active-site structures of the reactant complexes obtained from the simulations for the methylation in SETD7 with the lysine and ornithine substrates are given in Fig. 5B and C, respectively; the distribution map of *r*(C_M_-N_ζ_) and *θ* is also given in each case (Note: instead of N^ε^ labelling in experimental studies, we use a common nomenclature for N_ζ_ in computational studies). As can be seen from Fig. 5B, the lone pair of electrons on N_ζ_ of the target lysine is well aligned with the methyl group of SAM (AdoMet). This is further demonstrated by the large population of the structures with relatively short *r*(C_M_…N_ζ_) distances (e.g., ≤3.5 Å) and small values of the *θ* angle (e.g., ≤30°). The average distance between N_ζ_ and C_M_ of the methyl group is approximately 3.2 Å and the *θ* angle is primarily within the range of 0–60°. Fig. 5C shows that for the ornithine substrate the lone pair of electrons cannot be well aligned with the methyl group, with a longer average *r*(C_M_-N_ζ_) distance of about 4.5 Å. Moreover, the side chain of ornithine became quite flexible with the *θ* angle changing between 0° and 180°. This is probably due in part to the loss of the hydrogen bond involving Y305 with ε-amino group of ornithine; i.e. the active conformation of ε-amino group is no longer stabilized by this interaction. Y305 is known to be crucial for the activity of SETD7, and the present result seems to support the suggestion that it may play a role in stabilizing the reactive configuration. The representative active-site structures of the reactant complexes for the methylation in GLP with the lysine and ornithine substrates are given in Fig. 6A and B, respectively. Similar to the cases involving SETD7, while the lone pair of electrons on N_ζ_ of the target lysine is well aligned with the methyl group of SAM, this is not the case for the ornithine substrate, which shows a wide distribution of the *θ* angle and a relatively longer average distance for *r*(C_M_-N_ζ_). Notably, the hydrogen bond interactions involving Y1124 and the active site water were broken for the ornithine complex.

**Figure 7:**
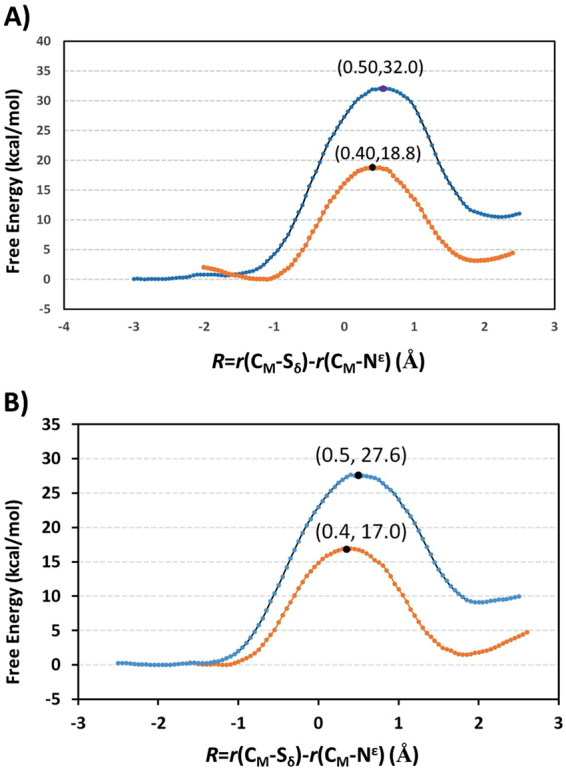
Free energy (potential of mean force) profiles for the methylation reactions in the enzymes as a function of the reaction coordinate [R = r(C_M_···S_δ_) − r(C_M_···N_ζ_)]. (**A**) The free energy profile for methylation of lysine in SETD7: orange line with a free energy barrier of 18.8 kcal mol^−1^ and the location of the transition state is at around 0.4. The free energy profile for methylation of ornithine in SETD7: blue line with a free energy barrier of 32 kcal mol^−1^. (**B**) The free energy profile for methylation of lysine in GLP: orange line with a free energy barrier of 17.0 kcal mol^−1^. The free energy profile for methylation of ornithine in GLP: blue line with a free energy barrier of 27.6 kcal mol^−1^.

The free-energy profiles for methylation in SETD7 and GLP are plotted in Fig. 7A and B, respectively. The free energy barrier for the SETD7-catalyzed methyl transfer with the lysine substrate obtained here is quite close to our earlier results (17–18 kcal mol^−1^)^26^. It should be pointed out that in the earlier studies^26–29^ a scaling procedure was used to correct the errors with the previous SCC-DFTB parameters. The similarity of the free energy results reported here suggests that the new DFTB3 parameters used here are more accurate in producing free energy data and a scaling procedure may not be necessary. Fig. 7A shows that the free energy barrier with the ornithine substrate is considerably higher than that with the lysine substrate (Orn: 32.0 kcal mol^−1^ vs Lys: 18.8 kcal mol^−1^), suggesting that the enzyme would not be active on the ornithine substrate, the result that is in line with our experimental observations. A similar discussion can be made for the GLP-catalyzed monomethylation. Indeed, the results of the free energy simulations shown in Fig. 7B demonstrated that GLP would be significant less active in catalyzing monomethylation for the ornithine substrate compared to that for the lysine substrate (Orn: 27.6 kcal mol^−1^ vs Lys: 17.0 kcal mol^−1^). Since the enzyme could not add even the first methyl group to ornithine, dimethylornithine may not be produced by GLP as well.

It should be mentioned that although the structures of the reactant complexes with lysine and ornithine are very different, the structures near transition state obtained from the free energy simulations (Supplementary Figure S55) are quite close to each other with rather similar interactions. Thus, the failure of SETD7 and GLP to catalyze the ornithine methylation may be related to the inability of the enzyme to bind the ornithine substrate in a reactive conformation for methylation. This seems also to be the case, at least in part, for some homolysine complexes. Indeed, we have tried to generate the models with the homolysine substrate based on the X-ray structures and through increasing the length of the lysine chain. The enzyme complexes generated in such ways had always some poor contacts with (or were too close to) SAM, some of the active site residues (e.g. Y245 and Y305 in SETD7) or the active site water molecule (Supplementary Figures S56–S57). The steric repulsions from such poor contacts would likely make it difficult to form the reactive (near attack) conformation (as observed in the SETD7 and GLP complexes with the lysine substrate) for the efficient methyl transfer to occur.

## Discussion

Recent studies in the epigenetic framework have demonstrated that the state-of-the-art chemical approaches provide essential insights into understanding the fundamentals of vital epigenetic processes^30–33^. Our synergistic experimental and computational studies on human KMTs reported here exemplify the power of modern chemical tools in addressing the posttranslational modifications of histones. The examination of histones that possess lysine and its simplest shorter and longer analogues reveals that lysine exhibits the perfect length of the side chain for SAM-dependent KMT-catalyzed methylations. Out of four human KMTs used in this study, only GLP and G9a were able to catalyze methylation of longer homolysine in histone (poor substrate) and p53 (relatively good substrate) peptides, whereas none of the enzymes has the ability to methylate the shorter ornithine. Combined MALDI-TOF MS and NMR studies confirmed that KMT-catalyzed histone methylation is tightly coupled to the enzymatic conversion of SAM to SAH. Our experimental results were further supported by quantum mechanical/molecular dynamics studies, which provided rationale into the lack of observed methylation of ornithine. We attribute the lack of ornithine methylation to its inability to bind in the active site of KMT in a reactive conformation. On the other hand, homolysine seems to associate with enzymes in a way that either sterically clashes with the active site residues or with SAM cosubstrate, thus leading to an inadequate conformation for efficient methyl transfer reactions.

Collectively, this work highlights that histone lysine methyltransferases are highly specific epigenetic enzymes that are very sensitive to the length of the side chain of residues that undergo N-methylation reactions. In this regard, it is noteworthy that KMTs (and related enzymes) do have an ability to accept several simple SAM analogues as cosubstrates for alkylation reactions, suggesting different levels of promiscuity of SAM- and lysine-binding pockets^34–36^. In contrast to the examination of SAM analogues, a chemically diverse panel of lysine analogues has not yet been explored for KMT catalysis. The cysteine-derived lysine has been observed to be methylated by SUV39H1, a H3K9 methyltransferase, suggesting that the cysteine-derived analogue is a good functional mimic of natural lysine^37^. These observations along with our results demonstrate that histone lysine methyltransferases could, in principle, have an ability to methylate residues other than natural lysine. It is envisioned that future explorations of the substrate scope for KMT catalysis will importantly contribute to our understanding of epigenetic processes that play essential roles in human health and disease.

## Methods

### Enzyme production

#### Cloning, expression and purification of human SETD7

The expression and purification of SETD7 (residues 1–366) was performed as previously described^16^. In brief, His-tagged SETD7 was expressed in *E. coli* Rosetta BL21 (DE3)pLysS cells in the presence of 0.4 mM IPTG (final concentration) and cultured overnight at 16 °C. The cells were pelleted and resuspended in lysis phosphate buffer saline (PBS) supplemented with 250 mM NaCl, 5 mM imidazole, 5% glycerol and 2 mM β-mercaptoethanol and subsequently lysed by sonication. The cell lysate was then incubated with Ni-NTA beads for 2.5 h at 4 °C. The beads were washed with 50 mL column volumes of lysis buffer containing 20 mM Tris pH 8.0, 250 mM NaCl, 50 mM imidazole and 5% glycerol, and the protein was subsequently eluted with 20 mM Tris pH 8.0, 250 mM NaCl, 250 mM imidazole, and 5% glycerol. The eluted protein was concentrated using a spinfilter device (Amicon, 3.5 MWCO) and was further purified by gel filtration chromatography, using a Superdex 75 column and 200 mM Tris pH 7.5, 20 mM NaCl at 0.5 mL min^−1^ flow speed.

#### Cloning, expression and purification of human SETD8

The expression and purification of SETD8 (residues 186–352) was carried out by following a published protocol^19^. Briefly, His-tagged SETD8 was expressed in *E. coli* Rosetta BL21 (DE3)pLysS cells in the presence of 1.0 mM IPTG (final concentration) and cultured overnight at 16 °C. Harvested cells were re-suspended into 20 mM Tris pH 7.8, 500 mM NaCl, 5 mM imidazole, 5% glycerol, and 5 mM β-mercaptoethanol, and subsequently lysed by sonication. The cell lysate was then incubated with Ni-NTA beads and the beads were then washed with 20 mM Tris pH 7.8, 500 mM NaCl, 20 mM imidazole and 5% glycerol, and the protein was subsequently eluted with 20 mM Tris pH 7.8, 500 mM NaCl, 250 mM imidazole and 5% glycerol. The eluted protein was concentrated using a spinfilter device (Amicon, 3.5 MWCO) and was further purified by gel filtration chromatography, using a Superdex 75 column and 20 mM Tris pH 7.5, 100 mM NaCl and 10 mM β-mercaptoethanol at 0.5 ml min^−1^ flow speed. Fractions with correct molecular weight and best purity based on SDS-PAGE were pooled and concentrated, aliquoted, snap-frozen, and stored at −80 °C.

#### Cloning, expression and purification of human GLP

The expression and purification of GLP (residues 951–1235) was carried out by following a reported protocol^18^. DNA sequence of catalytic domains of human GLP (951–1235, in pET28a-LIC vector) was used for the expression. Briefly, His-tagged GLP was expressed in *E. coli* Rosetta BL21 (DE3)pLysS cells in the presence of 0.1 mM IPTG (final concentration) and 0.1 mM of zinc chloride, and cultured overnight at 16 °C. Harvested cells were resuspended in lysis buffer (25 mM NaCl, 2 mM β-mercaptoethanol, PBS buffer pH 7.4, 5% glycerol, 0.1% Triton X-100). The cell lysate was then incubated with Ni-NTA beads and the beads were then washed with 20 mM Tris pH 8.0, 250 mM NaCl, 50 mM imidazole, and 5% glycerol. The protein was eluted with elution buffer (20 mM Tris pH 8.0, 250 mM NaCl, 250 mM imidazole, and 5% glycerol). The eluted protein was concentrated using a spinfilter device (Amicon, 3.5 MWCO) and was further purified by gel filtration chromatography using the AKTA system, employing a Superdex 75 column equilibrated with 20 mM Tris pH 8.0, 150 mM NaCl at 0.5 ml min^−1^ flow speed. Fractions were monitored by SDS-PAGE and those containing protein of the correct mass and high purity were pooled and concentrated, aliquoted, flash-frozen in liquid nitrogen, and stored at −80 °C.

#### Cloning, expression and purification of human G9a

The expression and purification of G9a (residues 913–1193) was carried out using the same protocol as for GLP^18^, except that G9a expression was induced by adding 1.0 mM IPTG and 0.1 mM of zinc chloride. DNA sequence of catalytic domains of human G9a (913–1193 residues, in pET28a-LIC vector) was used for the expression.

#### Solid-phase peptide synthesis

Synthetic peptides of H3, H4 and p53 were prepared manually using standard Fmoc-based solid-phase peptide chemistry on a cartridge (6 mL, Screening Devices B.V., The Netherlands). Breipohl rink amide resin was employed with H3 to afford peptides with the C-terminal amide and Wang resin was used with H4 and p53 to afford peptides with the C-terminal carboxylic acid. Coupling conditions for the amino acids: 3.0 equiv of Fmoc-amino acid, 3.6 equiv. of 1 M HOBt in DMF, and 3.3 equiv. of 1 M DIPCDI in DMF. The latter three components were premixed for 2 min and were added to the resin. The mixture was shaken for 1 h to ensure efficient coupling. All unnatural amino acids were incorporated into position 4 and position 9 of 1–15 H3 peptides and at position 20 for 13–27 H4 peptide with elongated reaction time of 16 h at room temperature. The Fmoc protecting group was removed by agitation with piperidine in DMF (20%) for 30 min. The coupling reactions and Fmoc deprotections were monitored with the colour Kaiser test. After synthesis, peptides were cleaved and deprotected from the resin by treatment with 95.0% trifluoroacetic acid/2.5% triisopropylsilane/2.5% water at room temperature for 4 h, followed by precipitation with cold diethyl ether. Crude peptides were purified by reverse phase HPLC. Fractions containing the pure peptide were combined, frozen in liquid nitrogen, and lyophilised to afford the product as a white-off solid with high purity as confirmed by analytical HPLC. Predicted masses were confirmed by MALDI-TOF MS, LC-MS, and ESI-MS. Peptides were prepared as TFA salts.

#### MALDI-TOF MS assays

The methyltransferase activity assays were performed by MALDI-TOF MS. The assay mixture contained the enzyme (2 µM), histone peptide (100 µM), SAM (200 μM for monomethylation and 500 μM for trimethylation), buffered in 50 mM Tris at optimal pH 8.0 (100 µL final volume). Samples were incubated in an Eppendorf vial 1.5 mL in thermomixer for 1 h at 37 °C. The assay mixtures of p53 peptide (100 µM), SAM (2 mM) and G9a/GLP/SETD7 (10 µM) were incubated for 3 h at 37 °C. A 5 μL aliquot of the reaction mixture was quenched with 5 µL of MeOH to stop the enzymatic reaction before analysis by MALDI-TOF MS. The spots were placed on a stainless steel MALDI plate (MS 96 target ground steel BC of Bruker, Germany). The mass spectra were measured in the positive reflector mode using α-cyano-4-hydroxycinnamic acid matrix. The mass corresponding to monomethylation observed as +14 Da, dimethylation observed as +28 Da, and trimethylation observed as +42 Da. Negative controls without enzyme and without SAM were included. The MALDI-TOF MS data were annotated employing FlexAnalysis software (Bruker Daltonics, Germany). All methylation experiments were carried out in duplicate.

#### NMR assays

NMR enzymatic experiments were conducted at 310 K in 50 mM Tris-D_11_.HCl (pD 8.0). Typically, to a premixed solution of GLP (8 μM) and SAM (2 mM), was added H3K9/H3Orn9/H3hK9 peptide (400 μM). After 1 h incubation at 37 °C in an Eppendorf vial, the reaction mixture was transferred into the NMR tube and then diluted to 500 μL and recorded by ^1^H NMR at 298 K. NMR spectra were recorded on a Bruker Avance III spectrometer paired with a 500 MHz magnet equipped with the Prodigy BB cryoprobe. ^1^H 1D spectra were acquired using presaturation to suppress the water signal with 128 or 256 transients and a relaxation delay of 4 s. 2D TOCSY spectra were acquired with presaturation of the water resonance using 1k points per transient, 8.3 kHz spin-lock for 100 ms, 56 transients per increment with a relaxation delay of 2 s and 512 increments with a sweep width of 10 ppm in each dimension. 2D ^1^H- ^13^C multiplicity-edited HSQC spectra were acquired using 1k points per transient, 64 transients per increment, a relaxation delay of 2 s, and 512 increments. The ^13^C sweep width spanned from −10 ppm to 130 ppm. ^1^H NMR characterization of substrates prior to enzymatic catalysis was performed using a 30° excitation pulse, 16–128 transients per compound, and a relaxation delay of 8 s. ^1^H- ^13^C spectra of the substrates were recorded using a 30° excitation pulse, 512–4096 transients per compound and a relaxation delay of 2 s. ^1^H and ^13^C chemical shifts were externally referenced to TMS based on the lock frequency of solvent. Adjustment of the referencing scale from TMS to DSS for comparison to literature reports was performed post-acquisition using the Absolute Referencing feature in MestreNova.

#### QM/MM computations

QM/MM free energy (potential of mean force) and MD simulations were performed to study the active-site dynamics of SETD7 and GLP and to calculate the free energy profiles of the methyl transfers from SAM (AdoMet) to the ε-amino group of the target lysine/ornithine residues using the CHARMM program^38^. The –CH_2_–CH_2_–S^+^(Me)–CH_2_– part of SAM and lysine/ornithine side chains were treated by QM and the rest of the system by MM. The link-atom approach^39^ was applied to separate the QM and MM regions. A modified TIP3P water model^40^ was employed for the solvent, and the stochastic boundary molecular dynamics method^41^ was used for the QM/MM simulations. The reaction region was a sphere with radius *r* of 20 Å, and the buffer region extended over 20 Å ≤ *r* ≤ 22 Å. The resulting systems contained around 5500 atoms, including about 800–900 water molecules. The DFTB3 method^40,41^ was used for the QM atoms, and the all-hydrogen CHARMM potential function (PARAM27)^42^ was used for the MM atoms. The initial coordinates for the reactant complexes of the methylation were based on the crystallographic complexes (PDB codes: 1O9S and 3HNA for SETD7 and GLP, respectively) containing, SAH (AdoHcy) and methyl lysine (i.e., the product complexes). In each of the cases, a methyl group was manually added to AdoHcy to change it to AdoMet and the methyl group(s) on the methyl lysine were manually deleted to generate the target lysine. For the models with the ornithine substrate, the ε-amino group on lysine was removed first, and the CH_2_ group next to N_ζ_ on the lysine chain was changed to N_ζ_H_2_. The enzyme complexes with homolysine generated always had some bad contacts, and no simulations were performed with in such cases. The initial structures for the entire stochastic boundary systems were optimized using the steepest descent (SD) and adopted-basis Newton-Raphson (ABNR) methods. The systems were gradually heated from 50.0 to 298.15 K and 1-fs time step was used for integration of the equation of motion. 1.5 ns QM/MM MD simulations were carried out for each of the reactant complexes; the distribution maps of *r*(C_M_-N_ζ_) and *θ* were also generated. The umbrella sampling method^43^ implemented in the CHARMM program along with the Weighted Histogram Analysis Method (WHAM)^44^ was applied to determine the change of the free energy (potential of mean force) as a function of the reaction coordinate for the methyl transfer from SAM to the target lysine/ornithine in SETD7 and GLP. The reaction coordinate was defined as a linear combination of *r*(C_M_-N_ζ_) and *r*(C_M_-S_δ_) [*R* = *r*(C_M_-S_δ_) − *r*(C_M_-N_ζ_)]. Twenty-six (twenty-five) and thirty-two (thirty) windows were used for SETD7 (GLP) with lysine and ornithine substrates, respectively. For each window 50 ps production runs were performed after 50 ps equilibration. The force constants of the harmonic biasing potentials used in the PMF simulations were 50–400 kcal mol^–1^ Å^–2^.

### Data Access Statement

All data supporting this study are provided as Supplementary Information accompanying this paper.

## Electronic supplementary material

Supplemenary Information
