# Supplementary material for: Lysine Possesses the Optimal Chain Length for Histone Lysine Methyltransferase Catalysis
Source: Sci Rep. 2017 Nov 23;7:16148. doi: 10.1038/s41598-017-16128-4 (PMC5700997; doi:10.1038/s41598-017-16128-4)
Supplement: Supplementary file 1 — Supplemenary Information [file 41598_2017_16128_MOESM1_ESM.pdf]

Supplementary Information

Lysine Possesses the Optimal Chain Length for Histone Lysine Methyltransferase Catalysis

Abbas H. K. Al Temimi[1], Y. Vijayendar Reddy[1], Paul B. White[1], Hong Guo[2,3] , Ping Qian[4] & Jasmin Mecinović[1]

[1] Institute for Molecules and Materials, Radboud University, The Netherlands

[2] Department of Biochemistry and Cellular and Molecular Biology, University of Tennessee, USA

[3] UT/ORNL Center for Molecular Biophysics, Oak Ridge National Laboratory, USA

[4] Chemistry and Material Science Faculty, Shandong Agricultural University, P.R. China

Correspondence and requests for materials should be addressed to J.M. (email: j.mecinovic@science.ru.nl) and P.Q. (email: qianp@sdau.edu.cn).

Table of Contents

Pages

1. General methods S3

2. Analytical HPLC and ESI-MS figures S5

3. MALDI supplementary figures S21

4. NMR supplementary figures S33

5. Computational figures S53

1. General methods

1.1 Materials

All reagents were obtained from commercial sources and used without further purifications. Fmoc amino acid derivatives, N,N'-Disopropylcarbodiimide (DIPCDI), and 1-Hydroxybenzotriazole (HOBt) were obtained from Novabiochem (EMD Chemicals, Gibbstown, USA) or Bachem. Fmoc-L-HLys(Boc)-OH was purchased from Iris Biotech (Germany). Fmoc-Orn(Boc)-OH, Fmoc-Dab(Boc)-OH, and Fmoc-Dap(Boc)-OH, and Breipohl Resin [Fmoc-4-methoxy-4'-(-carboxypropyloxy)-benzhydrylamine linked to Alanyl-aminomethyl] (200-400 mesh) were purchased from Bachem AG (Bubendorf). Triisopropylsilane (TIS), N,N,-diisopropylethylamine (DIPEA), trifluoroacetic acid (TFA), and piperidine were purchased from Sigma-Aldrich. N,N-dimethylformamide (DMF) solvent for peptide synthesis and gradient degree high-performance liquid chromatography (HPLC) acetonitrile were purchased from Actu-All Chemicals b.v (Oss, The Netherlands). *S*-(5′-Adenosyl)-*L*-methionine chloride dihydrochloride (SAM) and S-(5′-Adenosyl)-L-homocysteine (SAH) were purchased from Sigma-Aldrich.

1.2 HPLC purification of HKMTs peptides

Lyophilised crude peptides were purified by prep- HPLC on a Phenomenex® Gemini-NX 3u C18 110A reversed-phase column (150 × 21.2 mm) using gradient elution at constant flow rate of 10 mL/min and the temperature is 30 °C. A typical run for all the natural and unnatural lysine analogues was performed as follows: (C-18 reverse phase column; after 3 mins at 3% a gradient of 3% to 15% over 12 mins was introduced, followed by a gradient of 15% to 30% over 17 mins and from 30% to 100% over 19 mins, proceeding with 100% to 100% over 21 mins finalised by 3 mins at 100% CH_3CN (total runtime 30 mins). Solvent A is 0.1% trifluoroacetic acid in H_2O, Solvent B is 0.1% trifluoroacetic acid in acetonitrile. The pure fractions containing product were combined and freeze-dried overnight to yield the target histone peptides as a white-off solid.

1.3 Analytical HPLC of HKMTs peptides

The Analytic HPLC was performed on a Shimadzu LC-2010A HPLC system (Shimadzu, Kyoto, Japan) on RP C18 column from Phenomenex, Prodigy ODS3, particle size 5 µm, pore size 110 Å, length 150 mm, and internal diameter 4.60 mm. The analysis was performed using solvent gradient of acetonitrile and water (both solvents containing 0.1% TFA) at flow rate 10 mL/min. After 1 min at 5% a gradient of 5% to 100% over 30 min was introduced, followed by 5 min at 100% to 100%. Finally, the system was allowed to re-equilibrate for 14 min. The HPLC signal was recorded at 214 nm absorbance. The retention time of each peptide was shown on the top of the corresponding peak in HPLC chromatogram. The used MilliQ water was purified using a WaterPro PS Polisher (Labconco), set to 18.2 MΩ/cm.

1.4 ESI mass spectrometry of HKMTs peptides

Mass spectra of the HKMTs peptides were confirmed by ESI-MS (Thermo Finnigan LCQ Advantage Max) operating in a positive ionization mode using LC-MS system, which was performed on a Thermo Finnigan LCQ-Fleet ESI-ion trap (Thermofischer, Breda, The Netherlands) equipped with a Phenomenex Gemini-NX C18 column, 50 × 2.0 mm, particle size 3 µM (Phenomenex, Utrecht, The Netherlands). An acetonitrile/water gradient containing 0.1 % formic acid was used for elution (5-100 %, 1-50 min, flow 0.2 mL/min). Ions were scanned in a range of m/z 50-2000 in MS mode. Multiply charged molecular-related ions of each peptide were detected.

2. Analytical HPLC and ESI-MS figures

A)

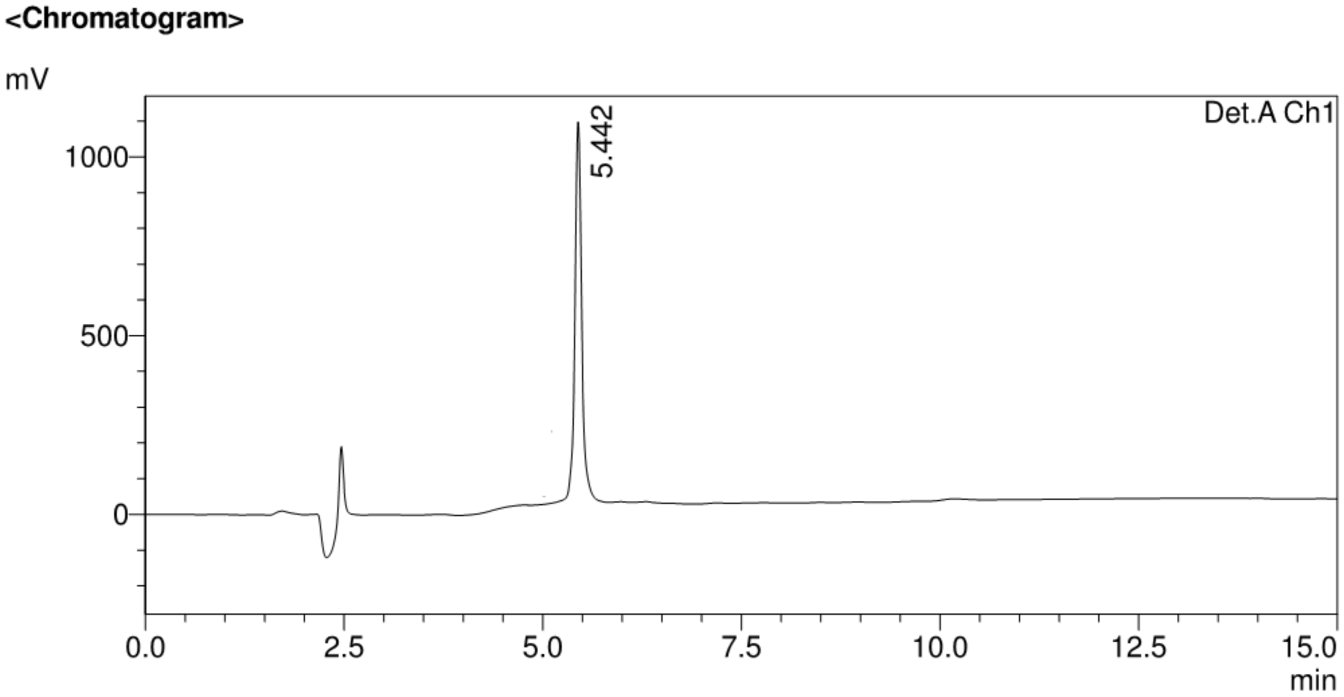

B)

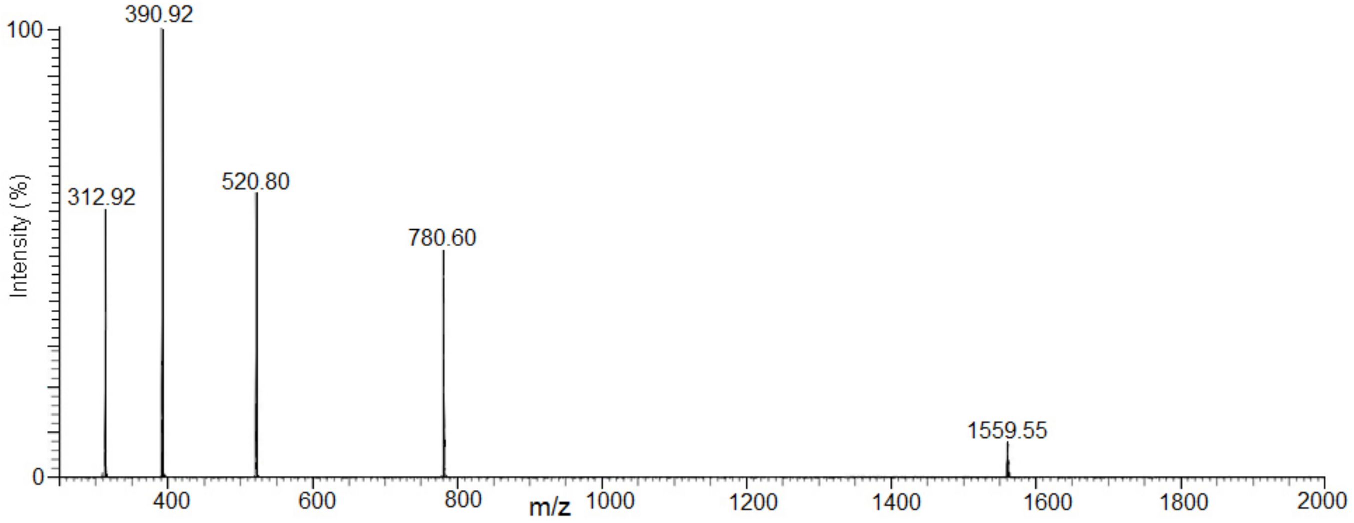

Supplementary Figure S1: A) Analytical HPLC profile and **B**) ESI-MS analysis of $H3_{1\text{-}15}K$ peptide after prep-HPLC purification. It elutes at 5.4 min. Calculated mass is 1559.78 Da, found 1559.55 Da $[M+H]^{1+}$, 780.60 $[M+2H]^{2+}$, 520.80 $[M+3H]^{3+}$, 390.92 $[M+4H]^{4+}$, 312.92 $[M+5H]^{5+}$.

A)

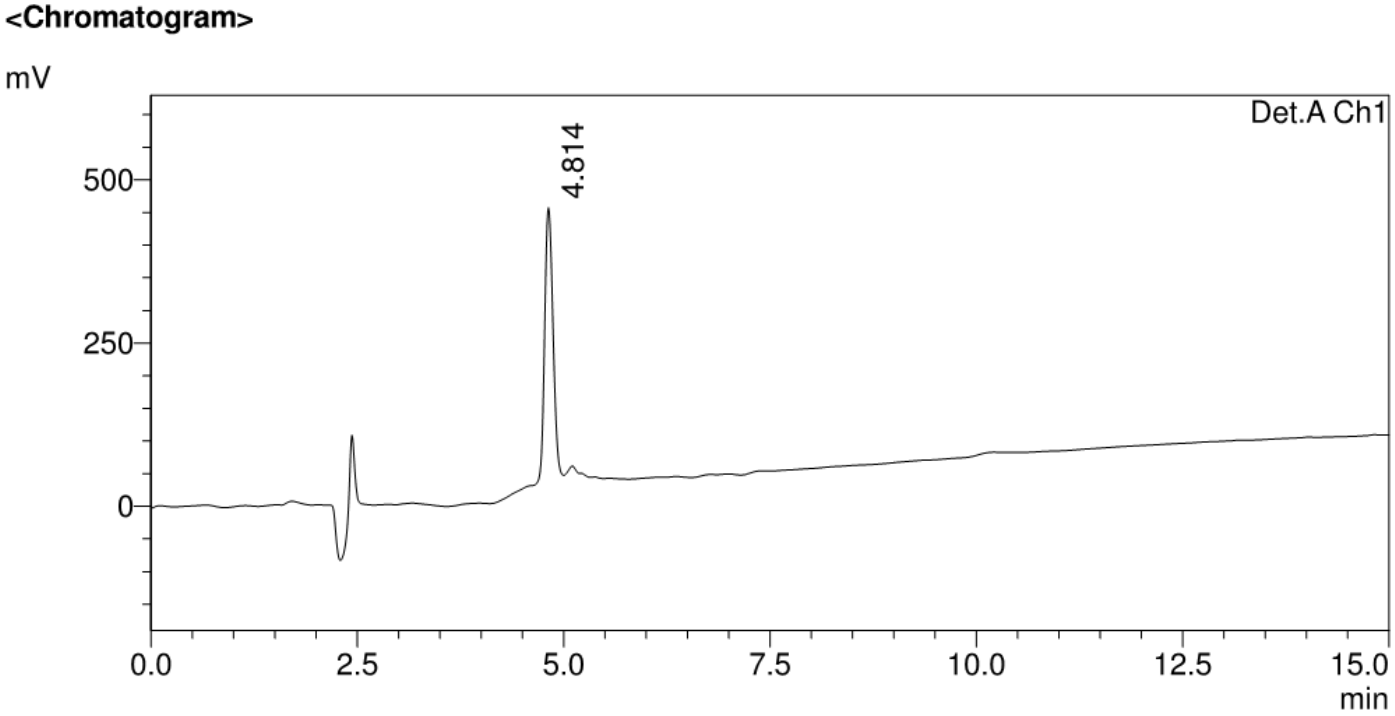

B)

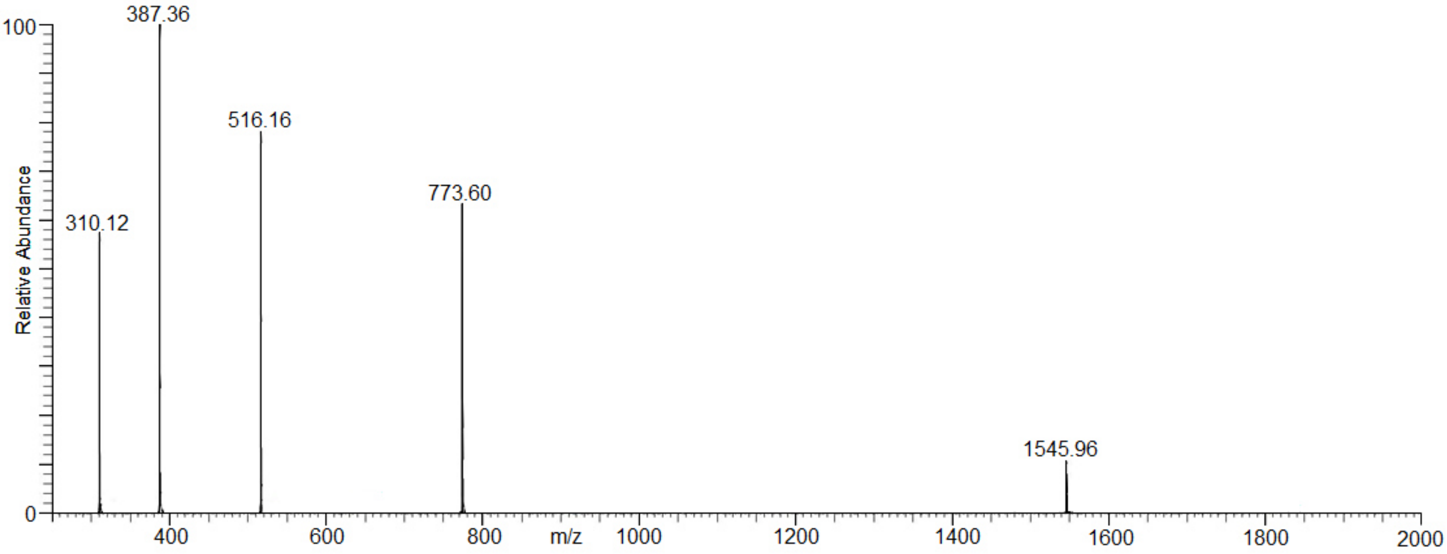

Supplementary Figure S2: A) Analytical HPLC profile and **B**) ESI-MS analysis of H3$_{1\text{-}15}$Orn4 peptide after prep-HPLC purification. It elutes at 4.8 min. Calculated mass is 1545.70 Da, found 1545.96 Da [M+H]$^{1+}$, 773.60 [M+2H]$^{2+}$, 516.16 [M+3H]$^{3+}$, 387.36 [M+4H]$^{4+}$, 310.12 [M+5H]$^{5+}$.

A)

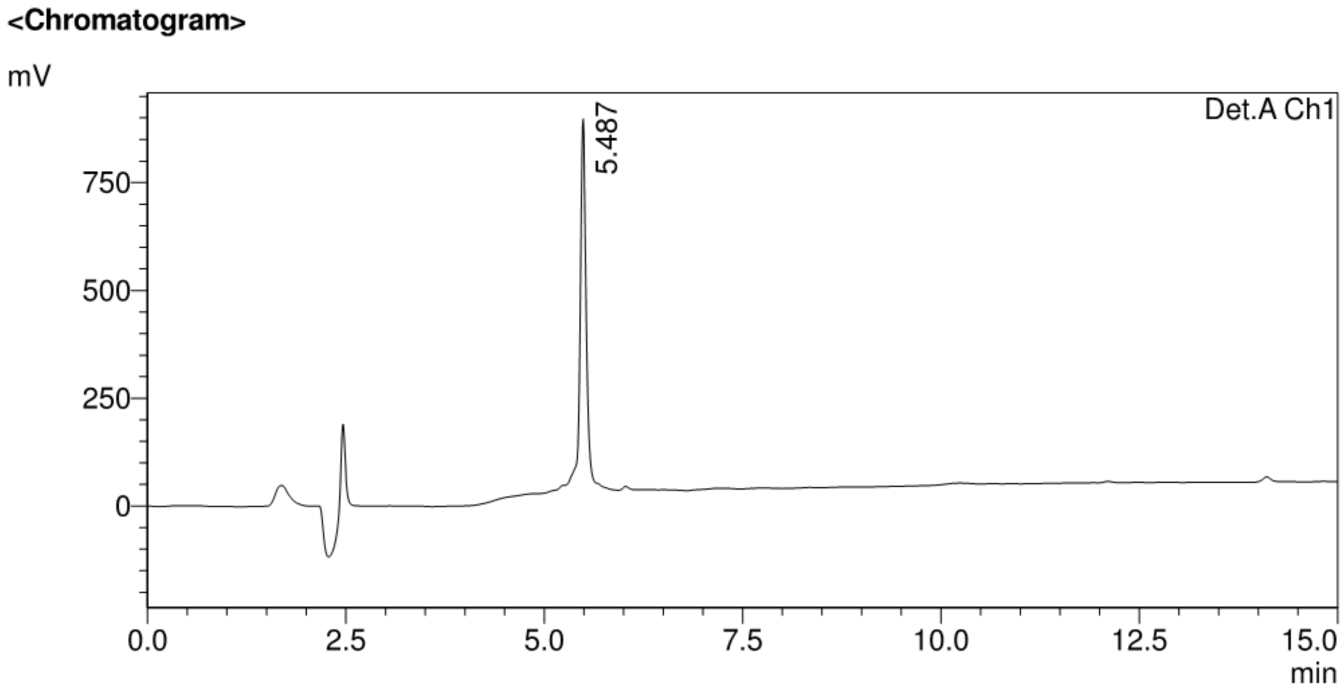

B)

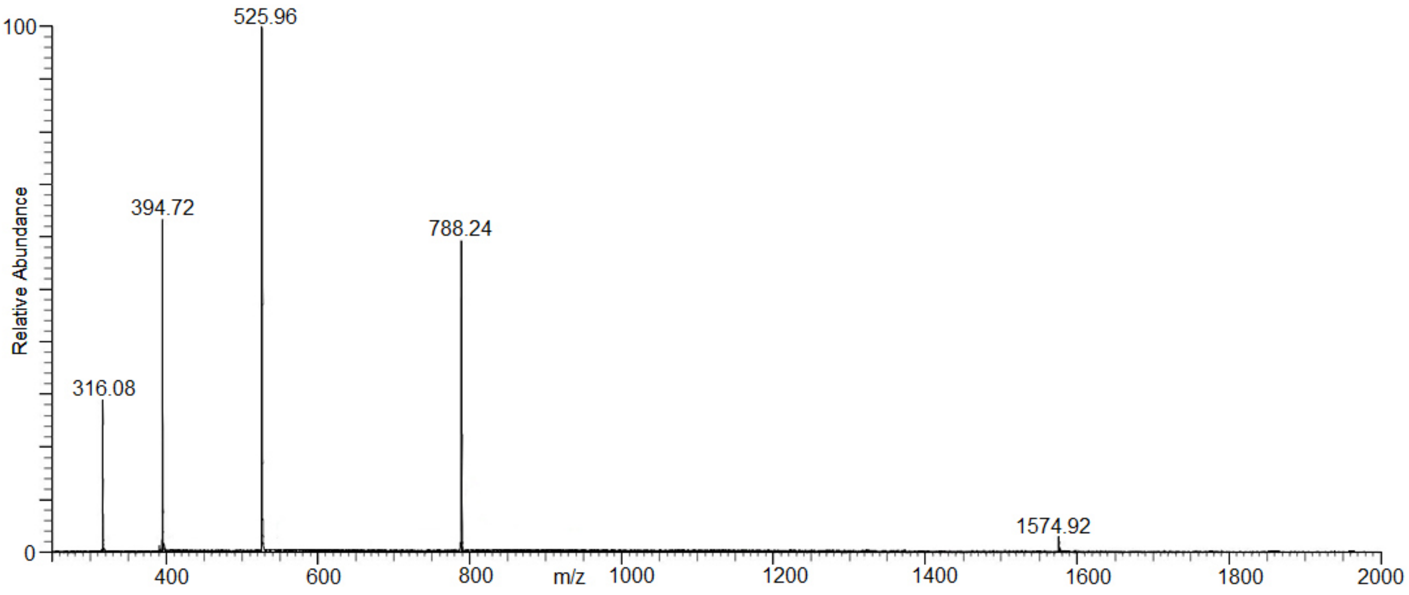

Supplementary Figure S3: A) Analytical HPLC profile and **B**) ESI-MS analysis of $H3_{1\text{-}15}hK4$ peptide after prep-HPLC purification. It elutes at 5.5 min. Calculated mass is 1573.80 Da, found 1574.92 Da $[M+H]^{1+}$, 788.24 $[M+2H]^{2+}$, 525.96 $[M+3H]^{3+}$, 394.72 $[M+4H]^{4+}$, 316.08 $[M+5H]^{5+}$.

A)

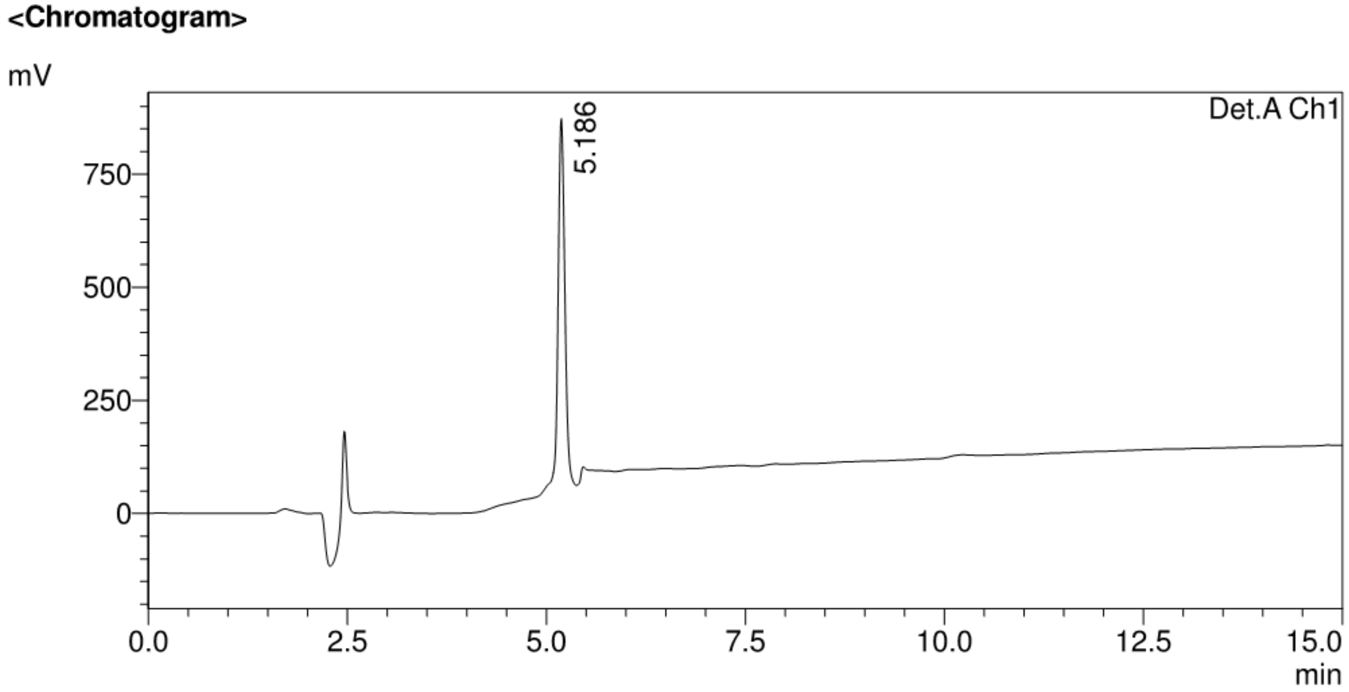

B)

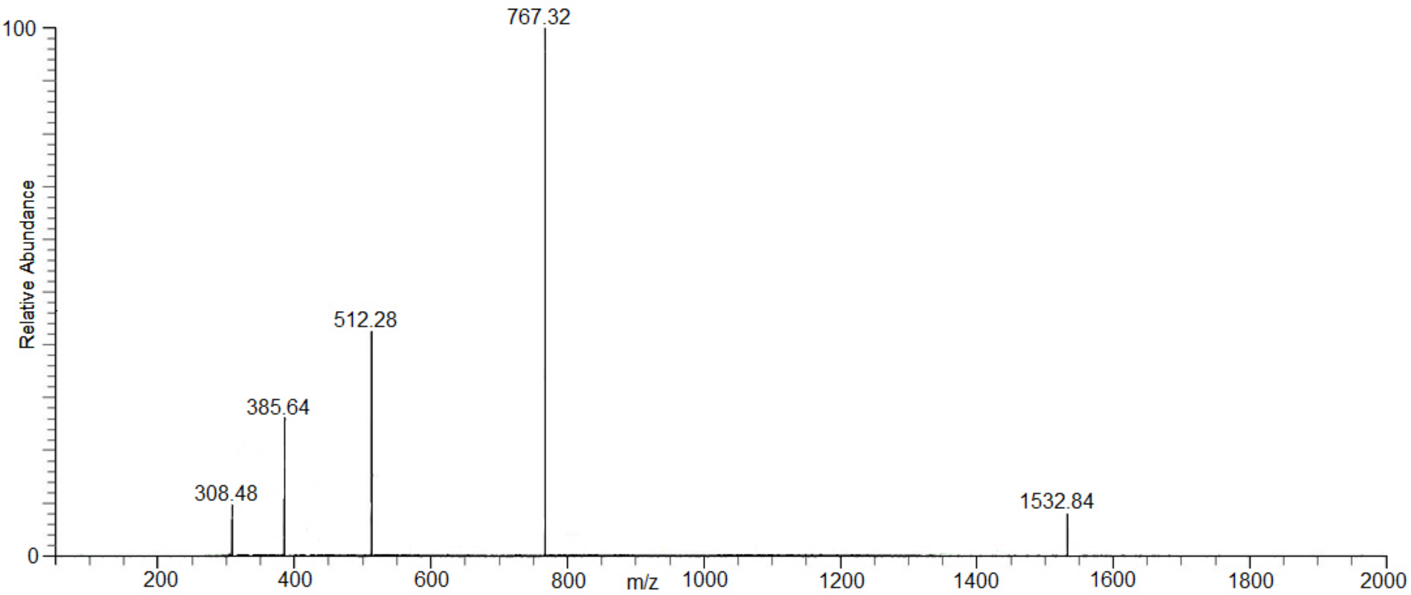

Supplementary Figure S4: A) Analytical HPLC profile and **B**) ESI-MS analysis of $H3_{1\text{-}15}Dab4$ peptide after prep-HPLC purification. It elutes at 5.2 min. Calculated mass is 1531.70 Da, found 1532.84 Da $[M+H]^{1+}$, 767.32 $[M+2H]^{2+}$, 512.28 $[M+3H]^{3+}$, 385.64 $[M+4H]^{4+}$, 308.48 $[M+5H]^{5+}$.

A)

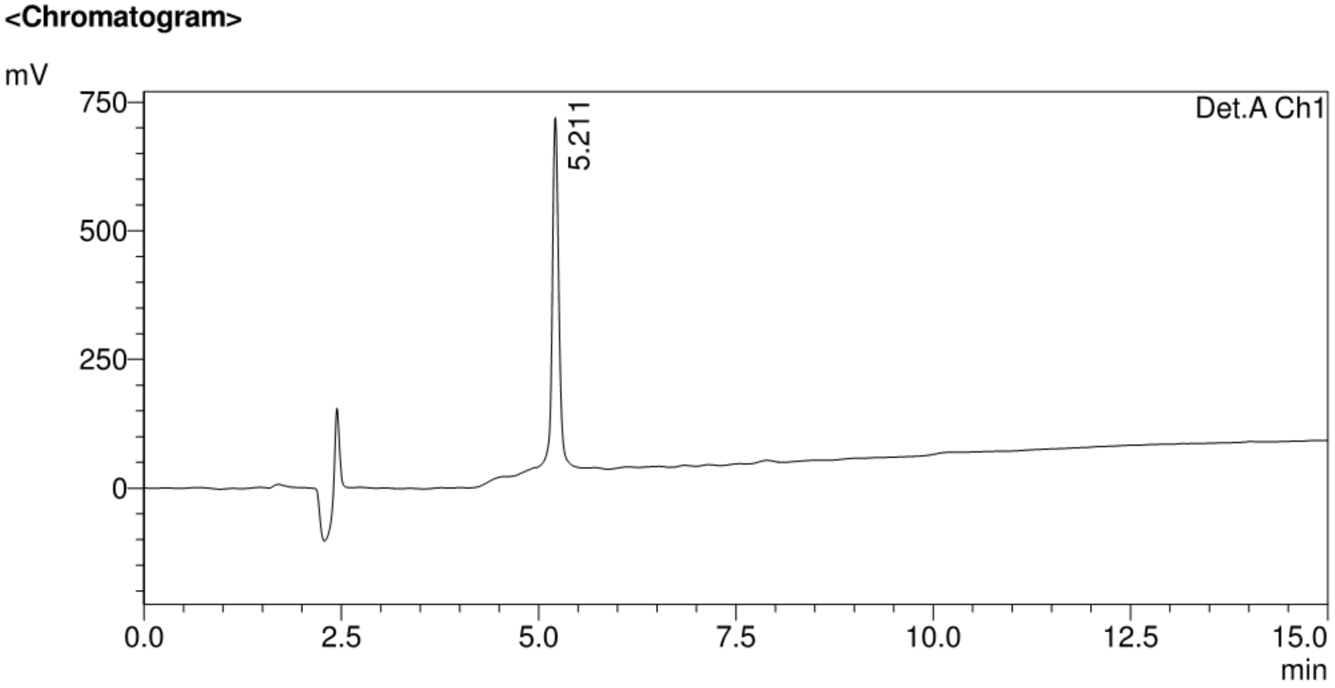

B)

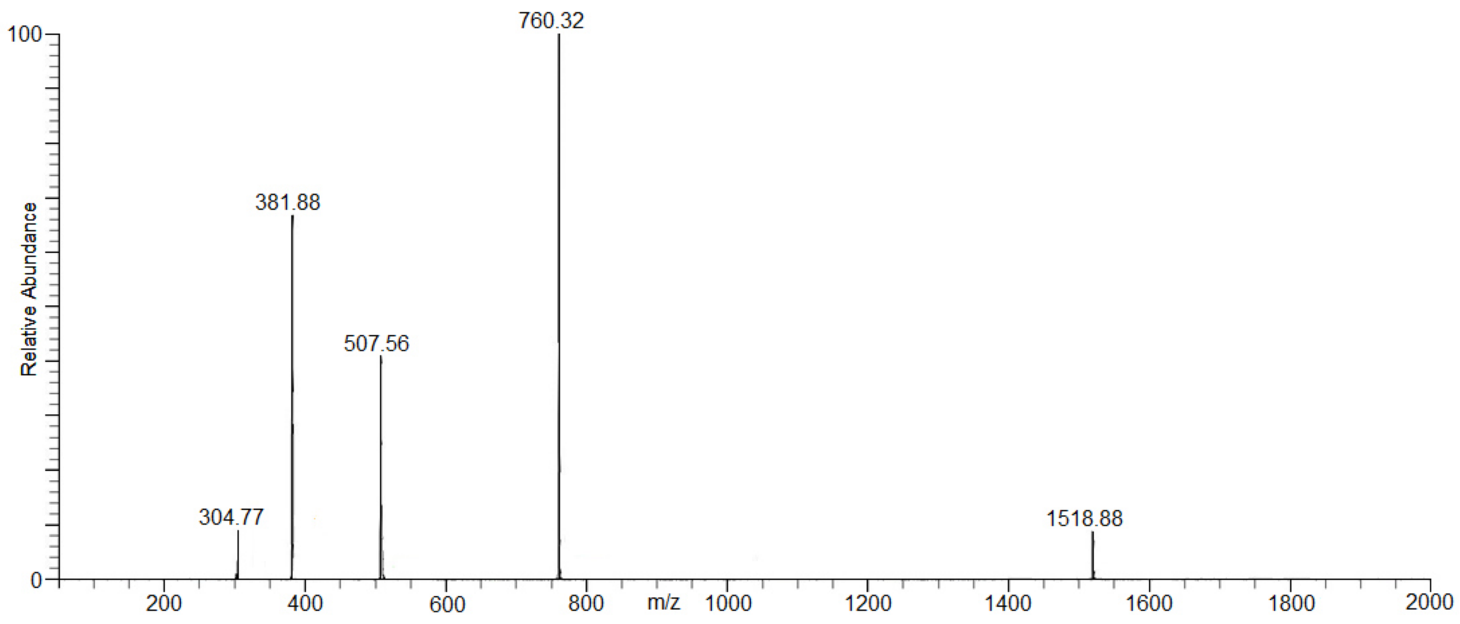

Supplementary Figure S5: A) Analytical HPLC profile and **B**) ESI-MS analysis of H3$_{1\text{-}15}$Dap4 peptide after prep-HPLC purification. It elutes at 5.2 min. Calculated mass is 1517.70 Da, found 1518.88 Da $[M+H]^{1+}$, 760.32 $[M+2H]^{2+}$, 507.56 $[M+3H]^{3+}$, 381.88 $[M+4H]^{4+}$, 304.77 $[M+5H]^{5+}$.

A)

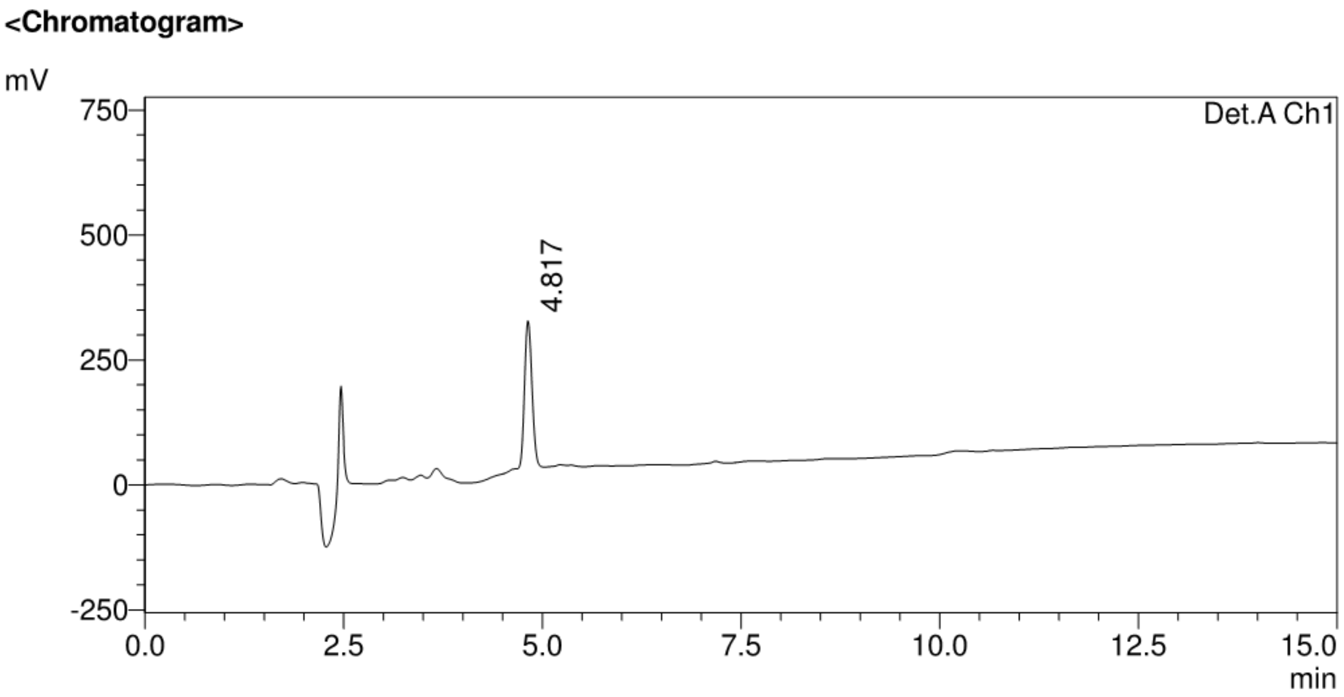

B)

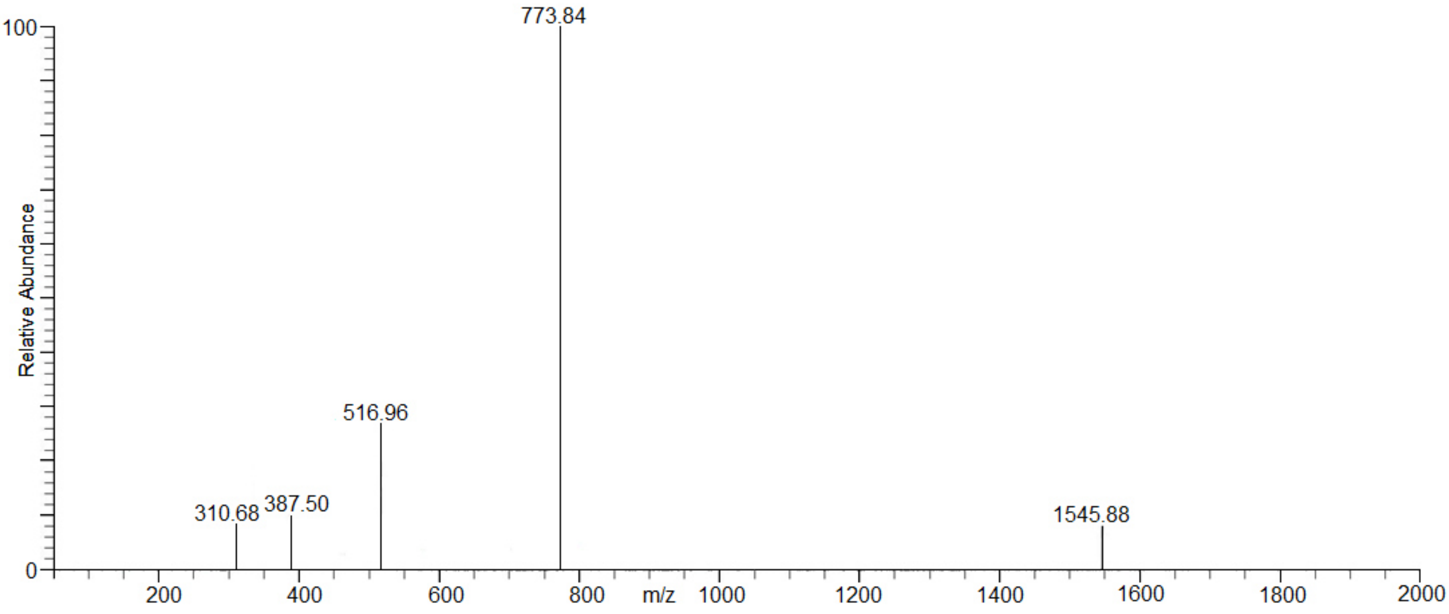

Supplementary Figure S6: A) Analytical HPLC profile and **B**) ESI-MS analysis of H3$_{1\text{-}15}$Orn9 peptide after prep-HPLC purification. It elutes at 4.8 min. Calculated mass is 1545.70 Da, found 1545.88 Da $[M+H]^{1+}$, 773.84 $[M+2H]^{2+}$, 516.96 $[M+3H]^{3+}$, 387.50 $[M+4H]^{4+}$, 310.68 $[M+5H]^{5+}$.

A)

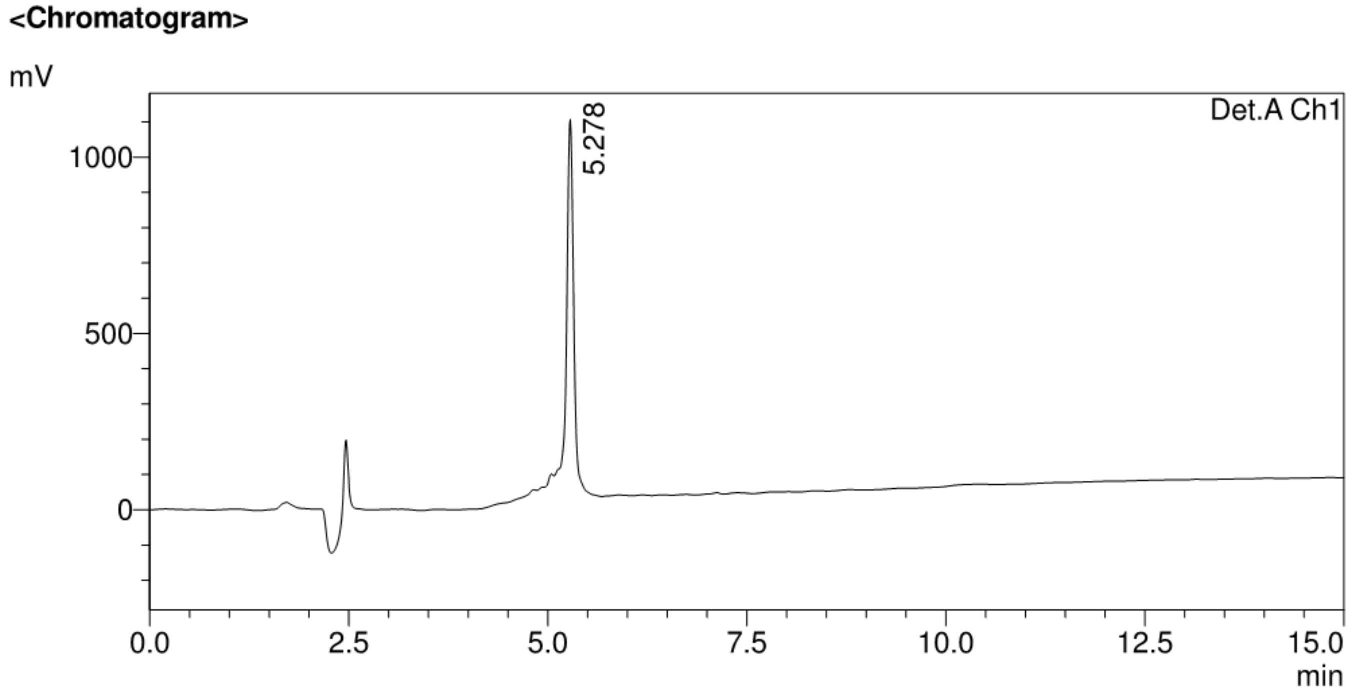

B)

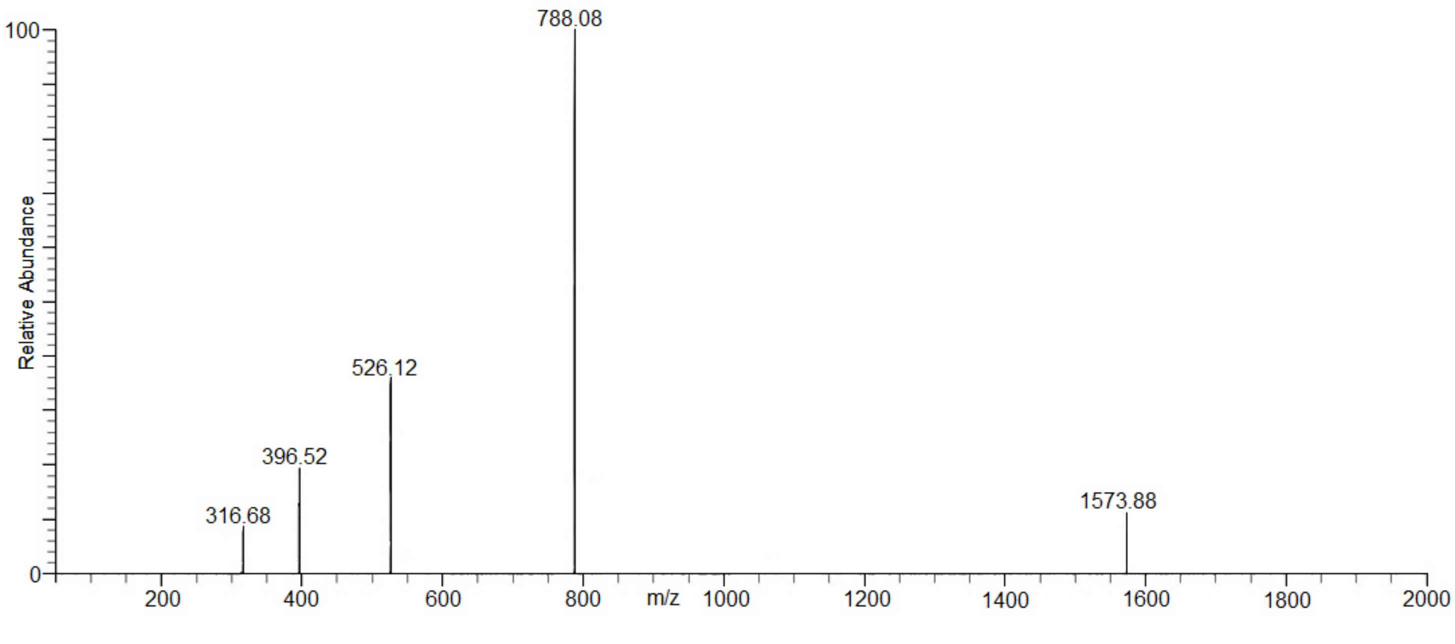

Supplementary Figure S7: A) Analytical HPLC profile and **B**) ESI-MS analysis of H3$_{1\text{-}15}$hK9 peptide after prep-HPLC purification. It elutes at 5.3 min. Calculated mass is 1573.80 Da, found 1573.88 Da $[M+H]^{1+}$, 788.08 $[M+2H]^{2+}$, 526.12 $[M+3H]^{3+}$, 396.52 $[M+4H]^{4+}$, 316.68 $[M+5H]^{5+}$.

A)

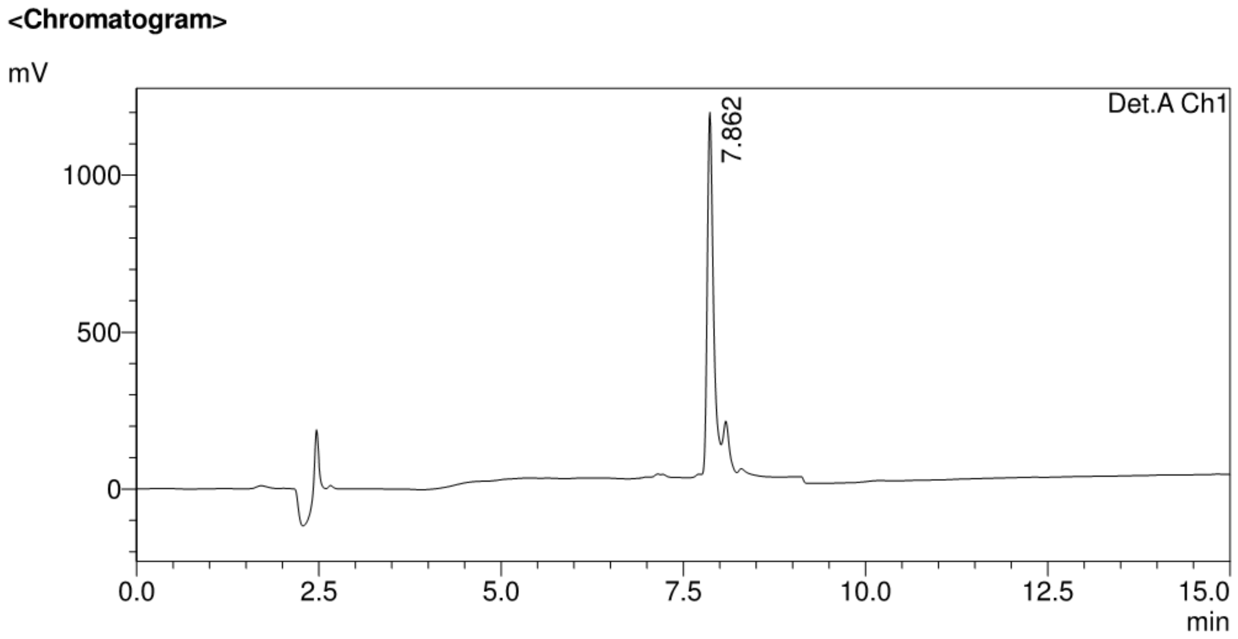

B)

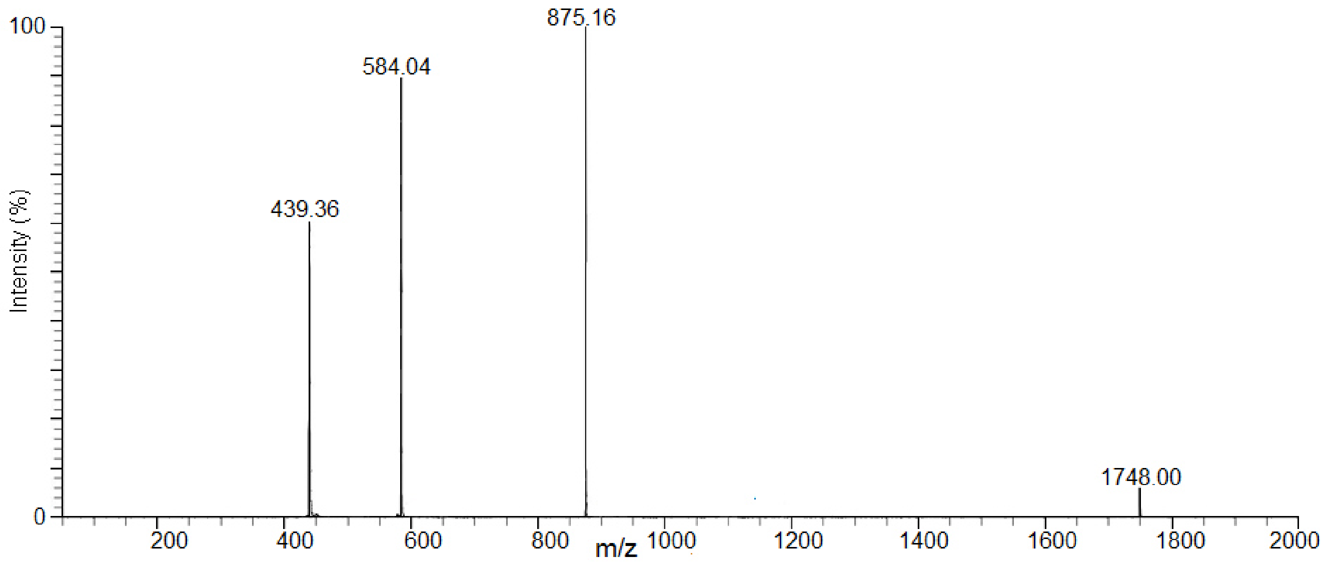

Supplementary Figure S8: A) Analytical HPLC profile and **B**) ESI-MS analysis of H4$_{13\text{-}27}$K20 peptide after prep-HPLC purification. It elutes at 7.8 min. Calculated mass is 1748.00 Da, found 1748.00 Da $[M+H]^{1+}$, 875.16 $[M+2H]^{2+}$, 584.04 $[M+3H]^{3+}$, 439.36 $[M+4H]^{4+}$.

A)

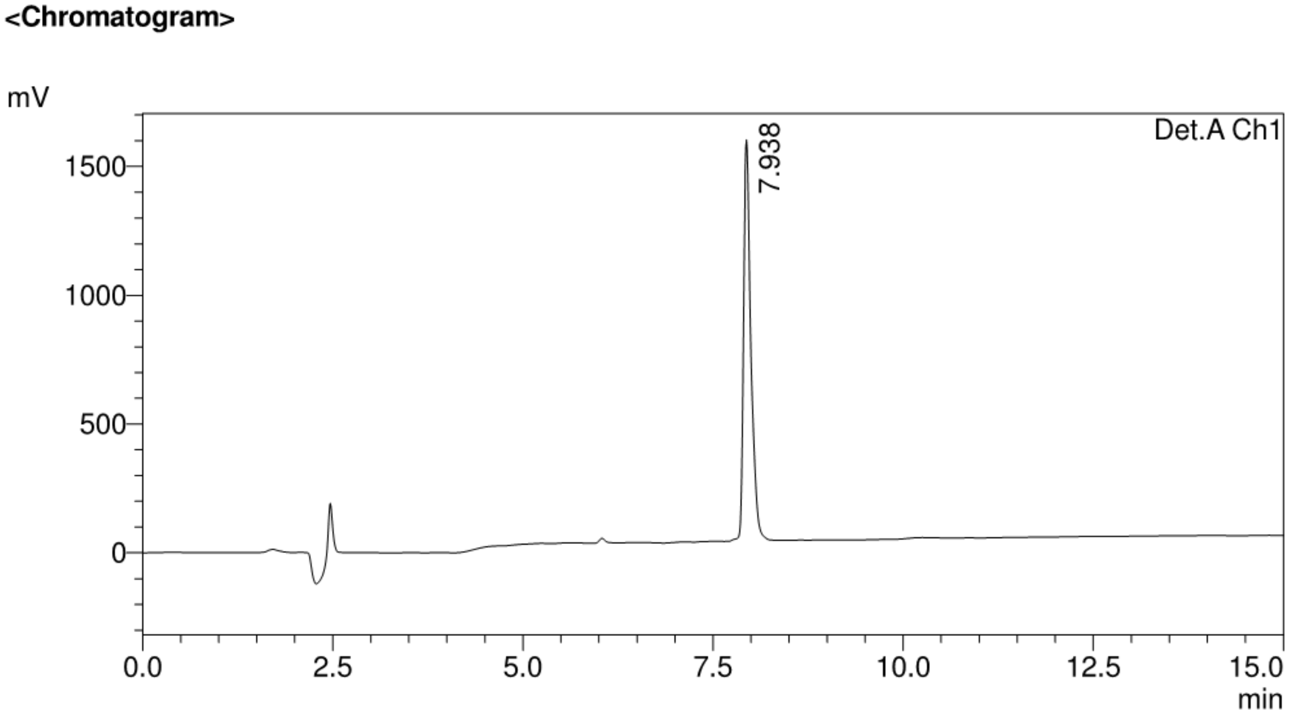

B)

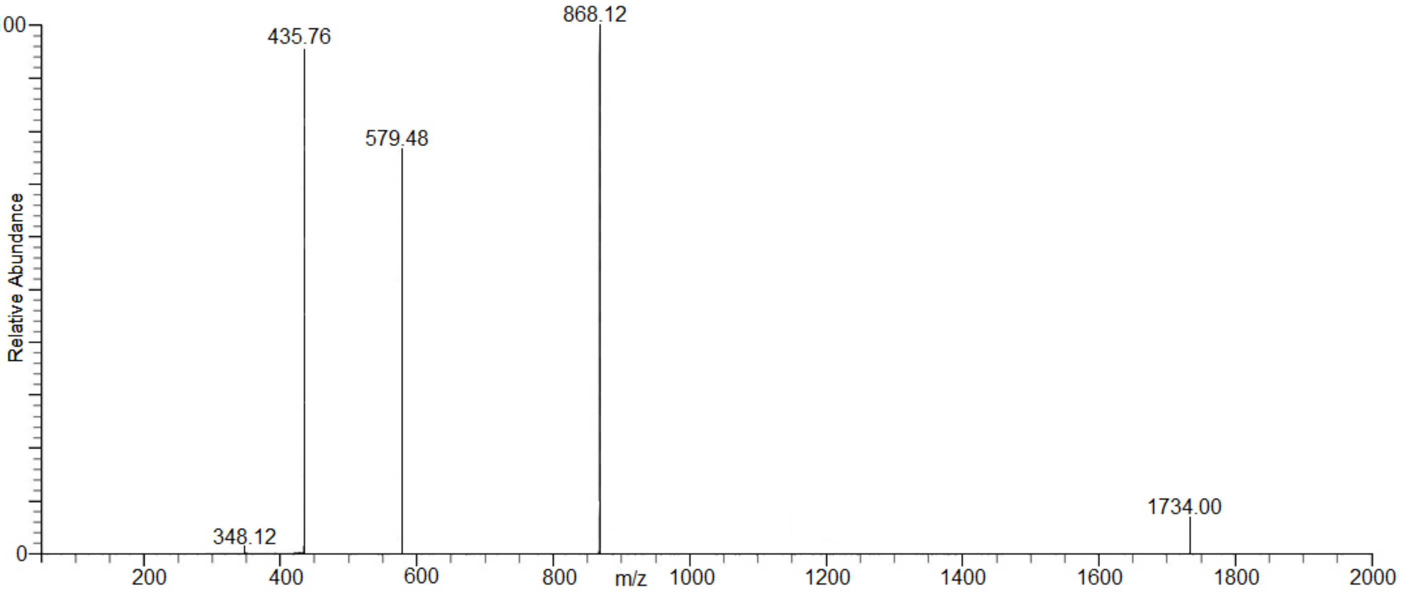

Supplementary Figure S9: A) Analytical HPLC profile and **B**) ESI-MS analysis of $H4_{13\text{-}27}$Orn20 peptide after prep-HPLC purification. It elutes at 7.9 min. Calculated mass is 1734.00 Da, found 1734.00 Da $[M+H]^{1+}$, 868.12 $[M+2H]^{2+}$, 579.48 $[M+3H]^{3+}$, 435.76 $[M+4H]^{4+}$, 348.12 $[M+5H]^{5+}$.

A)

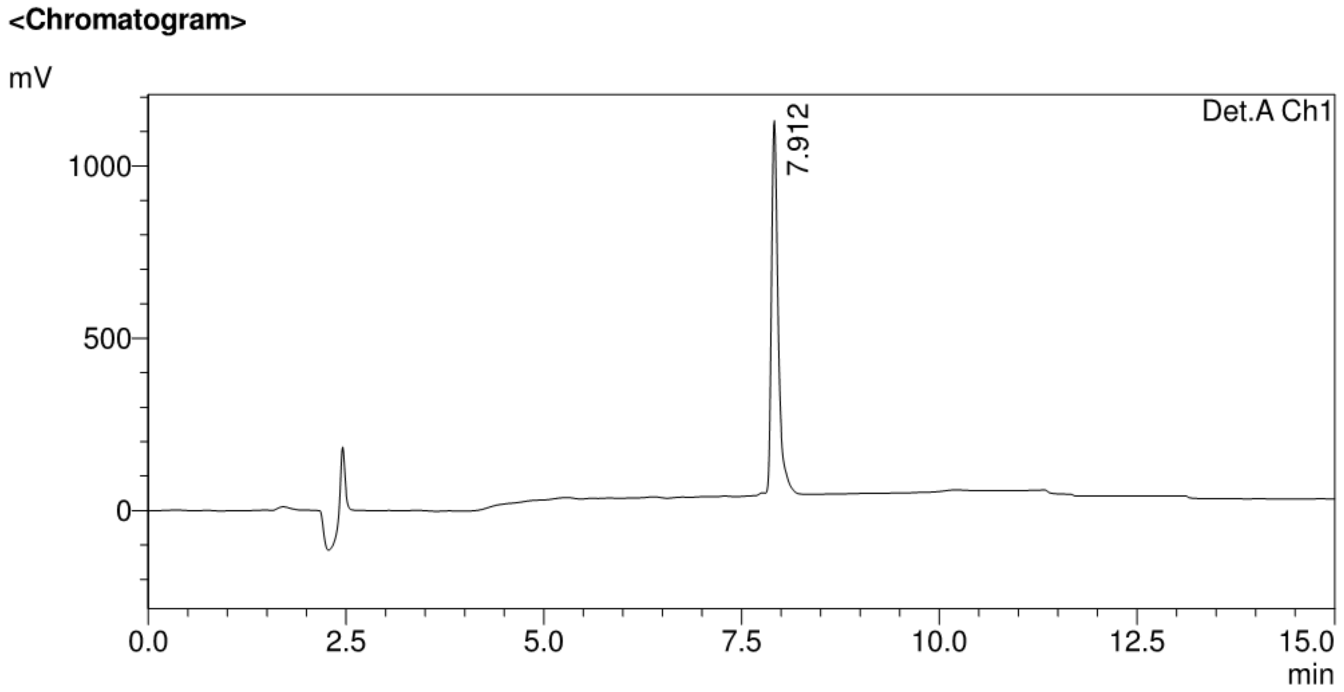

B)

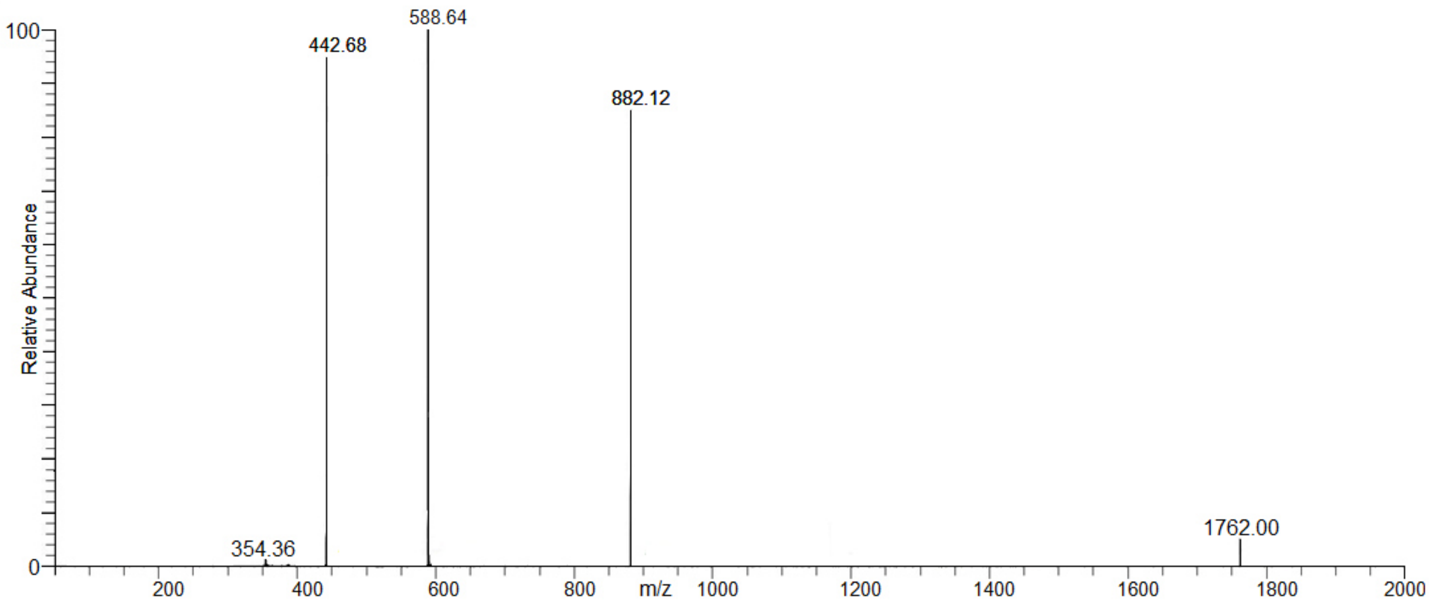

Supplementary Figure S10: A) Analytical HPLC profile and **B**) ESI-MS analysis of $H4_{13\text{-}27}$hK20 peptide after prep-HPLC purification. It elutes at 7.9 min. Calculated mass is 1762.00 Da, found 1762.00 Da $[M+H]^{1+}$, 882.12 $[M+2H]^{2+}$, 588.64 $[M+3H]^{3+}$, 442.68 $[M+4H]^{4+}$, 354.36 $[M+5H]^{5+}$.

A)

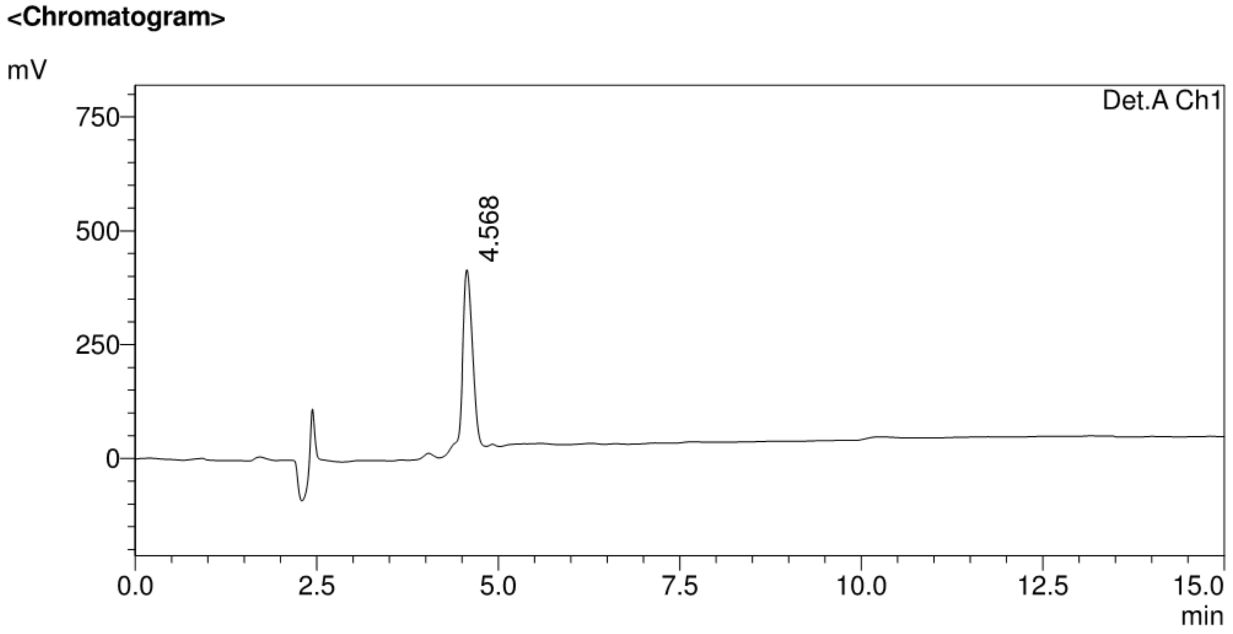

B)

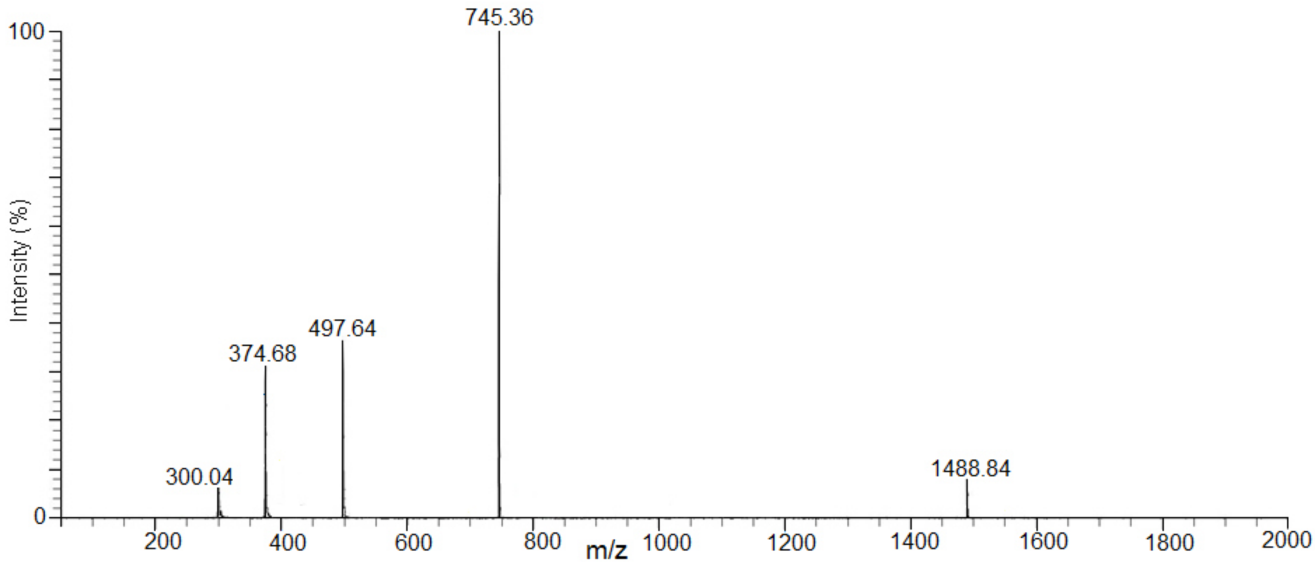

Supplementary Figure S11: (**A**) Analytical HPLC profile and (**B**) ESI-MS analysis of H3 (1-14) peptide after prep-HPLC purification. It elutes at 4.5 min. Calculated mass is 1488.70 Da, found 1488.84 Da $[M+H]^{1+}$, 745.36 $[M+2H]^{2+}$, 497.64 $[M+3H]^{3+}$, 374.68$[M+4H]^{4+}$, 300.04 $[M+5H]^{5+}$.

A)

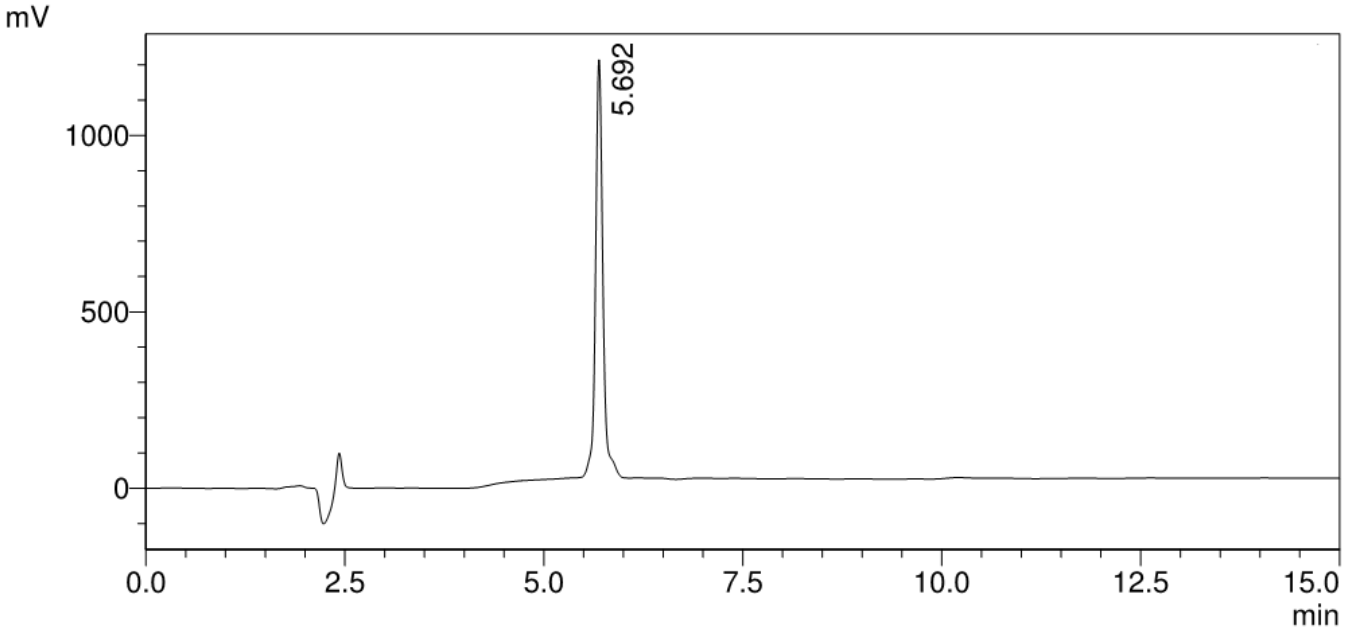

B)

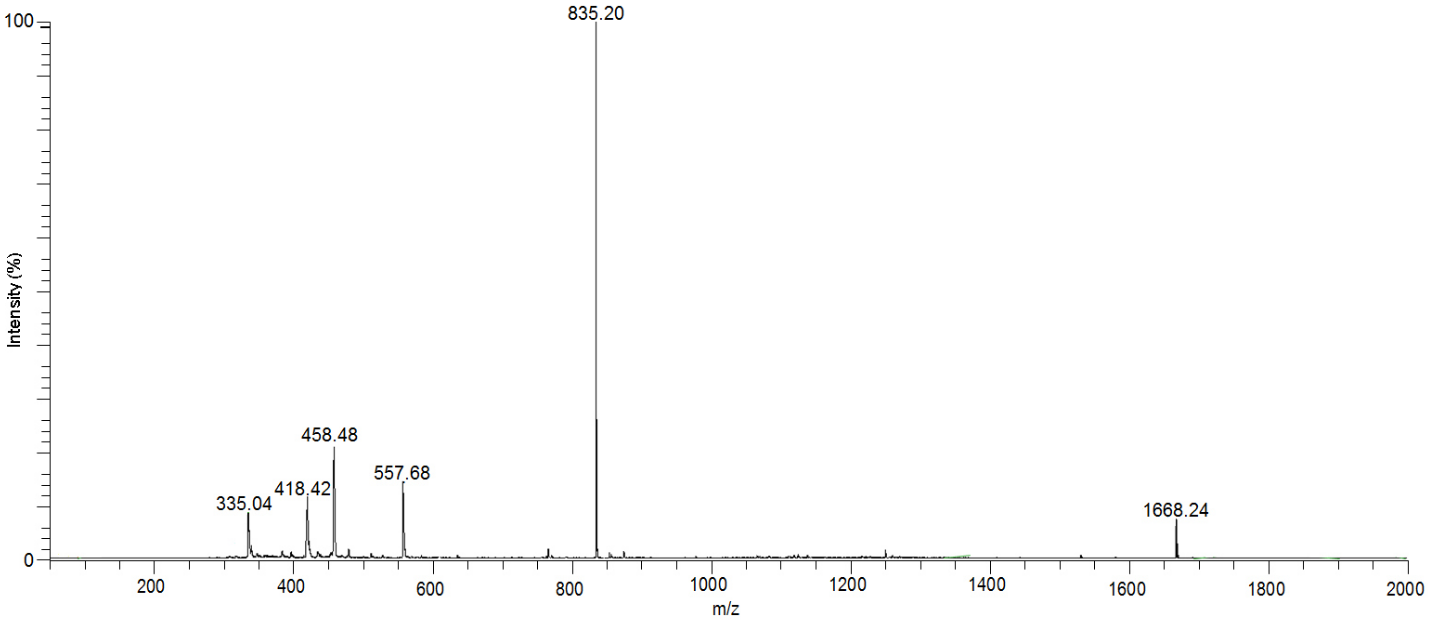

Supplementary Figure S12: A) Analytical HPLC profile and **B**) ESI-MS analysis of p53K373 peptide after prep-HPLC purification. It elutes at 5.7 min. Calculated mass is 1668.83 Da, found 1668.24 Da $[M+H]^{1+}$, 835.20 $[M+2H]^{2+}$, 557.68 $[M+3H]^{3+}$, 418.42 $[M+4H]^{4+}$, 335.04 $[M+5H]^{5+}$.

A)

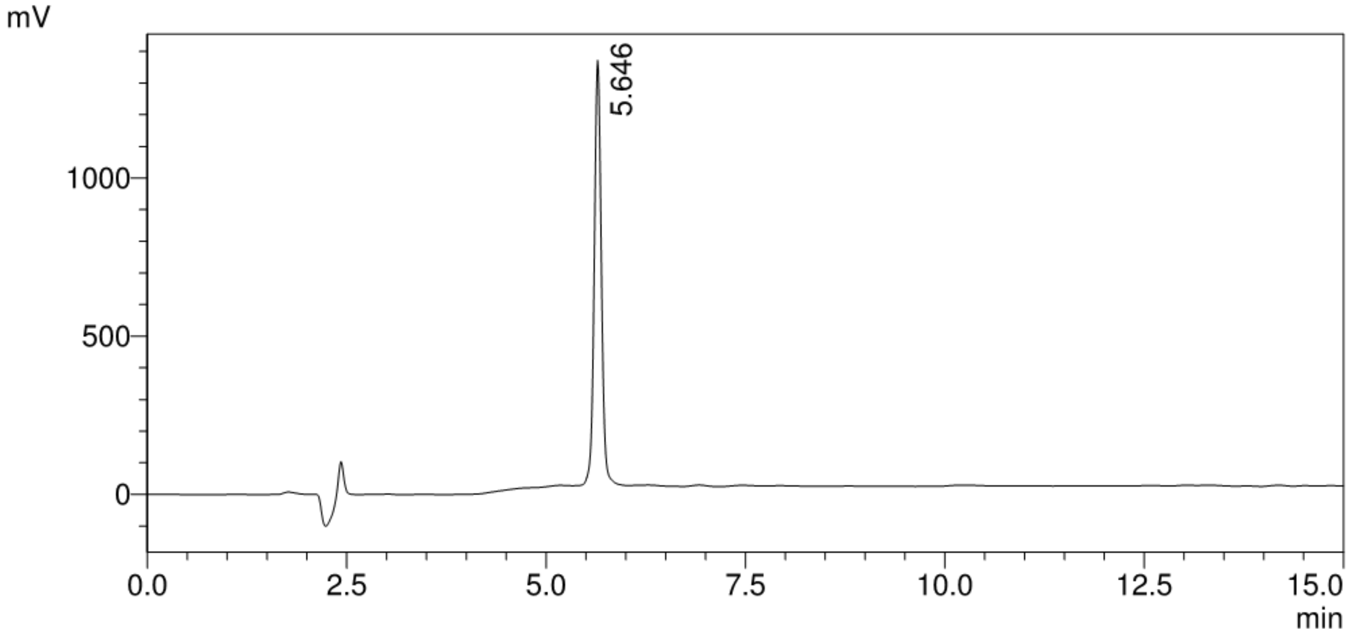

B)

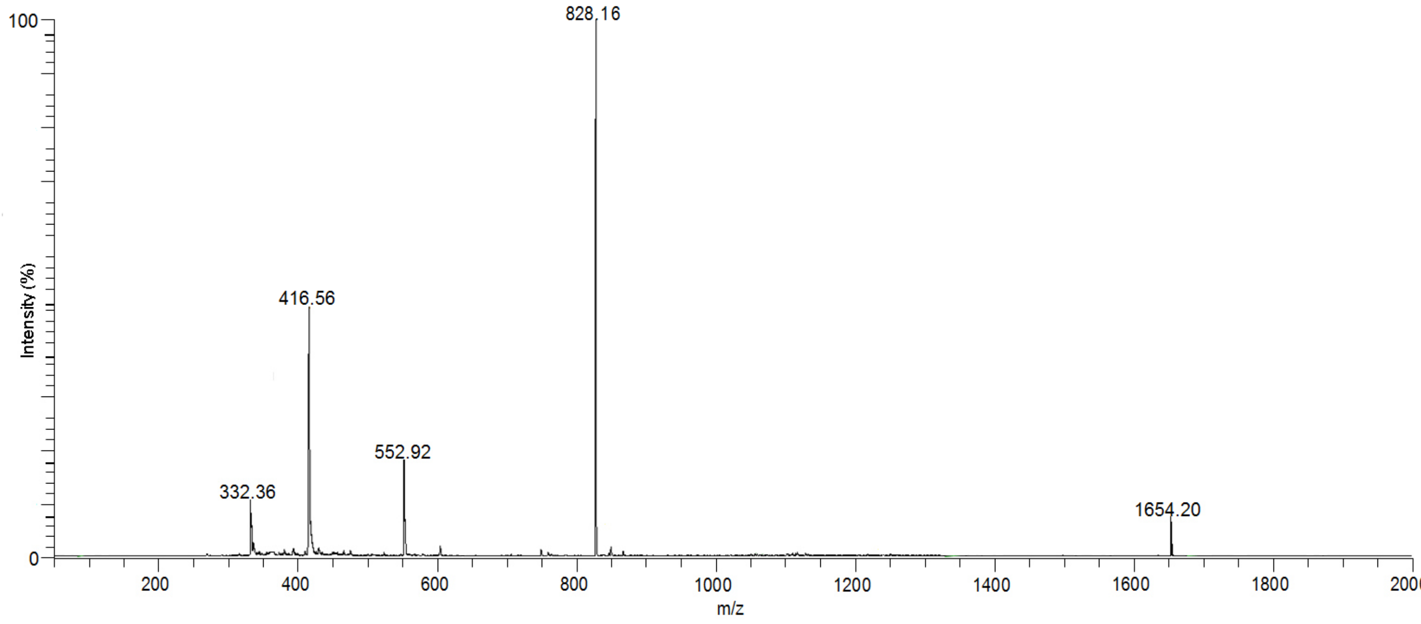

Supplementary Figure S13: A) Analytical HPLC profile and **B**) ESI-MS analysis of p53Orn373 peptide after prep-HPLC purification. It elutes at 5.6 min. Calculated mass is 1654.83 Da, found 1654.20 Da $[M+H]^{1+}$, 828.16 $[M+2H]^{2+}$, 552.92 $[M+3H]^{3+}$, 416.56 $[M+4H]^{4+}$, 332.36 $[M+5H]^{5+}$.

A)

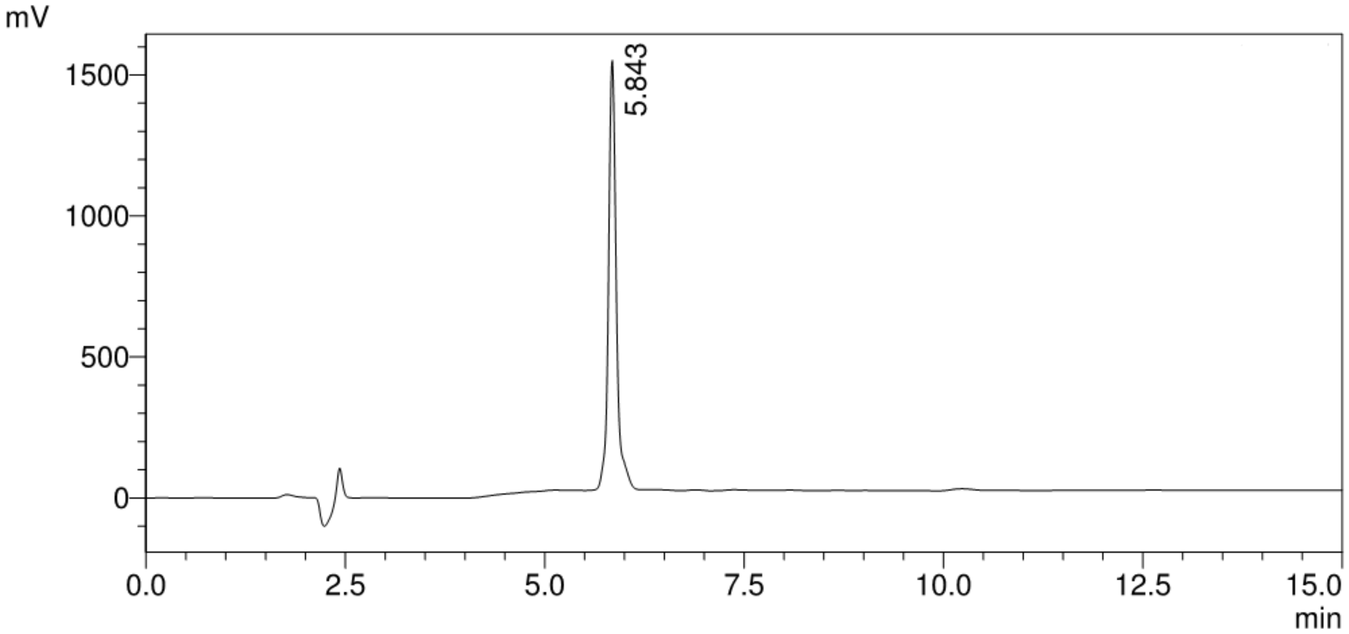

B)

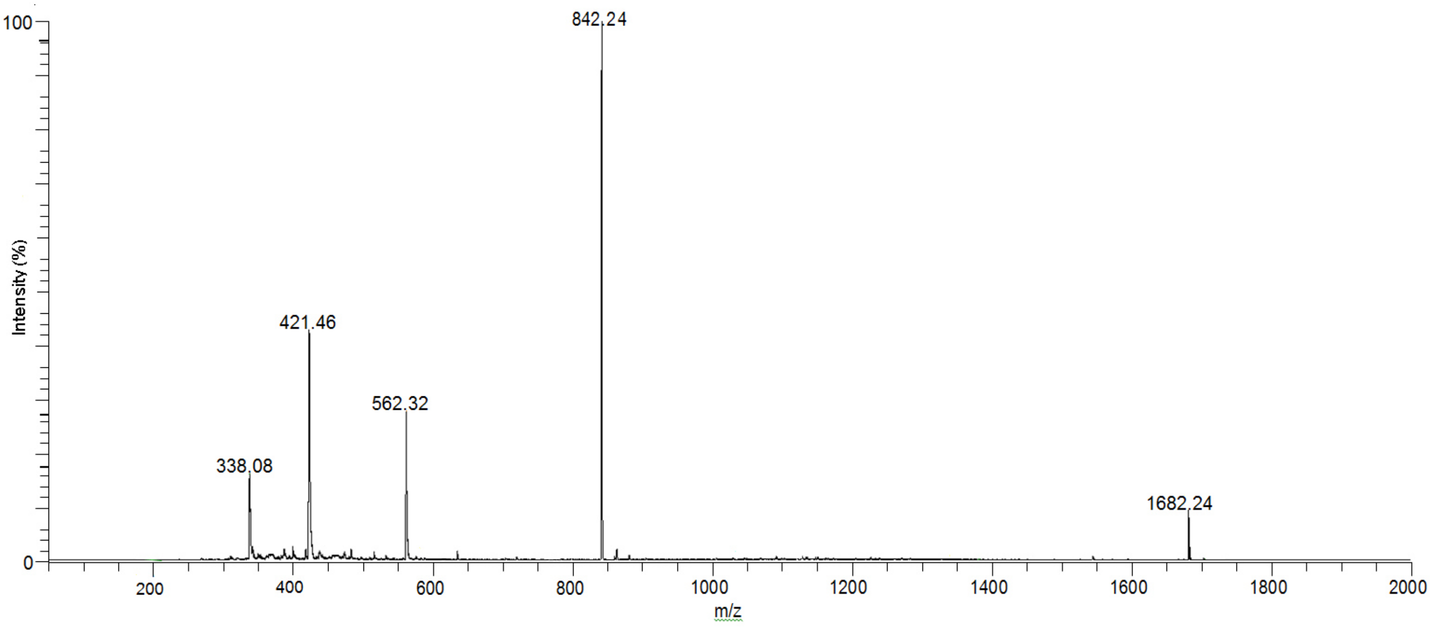

Supplementary Figure S14: A) Analytical HPLC profile and **B**) ESI-MS analysis of p53hK373 peptide after prep-HPLC purification. It elutes at 5.8 min. Calculated mass is 1682.83 Da, found 1682.24 Da $[M+H]^{1+}$, 842.24 $[M+2H]^{2+}$, 562.32 $[M+3H]^{3+}$, 421.46 $[M+4H]^{4+}$, 338.08 $[M+5H]^{5+}$.

A)

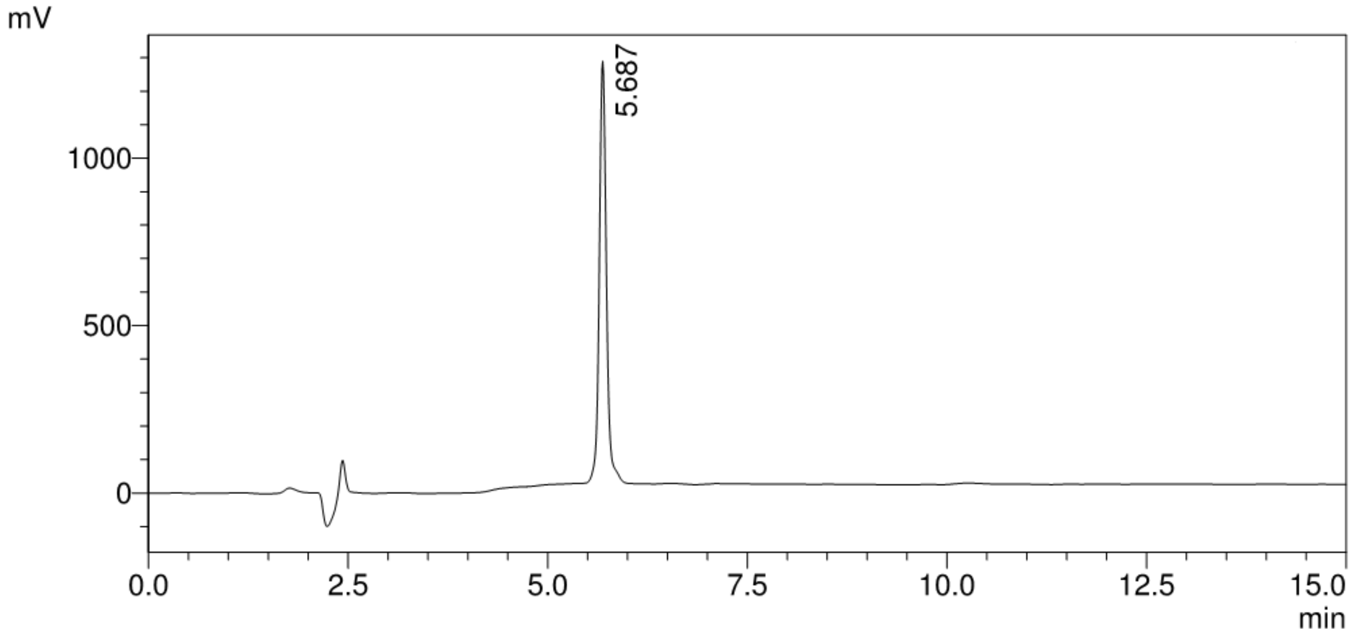

B)

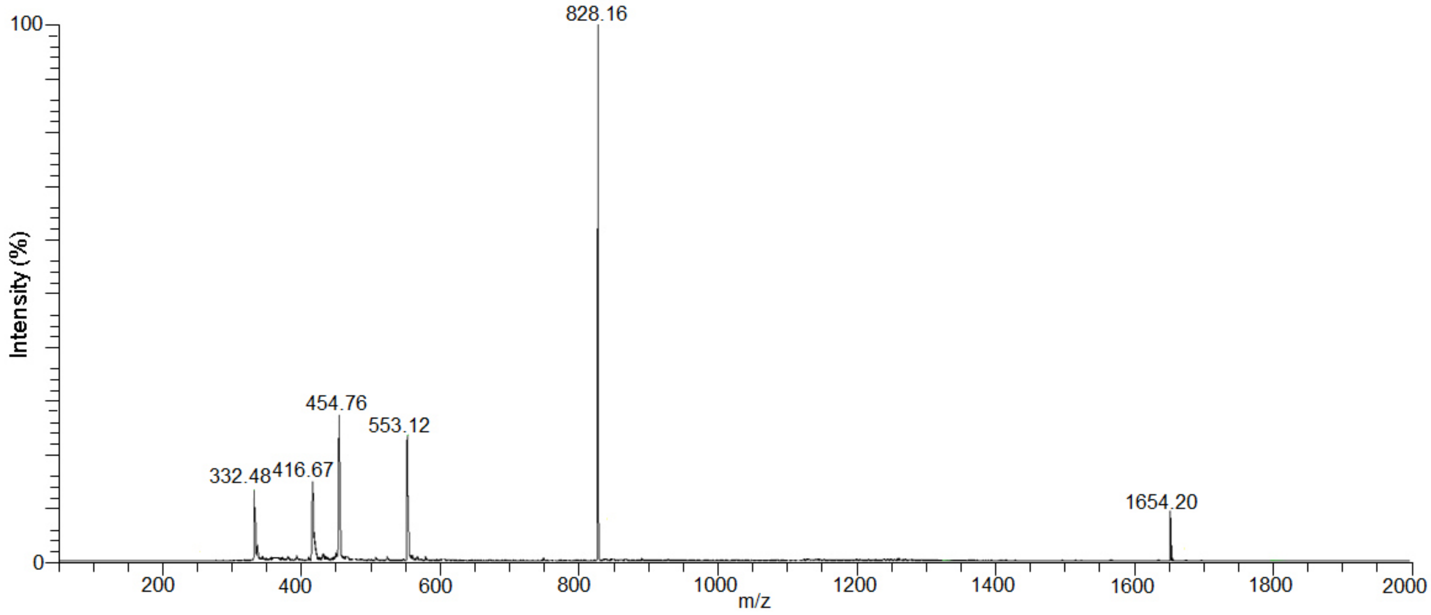

Supplementary Figure S15: A) Analytical HPLC profile and **B**) ESI-MS analysis of p53Orn372 peptide after prep-HPLC purification. It elutes at 5.7 min. Calculated mass is 1654.83 Da, found 1654.20 Da $[M+H]^{1+}$, 828.16 $[M+2H]^{2+}$, 553.12 $[M+3H]^{3+}$, 416.67 $[M+4H]^{4+}$, 332.48 $[M+5H]^{5+}$.

A)

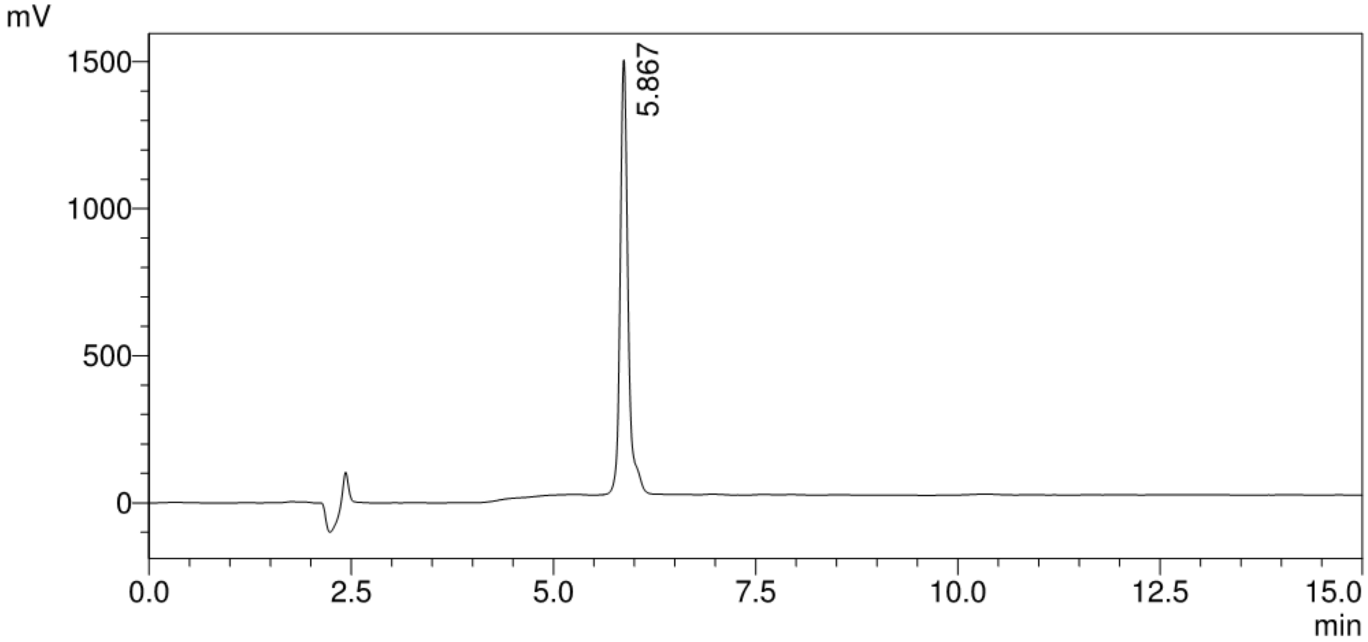

B)

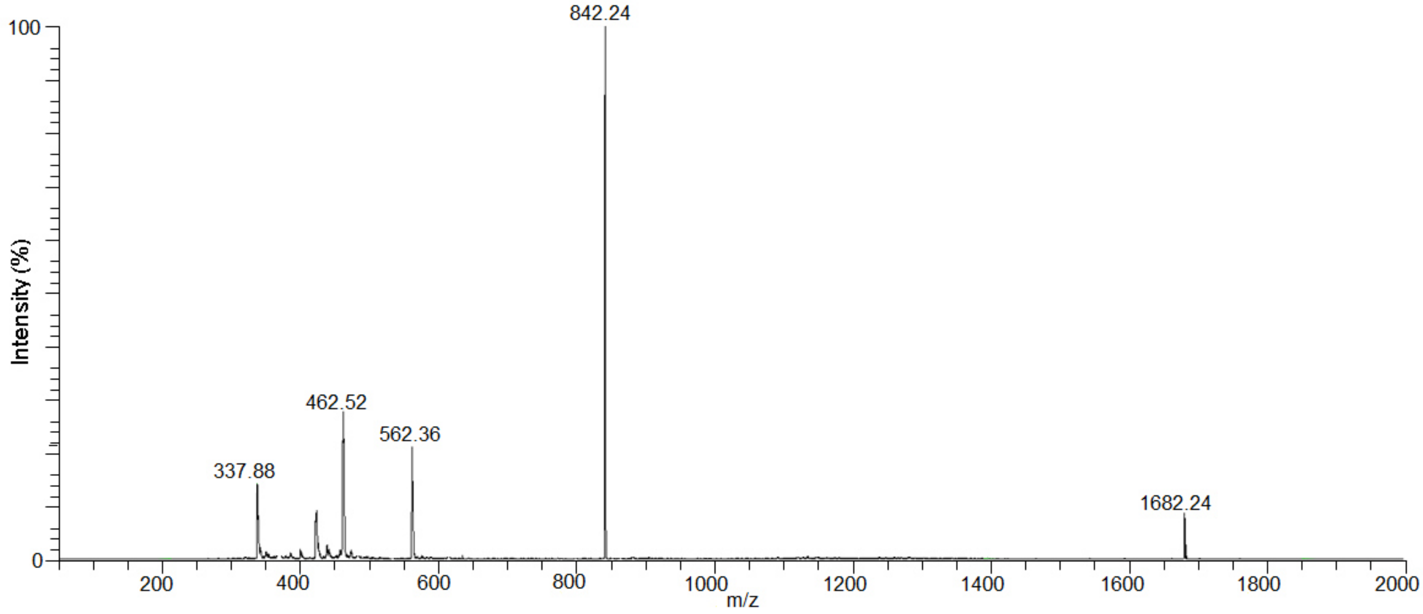

Supplementary Figure S16: A) Analytical HPLC profile and **B**) ESI-MS analysis of p53hK372 peptide after prep-HPLC purification. It elutes at 5.8 min. Calculated mass is 1682.83 Da, found 1682.24 Da $[M+H]^{1+}$, 842.24 $[M+2H]^{2+}$, 562.36 $[M+3H]^{3+}$, 421.46 $[M+4H]^{4+}$, 337.88 $[M+5H]^{5+}$.

3. MALDI supplementary figures

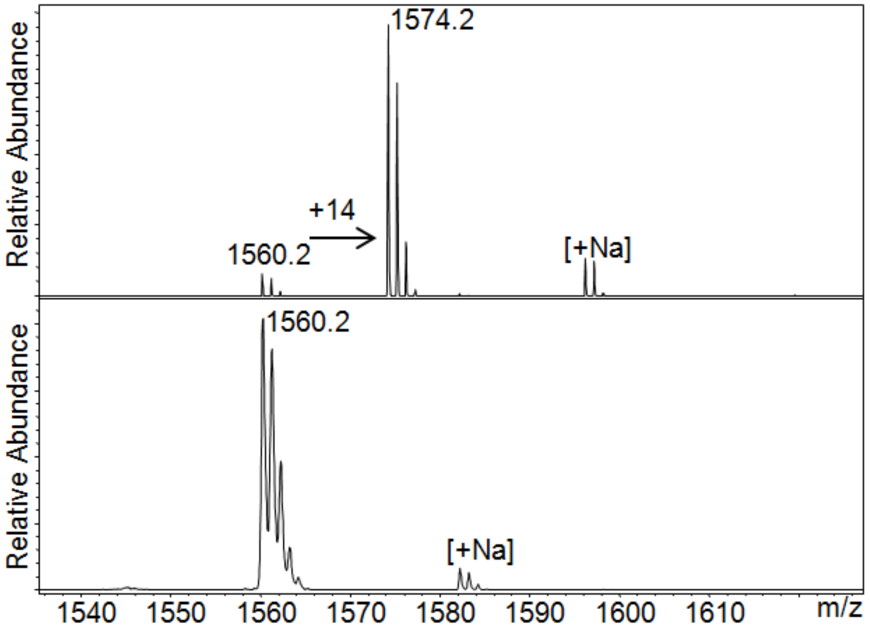

Supplementary Figure S17: MALDI-TOF mass spectra showing SETD7 (2 µM) catalyzed monomethylation of $H3_{1\text{-}15}K4$ peptide (100 µM) in the presence of SAM (200 µM), after 1 h at 37 °C (top panel). A control reaction in the absence of SETD7 (bottom panel).

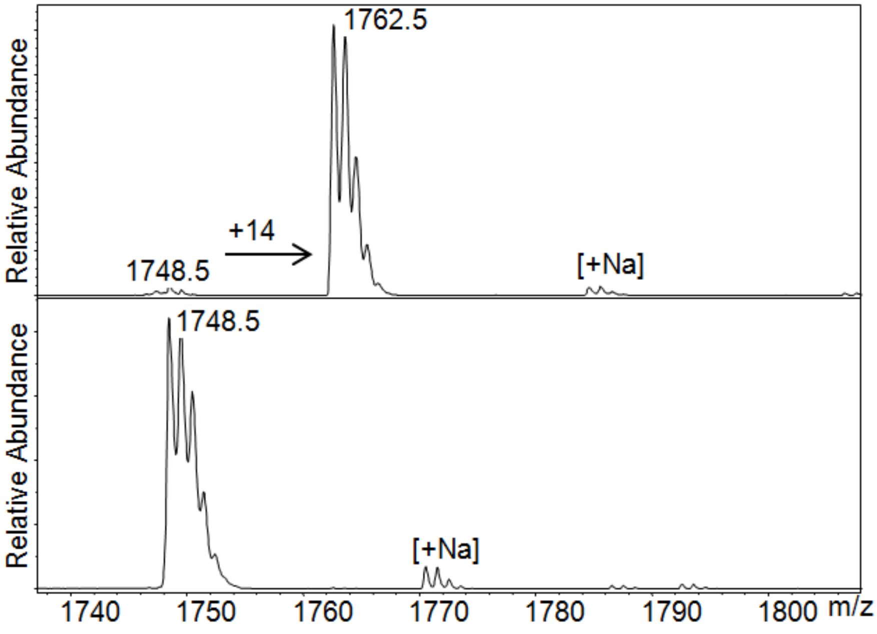

Supplementary Figure S18: MALDI-TOF mass spectra showing SETD8 (2 µM) catalyzed monomethylation of $H4_{13\text{-}27}K20$ peptide (100 µM) in the presence of SAM (200 µM), after 1 h at 37 °C (top panel). A control reaction in the absence of SETD8 (bottom panel).

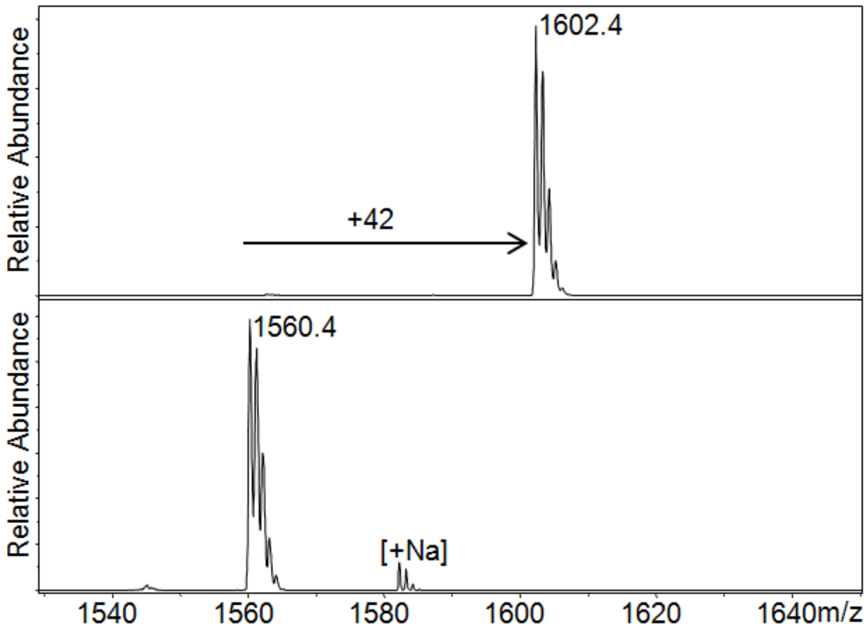

Supplementary Figure S19: MALDI-TOF mass spectra showing GLP (2 µM) catalyzed trimethylation of $H3_{1-15}K9$ peptide (100 µM) in the presence of SAM (500 µM), after 1 h at 37 °C (top panel). A control reaction in the absence of GLP (bottom panel).

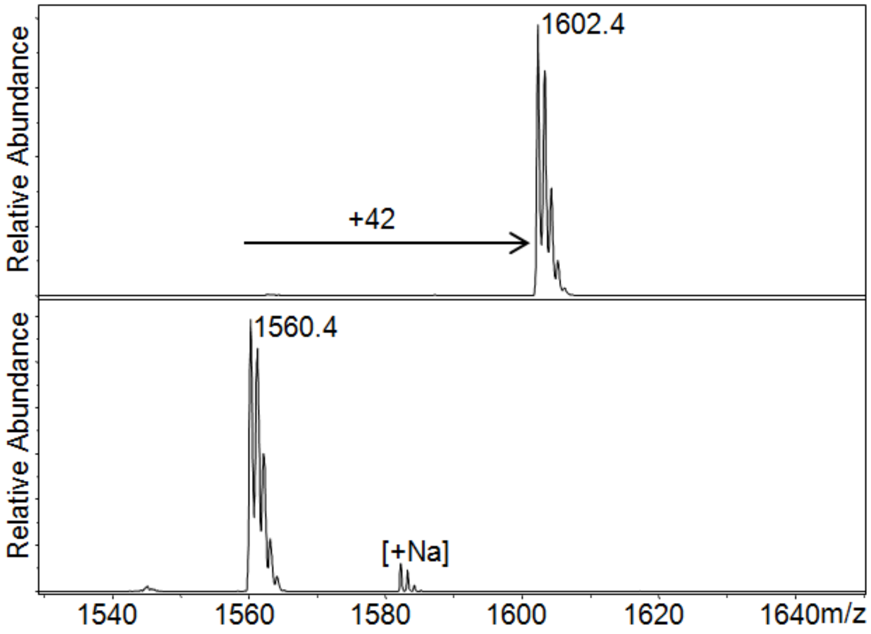

Supplementary Figure S20: MALDI-TOF mass spectra showing G9a (2 µM) catalyzed trimethylation of $H3_{1-15}K9$ peptide (100 µM) in the presence of SAM (500 µM), after 1 h at 37 °C (top panel). A control reaction in the absence of G9a (bottom panel).

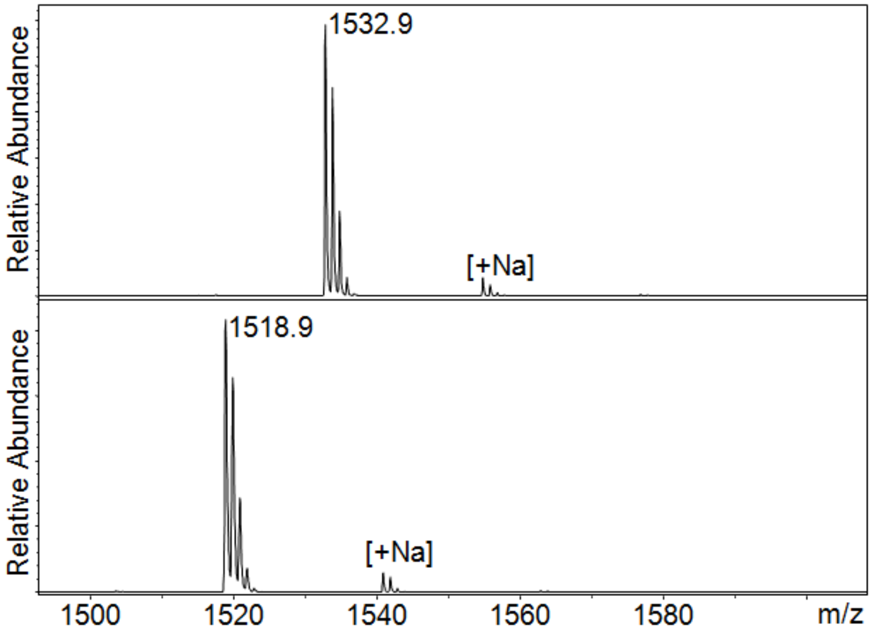

Supplementary Figure S21: MALDI-TOF MS analysis showing no methylation of H3$_{1\text{-}15}$Dab4 peptide (100 µM) in the presence of SETD7 (2 µM) and SAM (200 µM) after incubation for 1 h at 37 °C (top panel). Similar conditions were applied with H3$_{1\text{-}15}$Dap4 peptide (bottom panel).

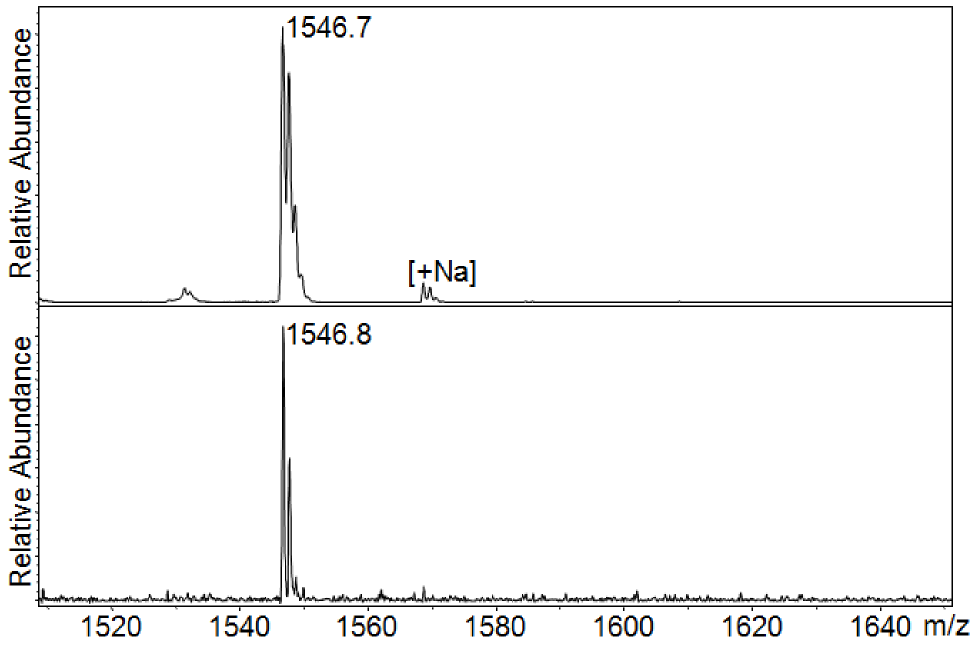

Supplementary Figure S22: MALDI-TOF MS analysis showing no methylation of H3$_{1\text{-}15}$Orn4 peptide (100 µM) in the presence of SETD7 (10 µM) and SAM (1 mM) after incubation for 1 h (top panel) and 6 h (bottom panel) at 37 °C.

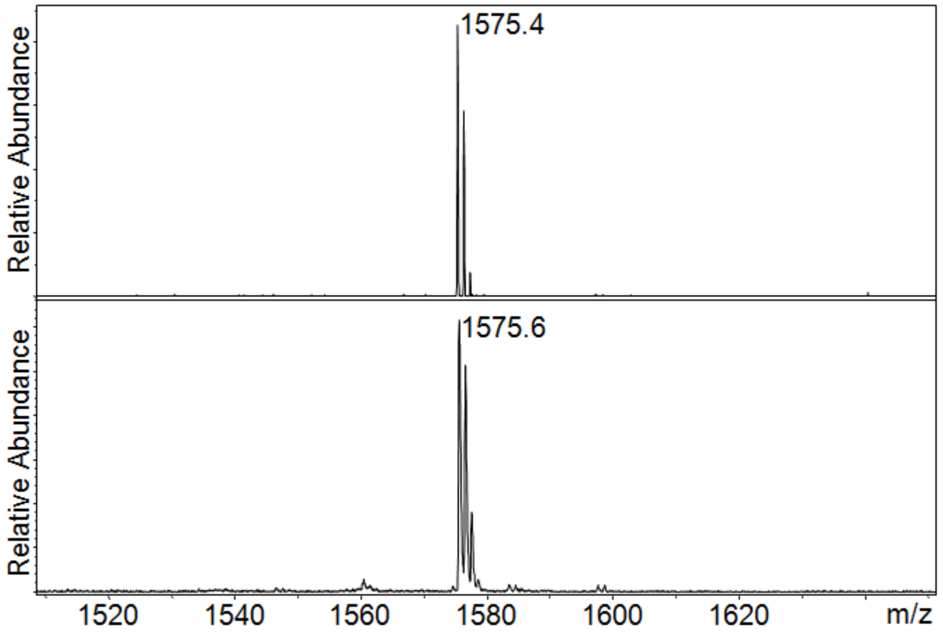

Supplementary Figure S23: MALDI-TOF MS analysis showing no methylation of H3$_{1\text{-}15}$hK4 peptide (100 µM) in the presence of SETD7 (10 µM) and SAM (1 mM) after incubation for 1 h (top panel) and 6 h (bottom panel) at 37 °C.

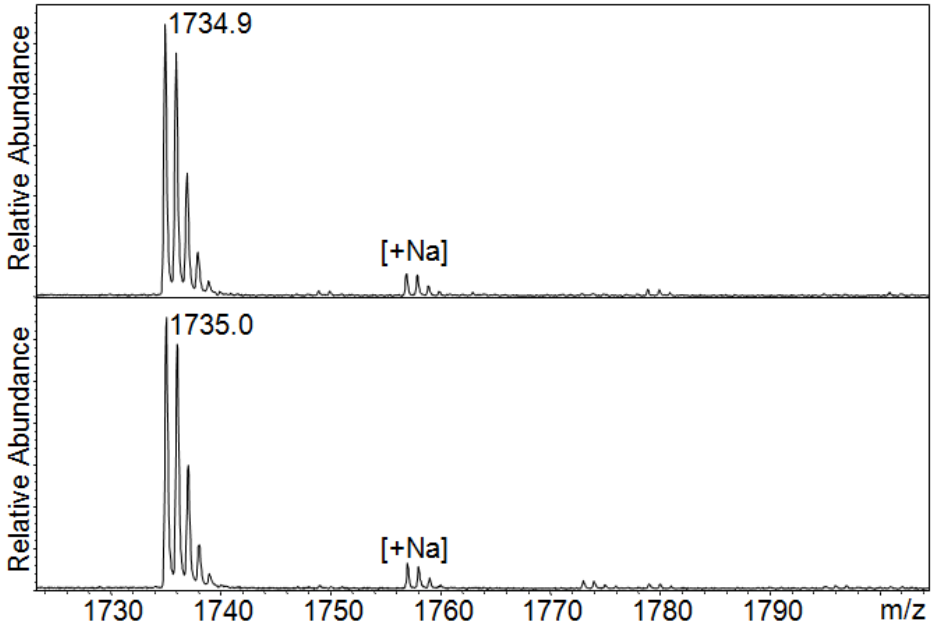

Supplementary Figure S24: MALDI-TOF MS analysis showing no methylation of $H4_{13\text{-}27}$Orn20 peptide (100 µM) in the presence of SETD8 (10 µM) and SAM (1 mM) after incubation for 1 h (top panel) and 6 h (bottom panel) at 37 °C.

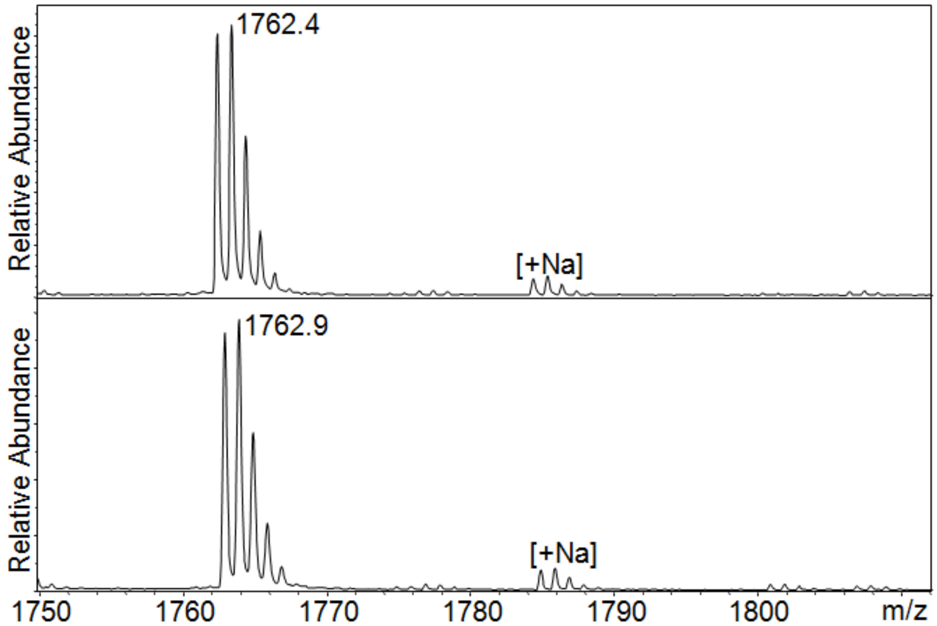

Supplementary Figure S25: MALDI-TOF MS analysis showing no methylation of $H4_{13\text{-}27}$hK20 peptide (100 µM) in the presence of SETD8 (10 µM) and SAM (1 mM) after incubation for 1 h (top panel) and 6 h (bottom panel) at 37 °C.

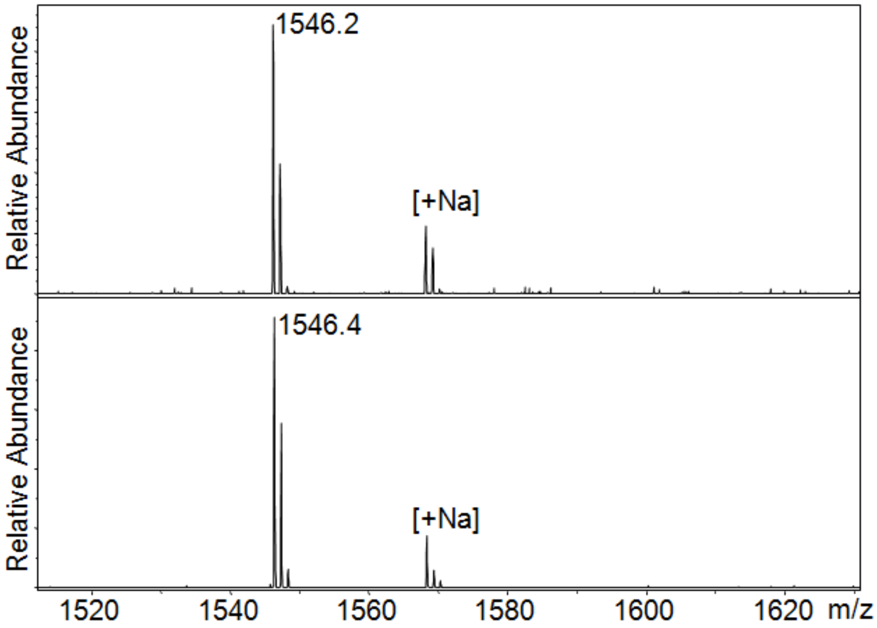

Supplementary Figure S26: MALDI-TOF MS analysis showing no methylation of $H3_{1\text{-}15}$Orn9 peptide (100 µM) in the presence of GLP (10 µM) and SAM (1 mM) after incubation for 1 h (top panel) and 6 h (bottom panel) at 37 °C.

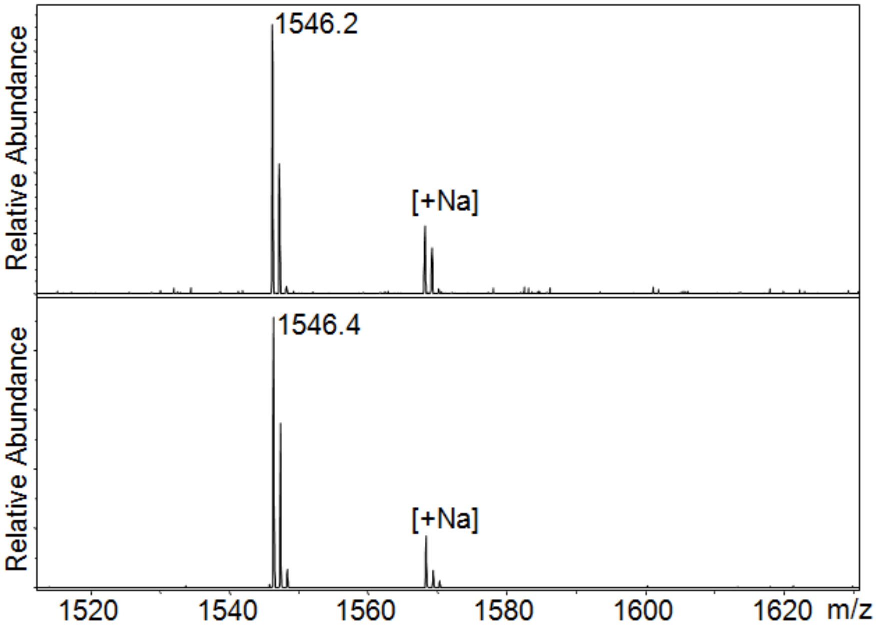

Supplementary Figure S27: MALDI-TOF MS analysis showing no methylation of $H3_{1\text{-}15}$Orn9 peptide (100 µM) in the presence of G9a (10 µM) and SAM (1 mM) after incubation for 1 h (top panel) and 6 h (bottom panel) at 37 °C.

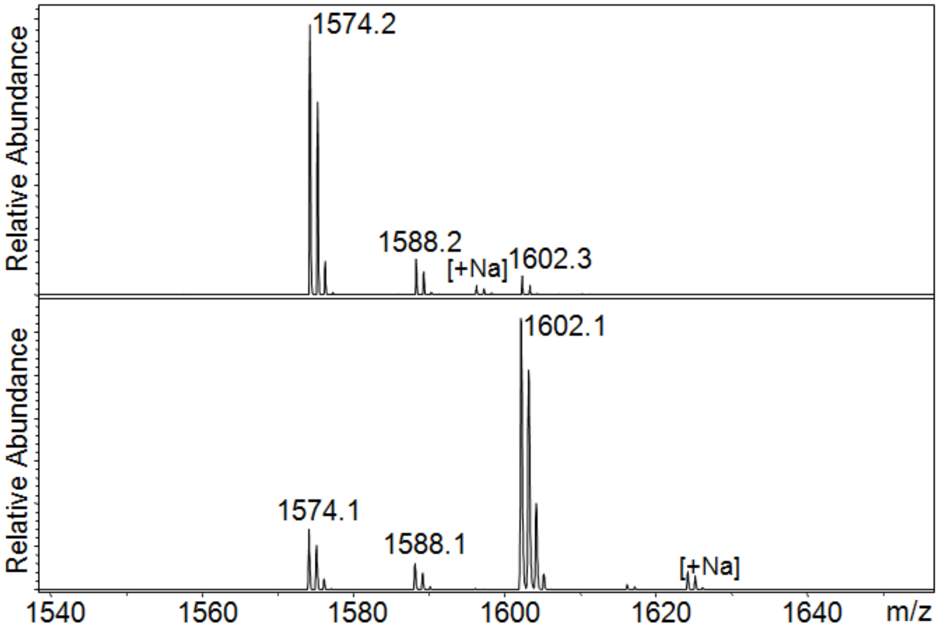

Supplementary Figure S28: MALDI-TOF MS analysis of $H3_{1-15}$hK9 peptide (100 µM) in the presence of GLP (10 µM) and SAM (1 mM) after incubation for 1 h (top panel) and longer incubation for 6 h (bottom panel). Mixtures of unmethylated and monomethylated (+14 Da) and dimethylated (+28 Da) were obtained with both incubations.

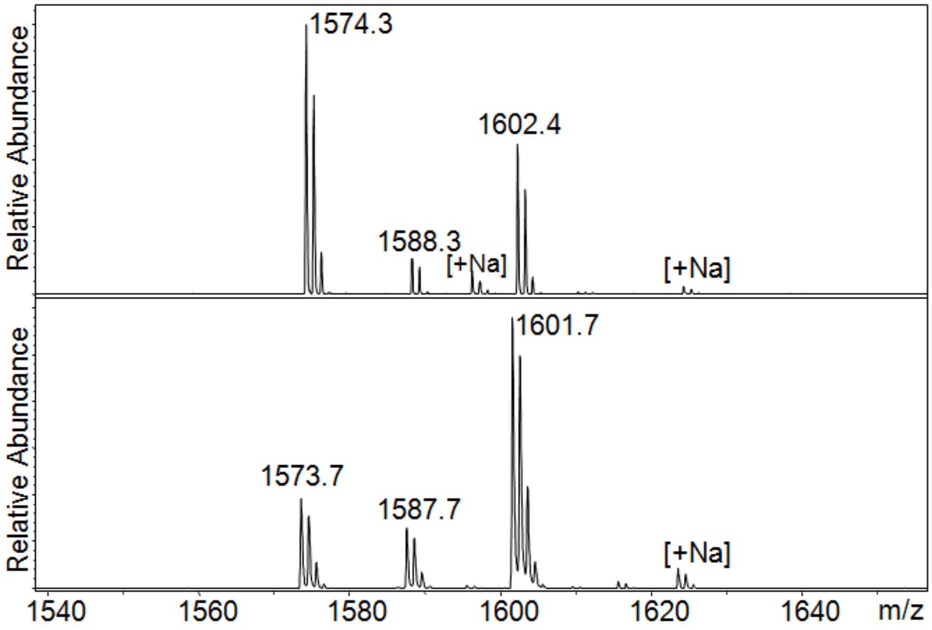

Supplementary Figure S29: MALDI-TOF MS analysis of $H3_{1-15}$hK9 peptide (100 µM) in the presence of G9a (10 µM) and SAM (1 mM) after incubation for 1 h (top panel) and longer incubation for 6 h (bottom panel). Mixtures of unmethylated and monomethylated (+14 Da) and dimethylated (+28 Da) were obtained with both incubations.

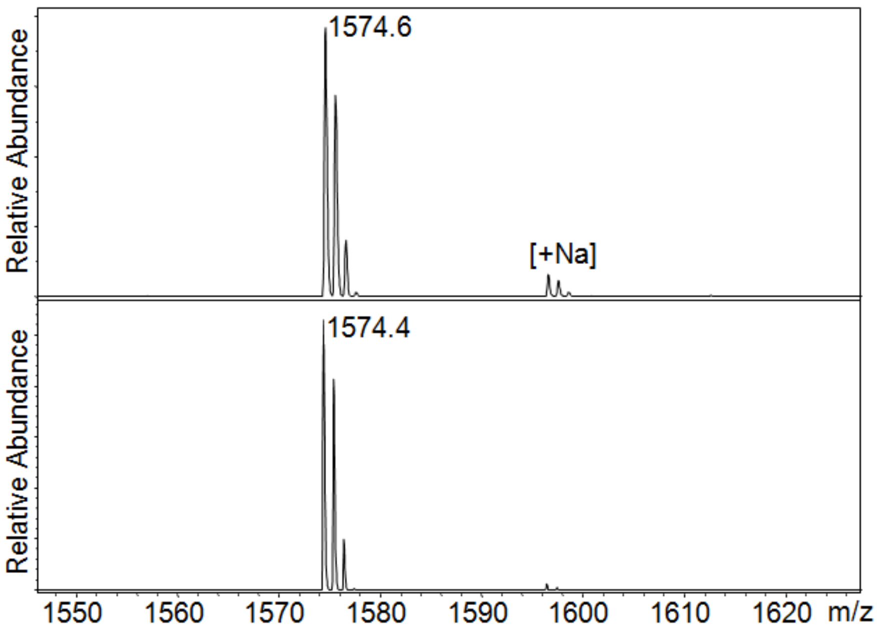

Supplementary Figure S30: MALDI-TOF MS analysis showing no methylation of $H3_{1\text{-}15}$hK9 peptide (100 µM) in the absence of the enzyme and in the presence of SAM (1 mM) after incubation for 1 h (top panel) and 6 h (bottom panel) at 37 °C.

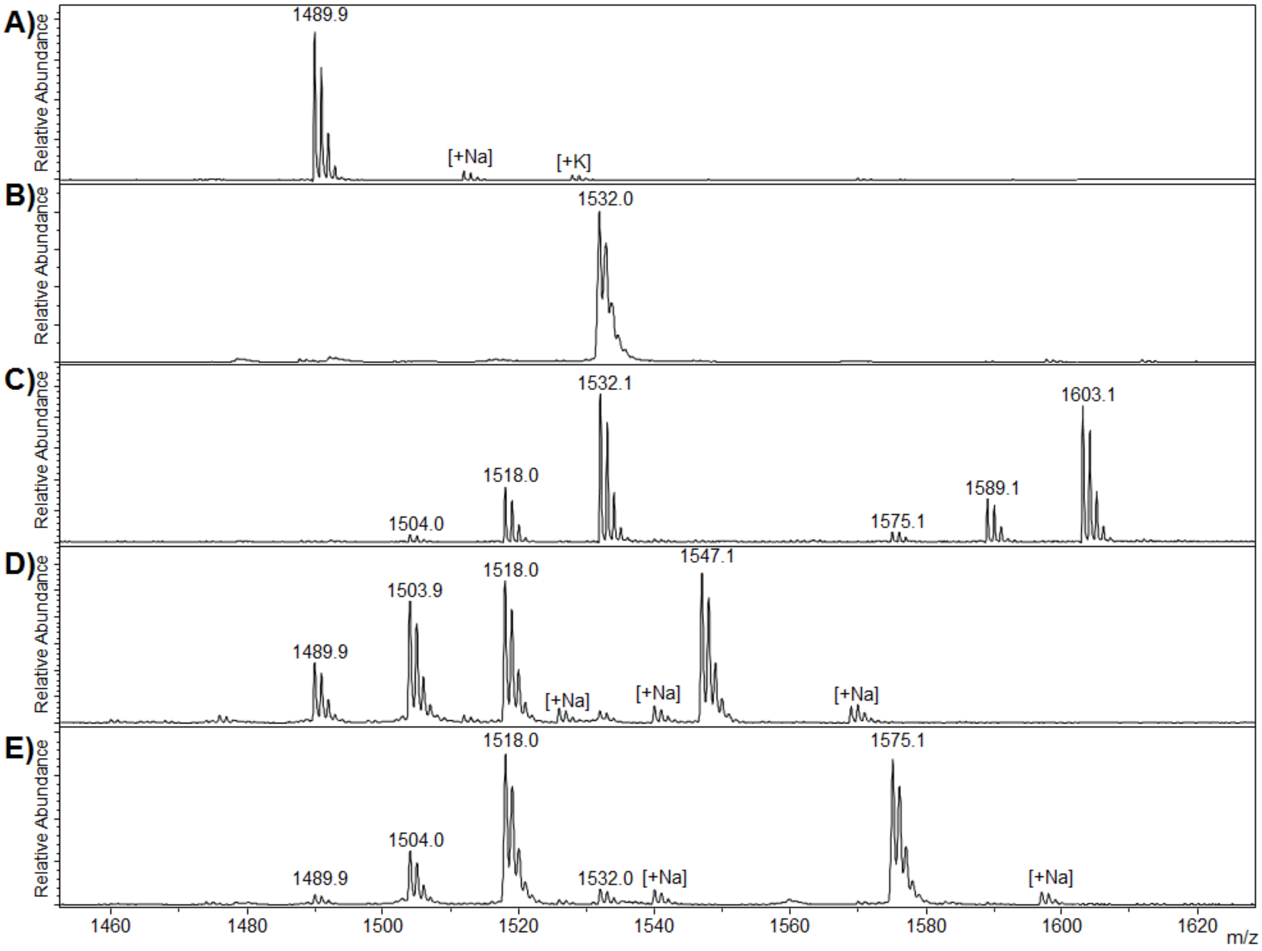

Supplementary Figure S31: MALDI-TOF MS based assay with **A)** H3$_{1\text{-}14}$K9 peptide. **B**) G9a-catalyzed trimethylation of H3$_{1\text{-}14}$K9 in the presence of SAM after 1 h at 37 °C. **C**) Competition experiment for G9a-catalyzed methylation of H3$_{1\text{-}14}$K9 (100 µM) and H3$_{1\text{-}15}$K9 (100 µM) in the presence of SAM (500 µM) after 1 h at 37 °C. **D**) G9a-catalyzed methylation of H3$_{1\text{-}14}$K9 (100 µM) in the presence of H3$_{1\text{-}15}$Orn9 (100 µM) and SAM (500 µM) after 1 h at 37 °C. **E**) G9a-catalyzed methylation of H3$_{1\text{-}14}$K9 (100 µM) in the presence of H3$_{1\text{-}15}$hK9 (100 µM) and SAM (500 µM) after 1 h at 37 °C.

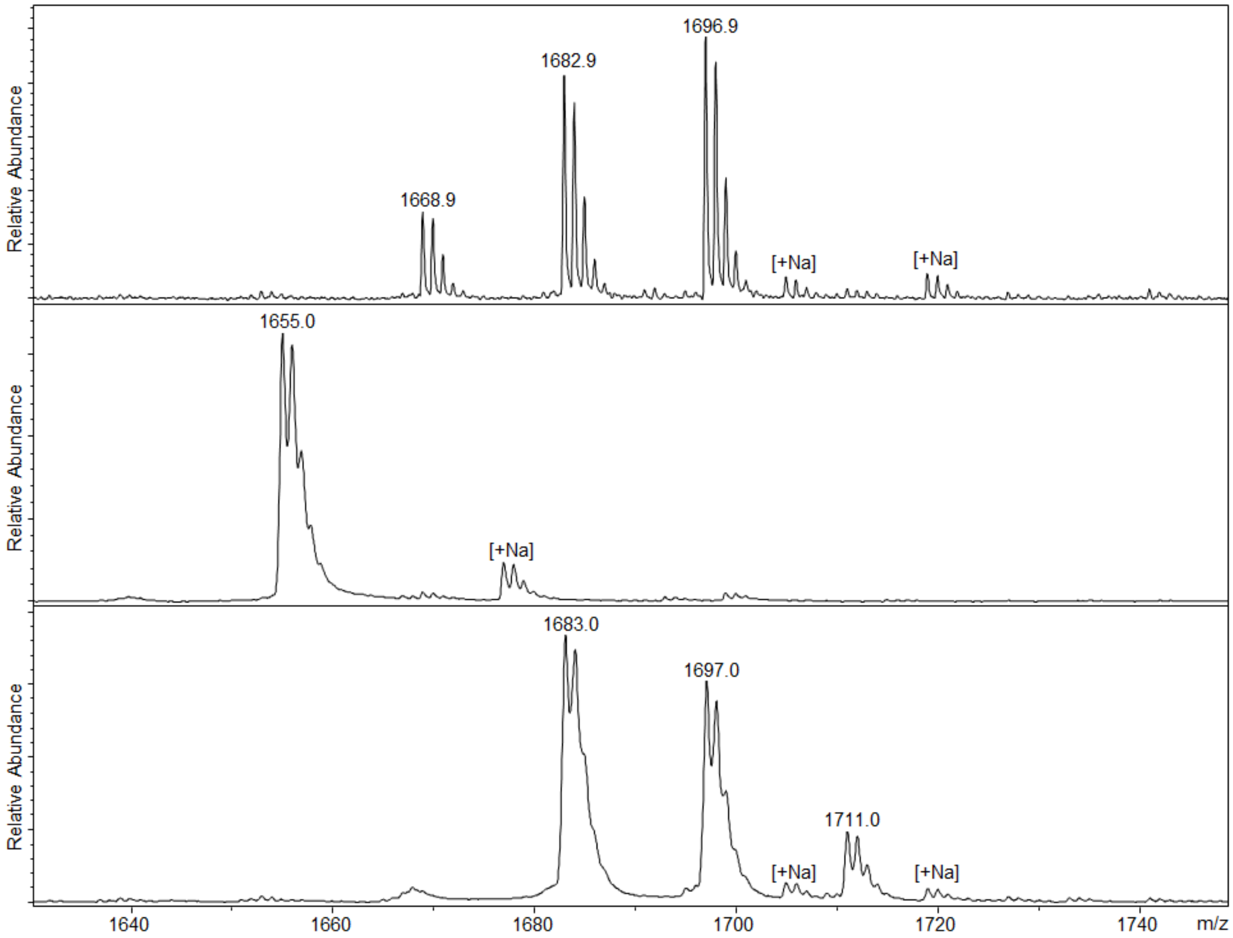

Supplementary Figure S32: GLP (10 µM)-catalyzed methylation of p53 peptides (100 µM) in the presence of SAM (2 mM) after 3 h at 37 °C as monitored by MALDI-TOF MS. GLP with $p53_{365\text{-}379}$K373 (top, Mw 1668.9), $p53_{365\text{-}379}$Orn373 (middle, Mw 1654.9), $p53_{365\text{-}379}$hK373 (bottom, Mw 1682.9). Mixtures of unmethylated starting material, and monomethylated (+ 14 Da) and dimethylated (+ 28 Da) products were obtained upon incubation with $p53_{365\text{-}379}$K373 and $p53_{365\text{-}379}$hK373.

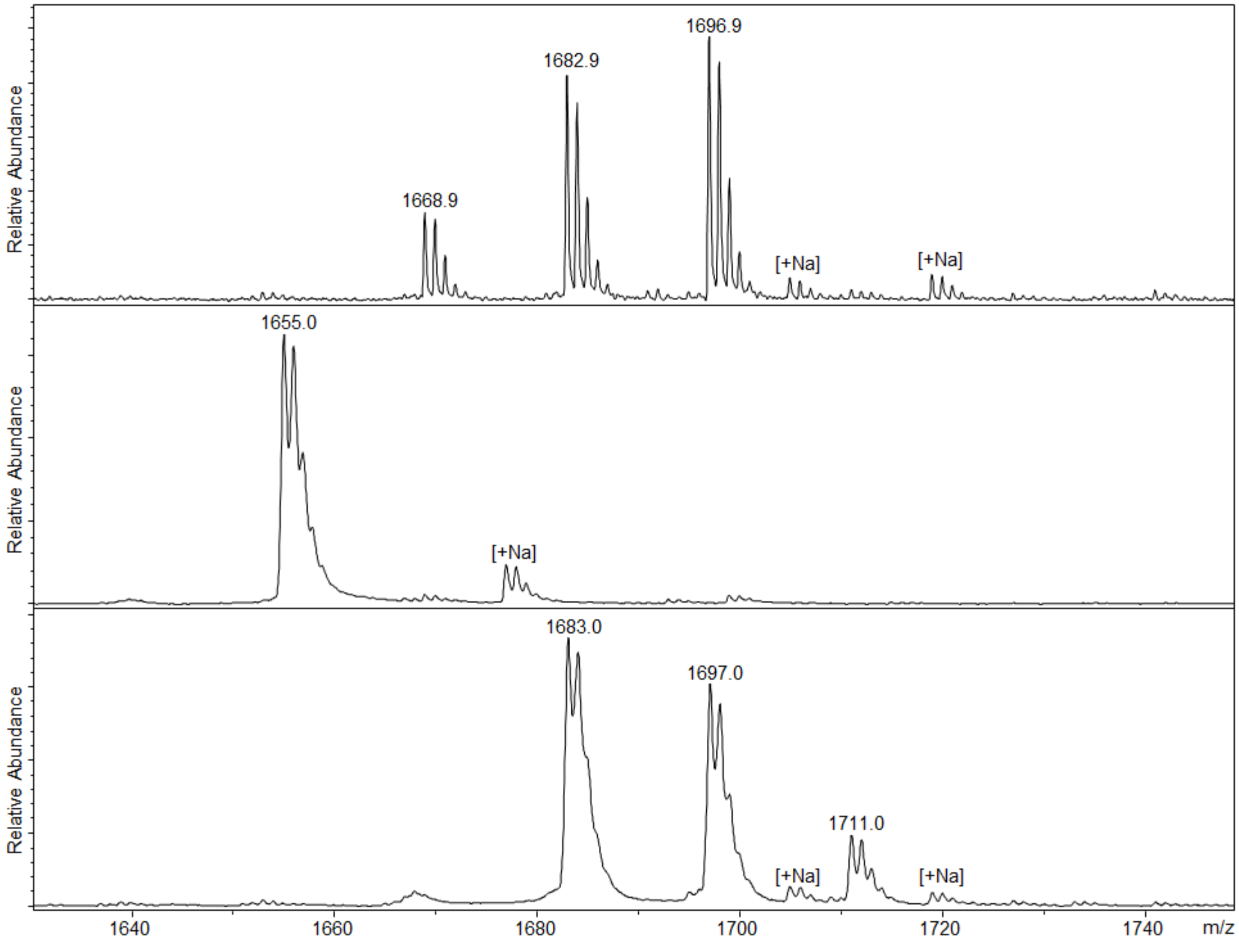

Supplementary Figure S33: G9a (10 µM)-catalyzed methylation of p53 peptides (100 µM) in the presence of SAM (2 mM) after 3 h at 37 °C as monitored by MALDI-TOF MS. GLP with $p53_{365\text{-}379}$K373 (top, Mw 1668.9), $p53_{365\text{-}379}$Orn373 (middle, Mw 1655.0), $p53_{365\text{-}379}$hK373 (bottom, Mw 1683.0). Mixtures of unmethylated starting material, and monomethylated (+ 14 Da) and dimethylated (+ 28 Da) products were obtained upon incubation with $p53_{365\text{-}379}$K373 and $p53_{365\text{-}379}$hK373.

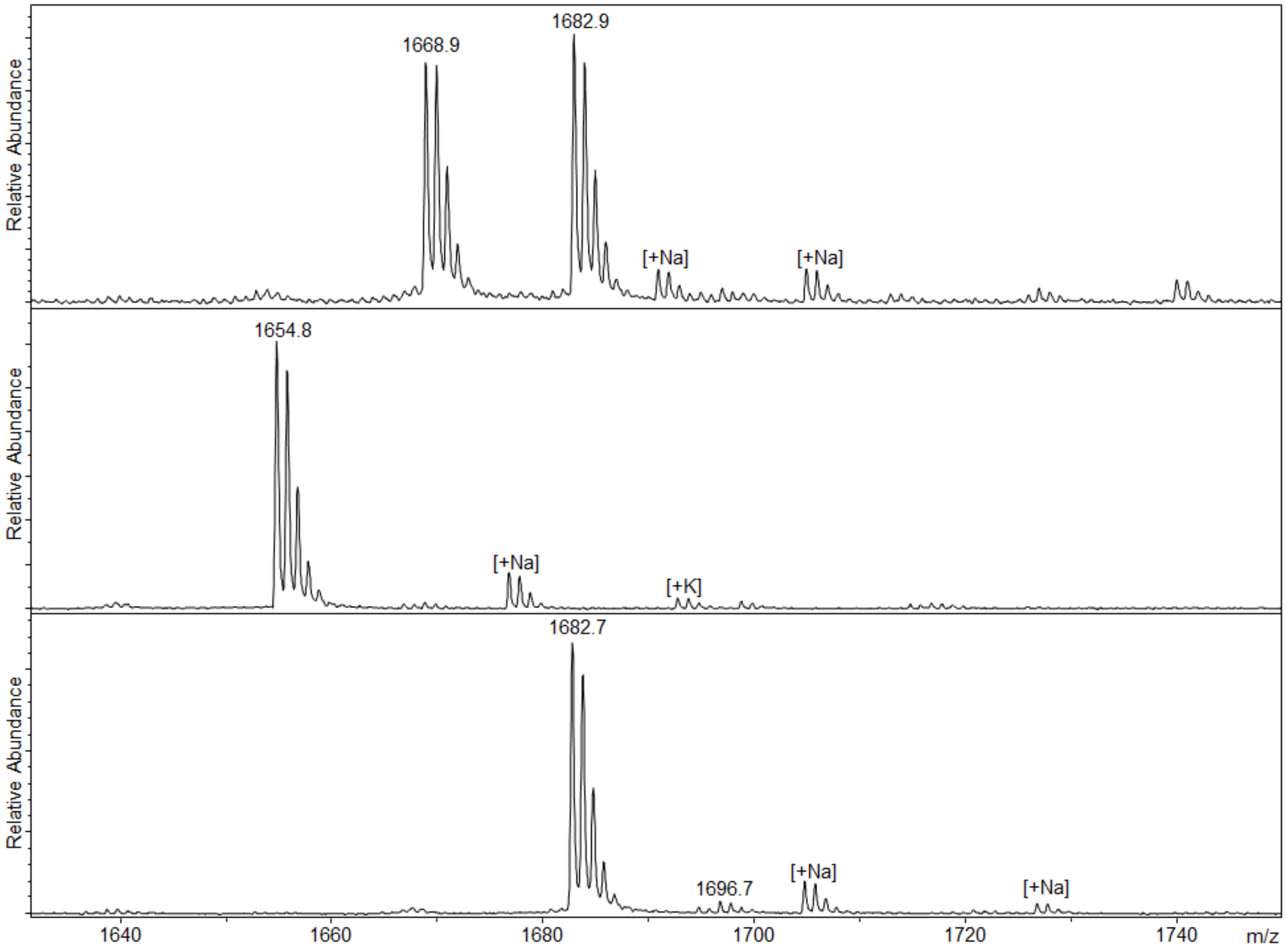

Supplementary Figure S34: SETD7 (10 µM)-catalyzed methylation of p53 peptides (100 µM) in the presence of SAM (2 mM) after 3 h at 37 °C as monitored by MALDI-TOF MS. SETD7 with p53$_{365-379}$K372 (top, Mw 1668.9), p53$_{365-379}$Orn372 (middle, Mw 1654.8), p53$_{365-379}$hK372 (bottom, Mw 1682.7).

4. NMR supplementary figures

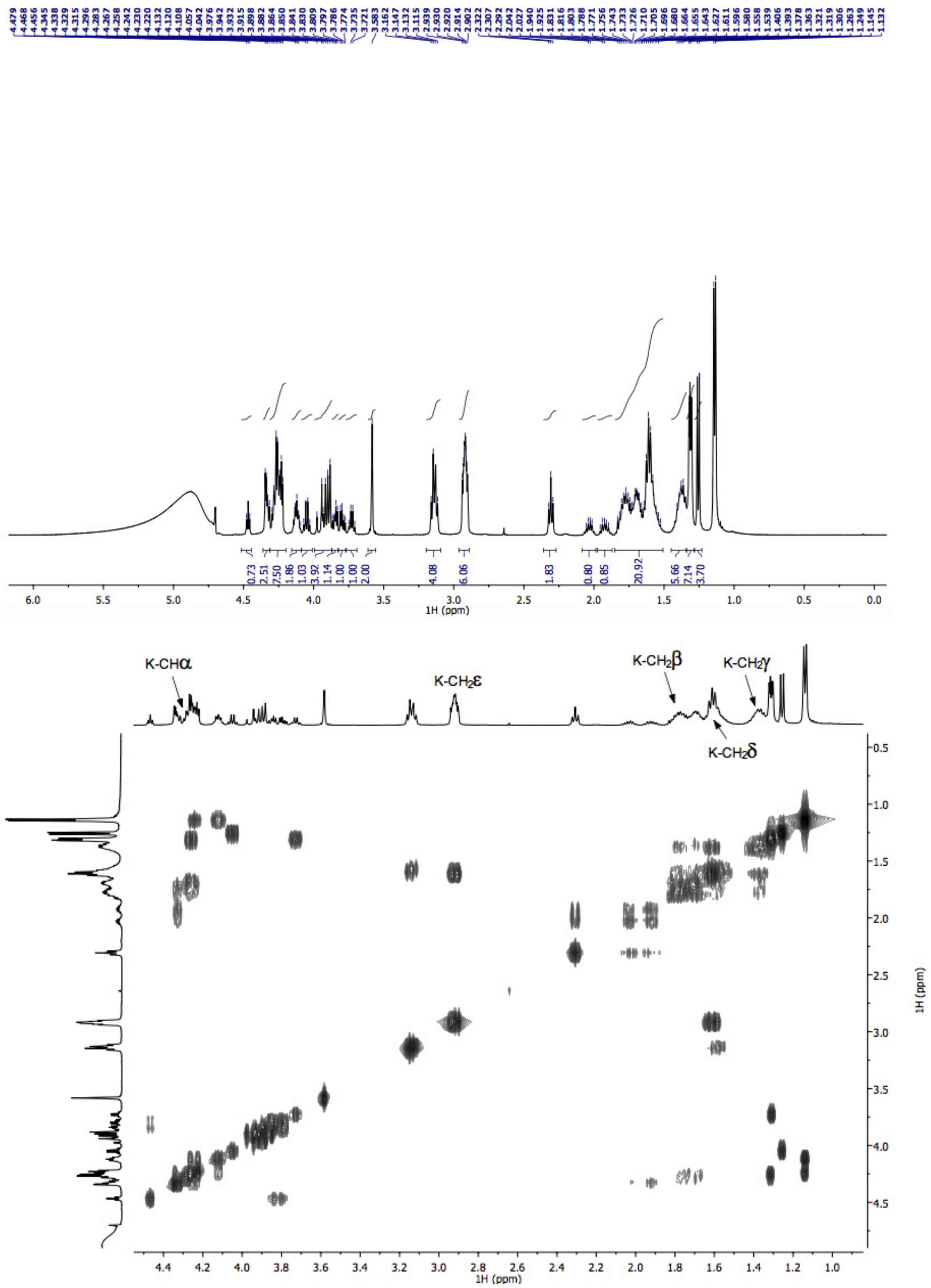

Supplementary Figure S35: Presat ^{1}H NMR data of H3K9 peptide (top). ^{1}H-^{1}H presatCOSY data of H3K9 peptide (bottom).

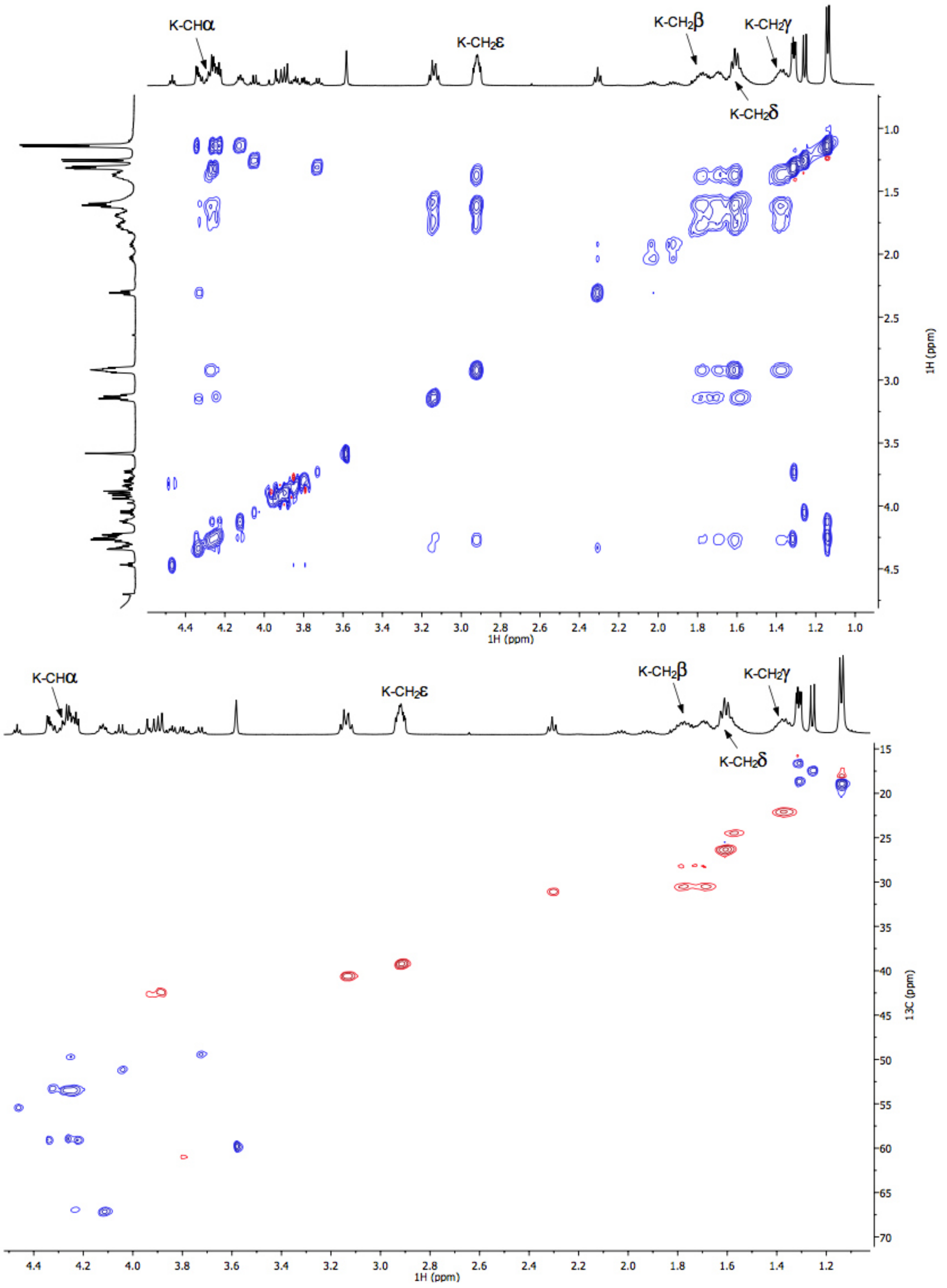

Supplementary Figure S36: 2D TOCSY data of H3K9 peptide (top). Multiplicity-edited HSQC data of H3K9 peptide (bottom; blue = positive, CH/CH_3, red = negative, CH_2).

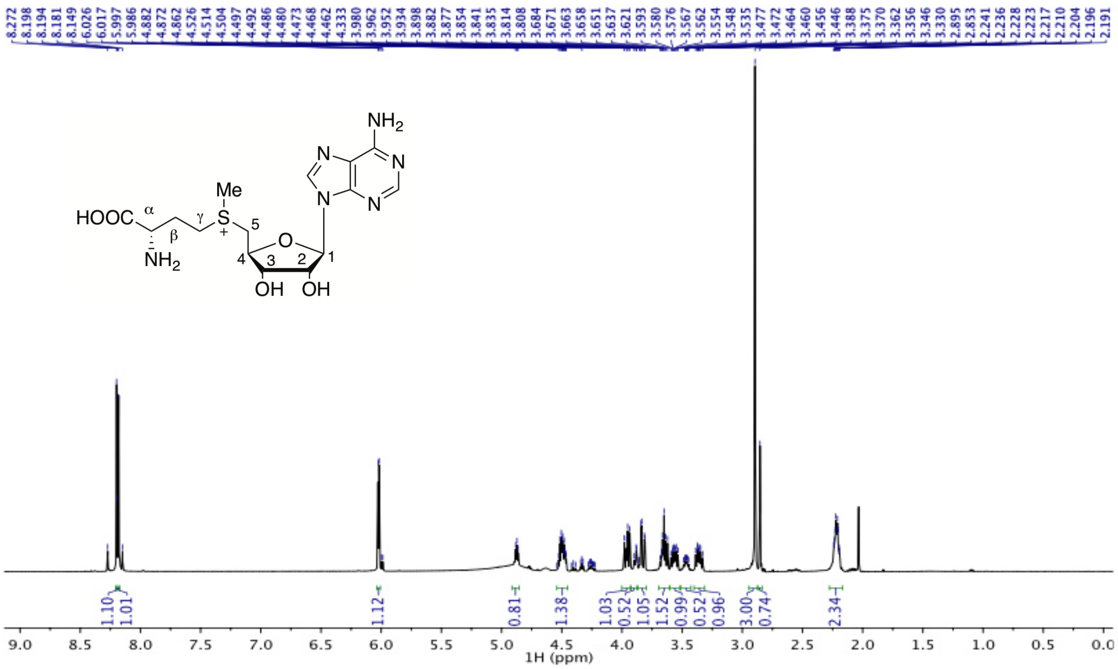

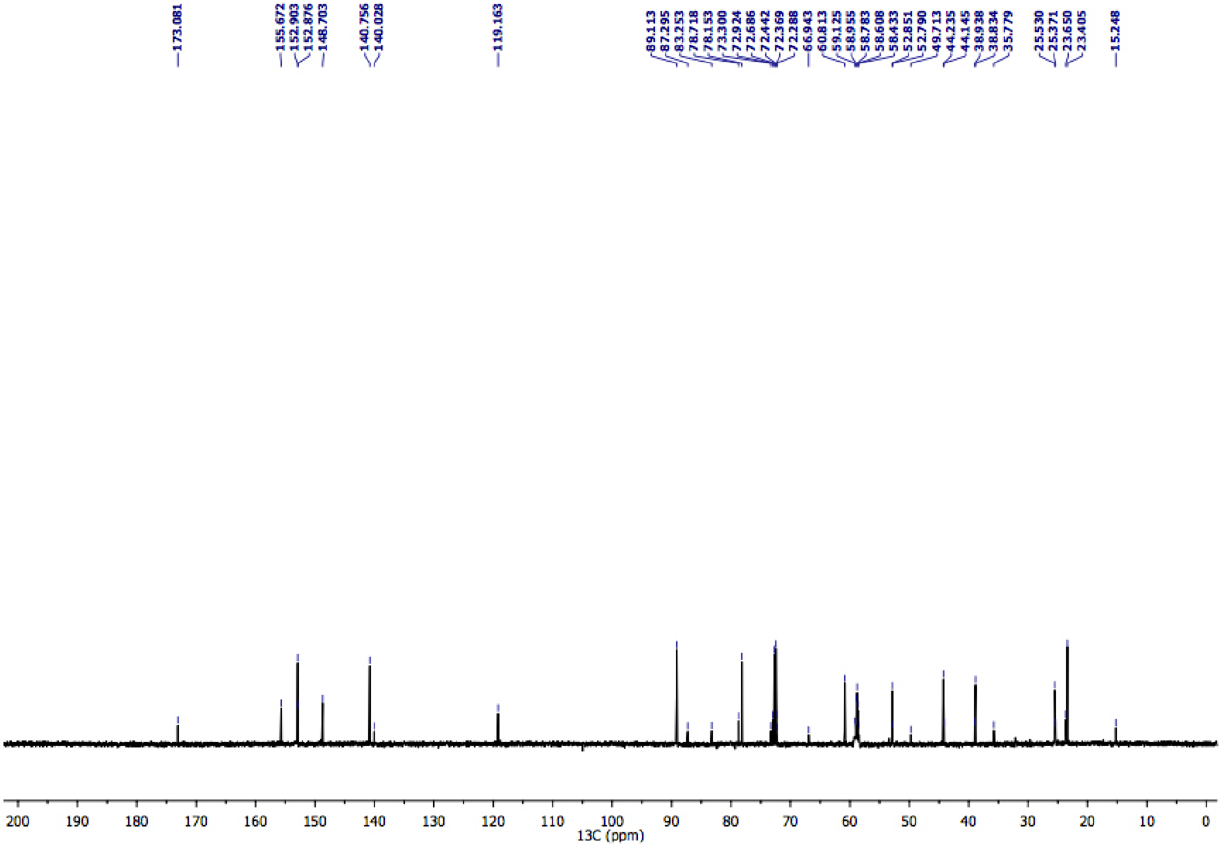

Supplementary Figure S37: Presat ^{1}H NMR data of SAM (top). ^{13}C NMR data of SAM (bottom).

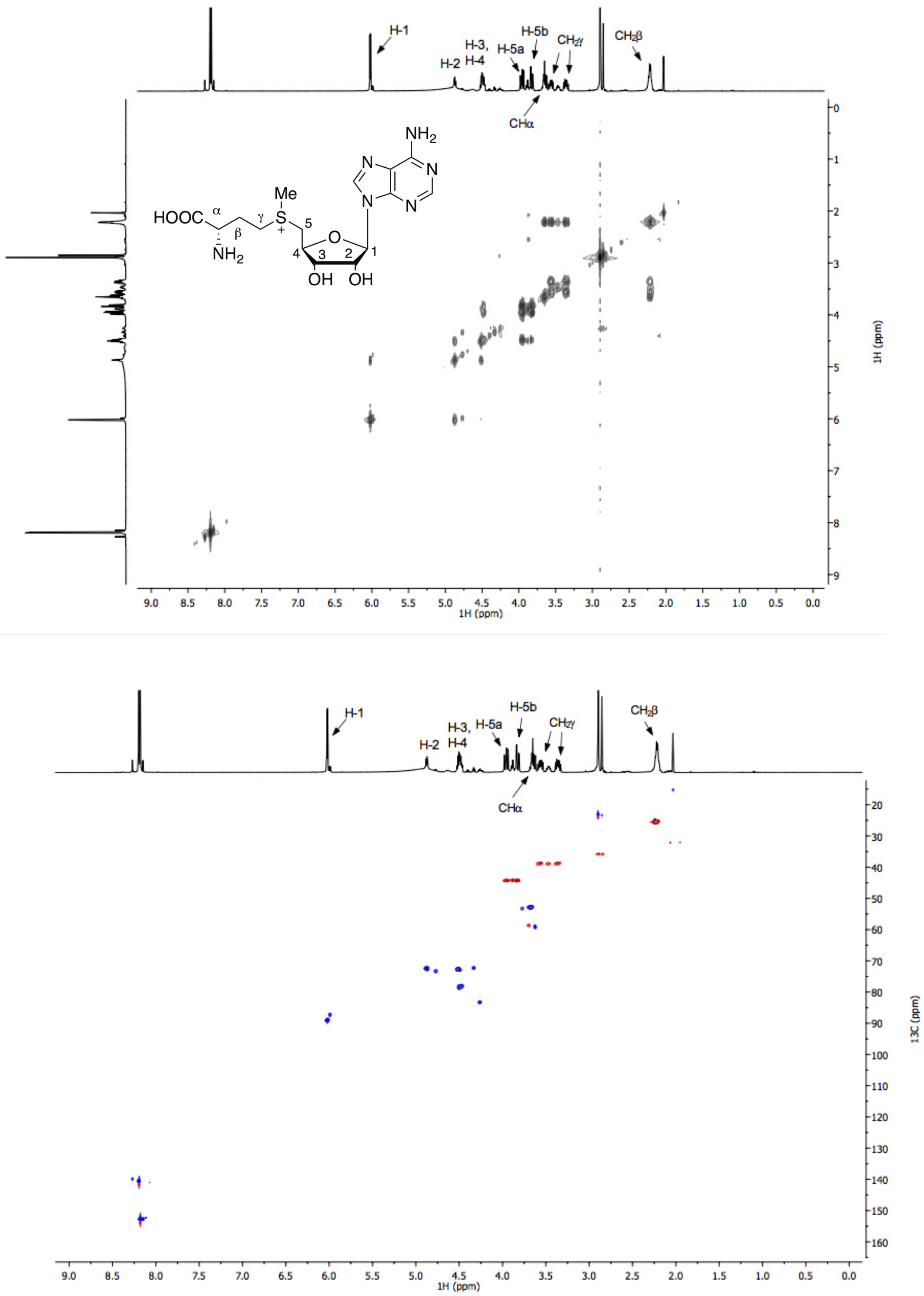

Supplementary Figure S38: ^{1}H-^{1}H presatCOSY data of SAM (top). Multiplicity-edited HSQC data of SAM (bottom; blue = positive, CH/CH_3, red = negative, CH_2).

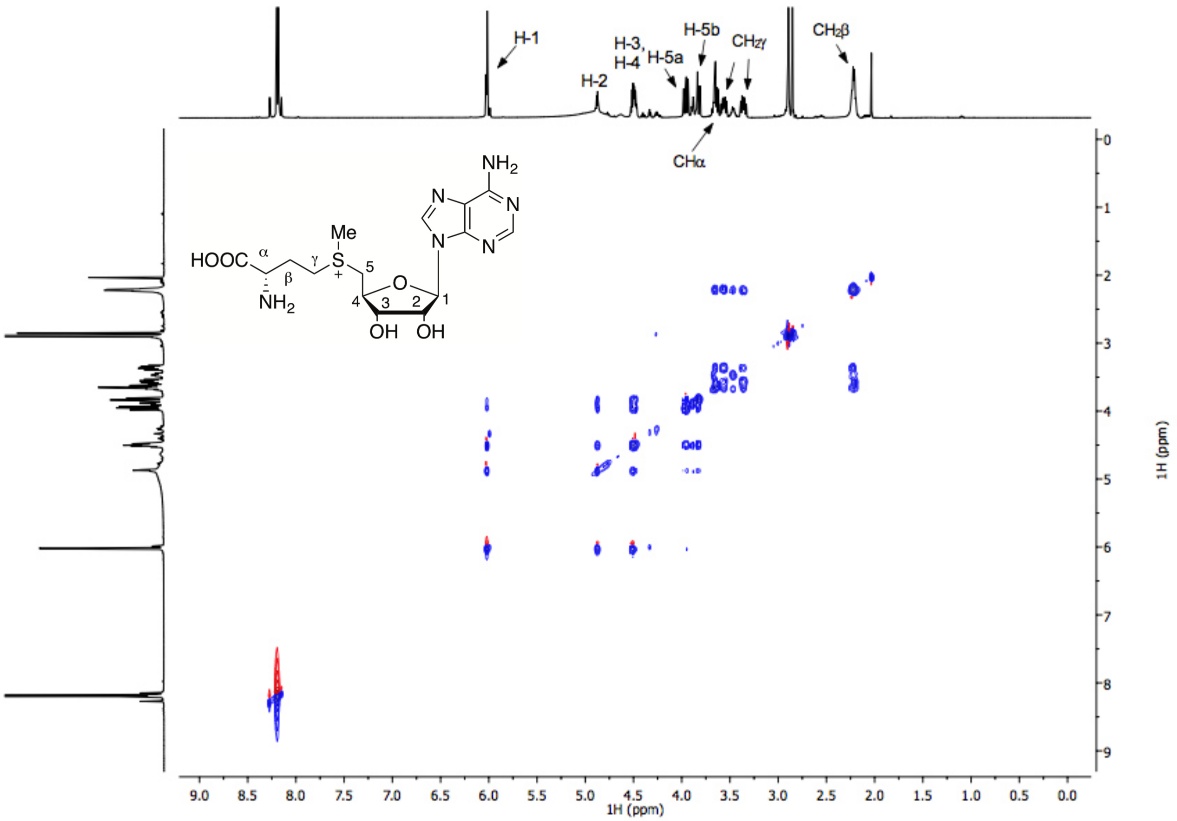

Supplementary Figure S39: 2D TOCSY data of SAM.

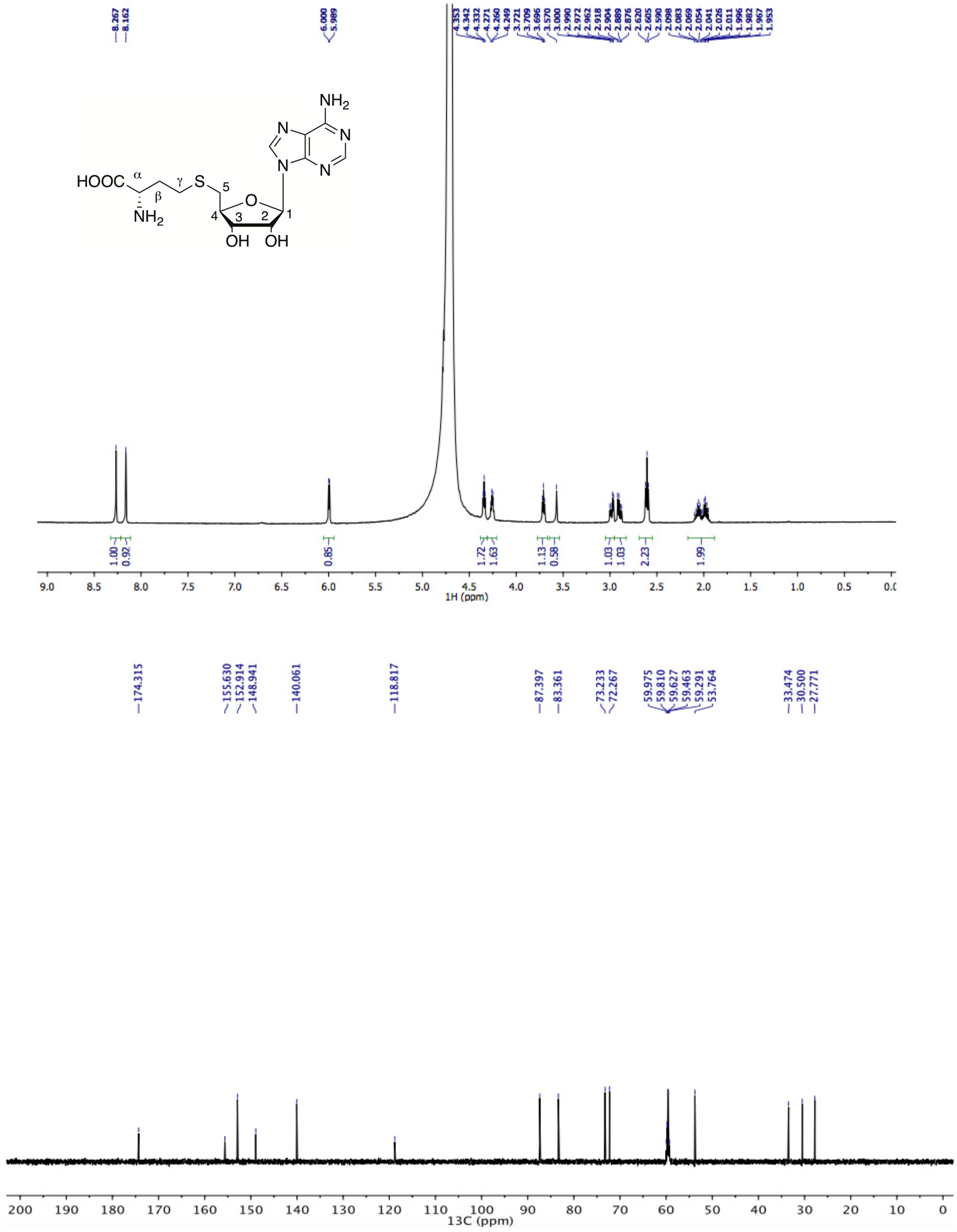

Supplementary Figure S40: Presat ^{1}H NMR data of SAH (top). ^{13}C NMR data of SAH (bottom).

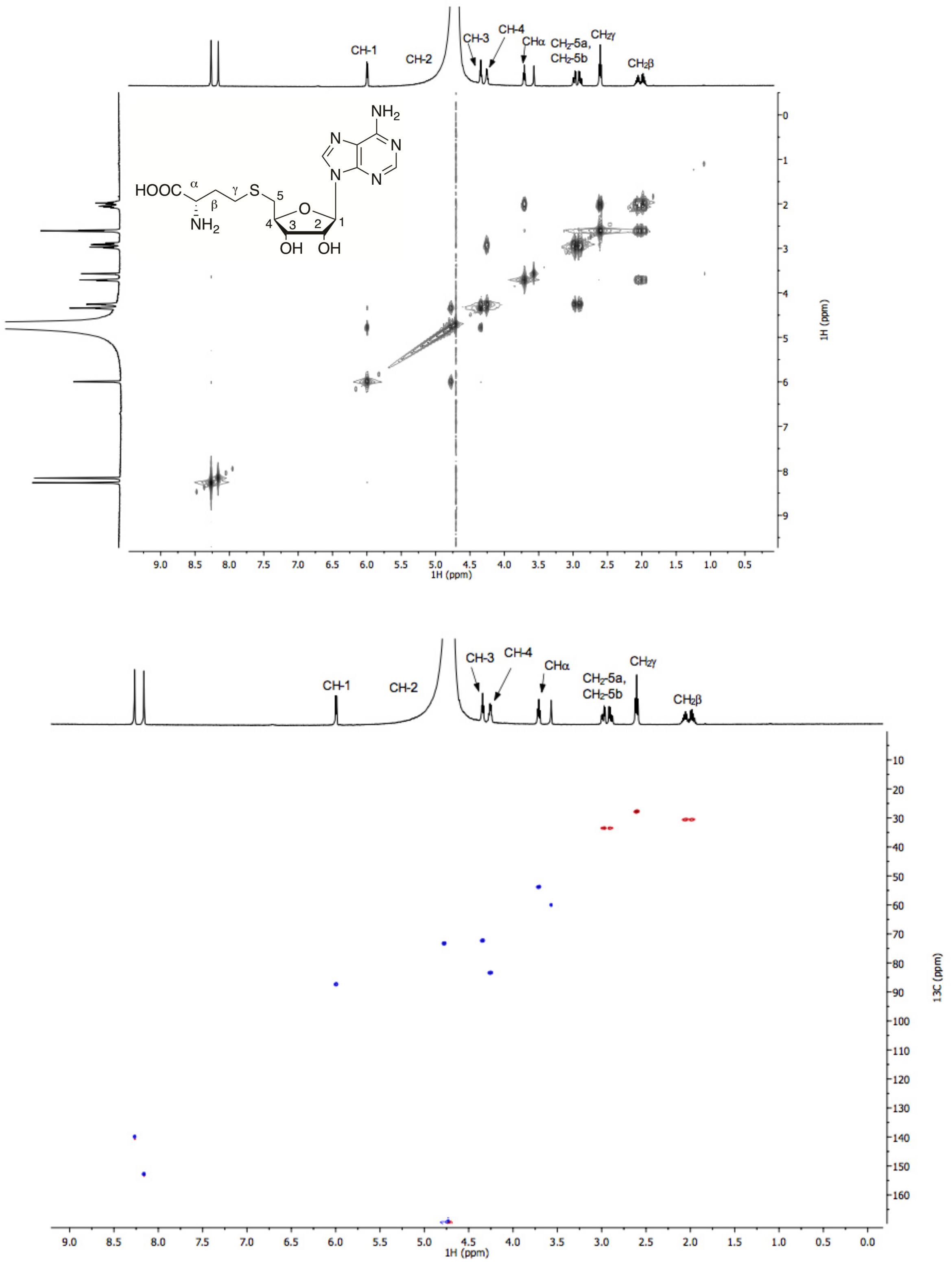

Supplementary Figure S41: ^{1}H-^{1}H presatCOSY data of SAH (top). Multiplicity-edited HSQC data of SAH (bottom; blue = positive, CH/CH_3, red = negative, CH_2).

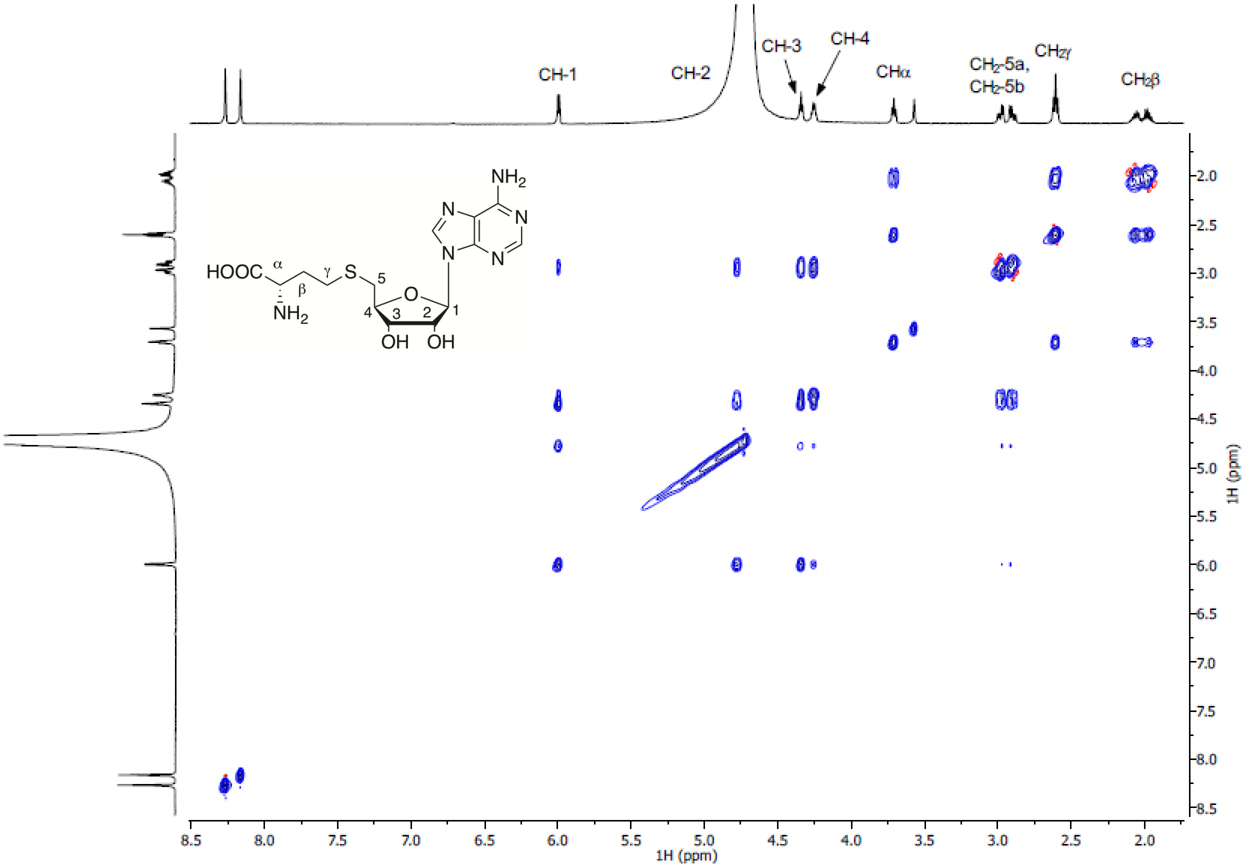

Supplementary Figure S42: 2D TOCSY data of SAH.

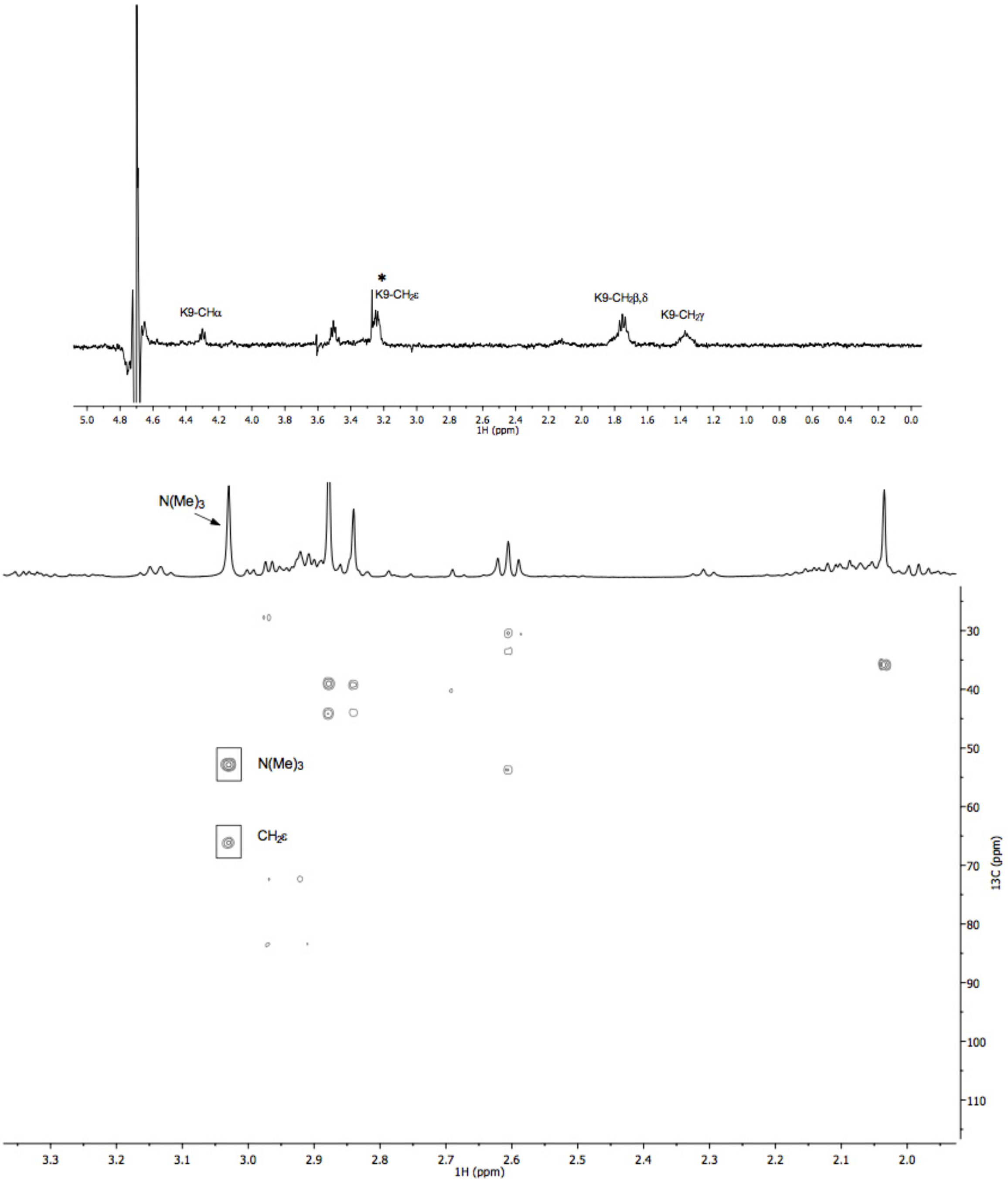

Supplementary Figure S43: 1D TOCSY data of GLP-catalyzed trimethylation of H3K9 peptide with mixing time of 100 ms; * selective excitation at 3.24ppm (top). HMBC data of GLP-catalyzed trimethylation of H3K9 peptide (bottom).

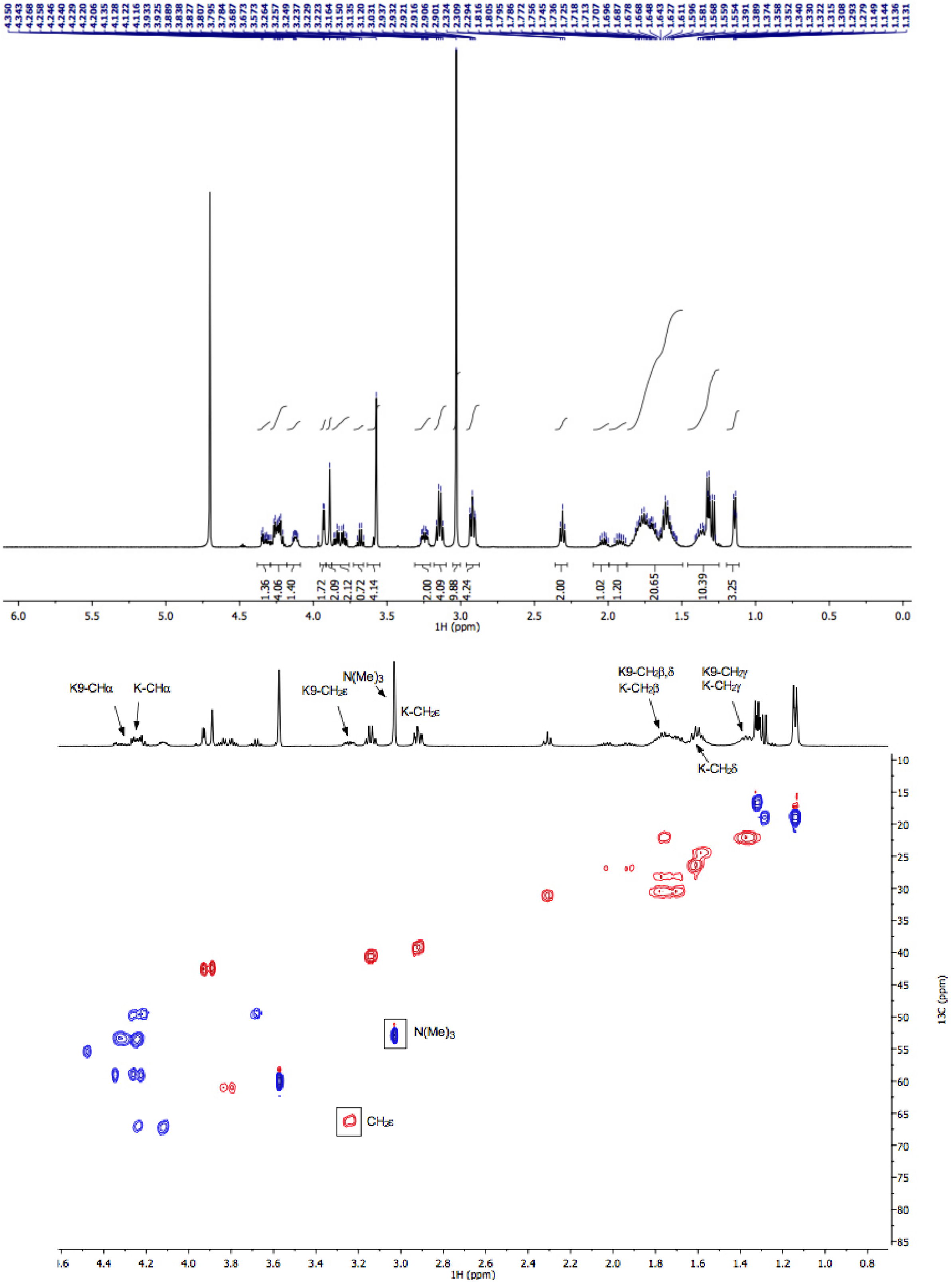

Supplementary Figure S44: Presat ^{1}H NMR data of H3K9me3 peptide (top). Multiplicity-edited HSQC data of H3K9me3 peptide (bottom; blue = positive, CH/CH_3, red = negative, CH_2).

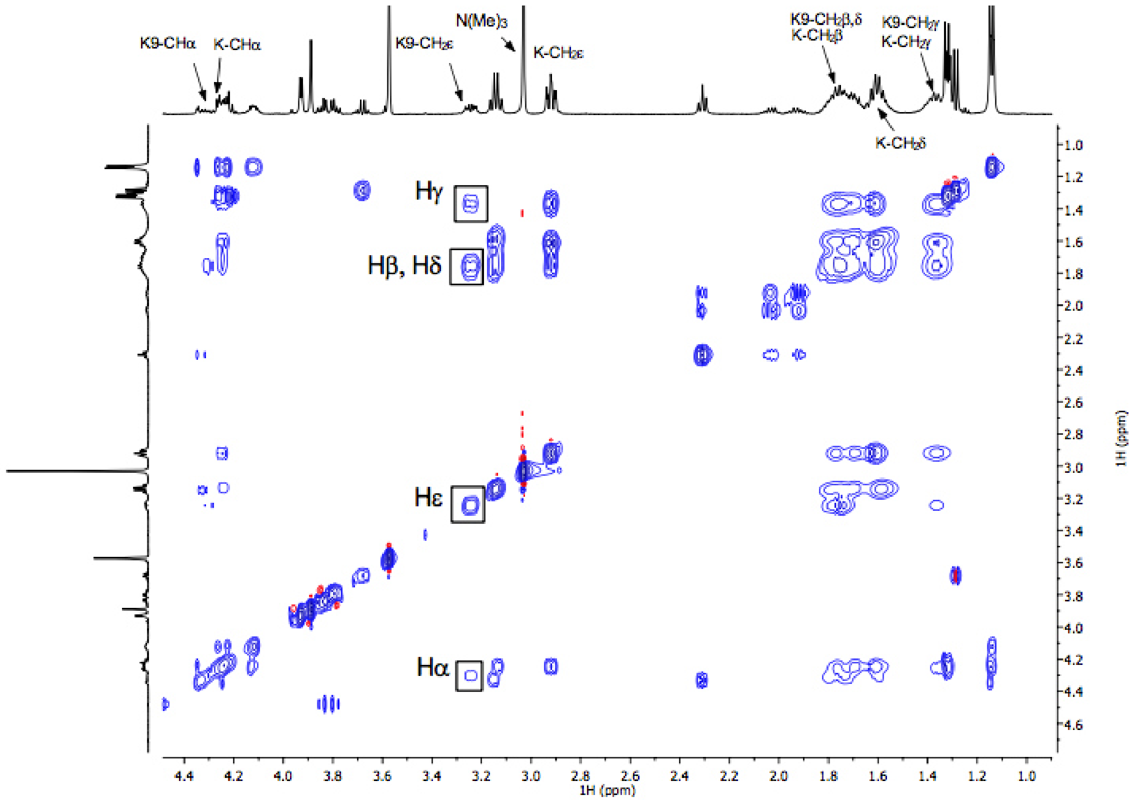

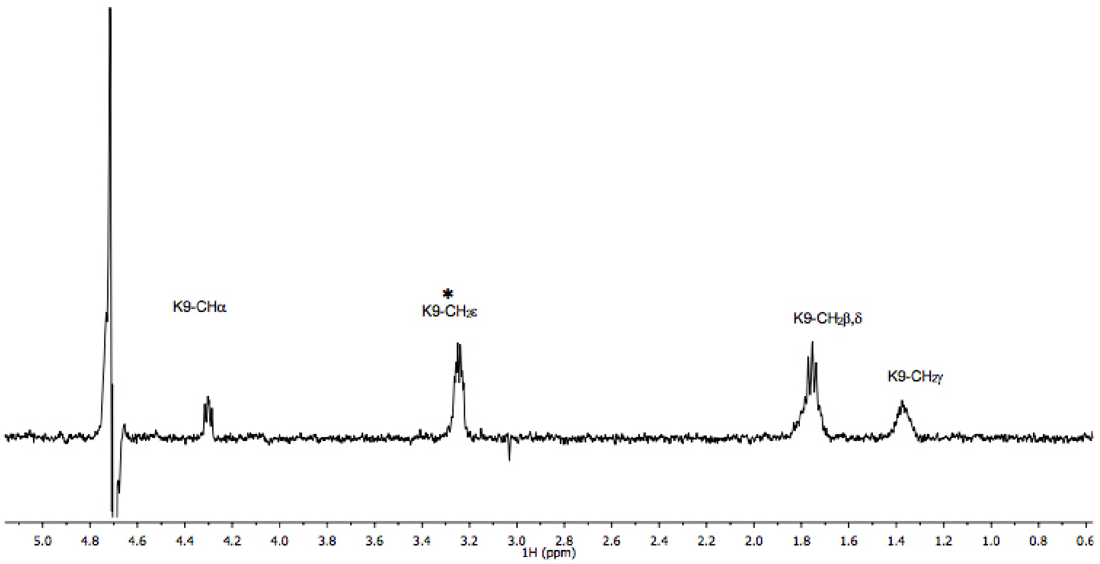

Supplementary Figure S45: 2D TOCSY data of H3K9me3 peptide (top). 1D TOCSY data of H3K9me3 peptide with mixing time of 100 ms; * selective excitation at 3.24 ppm (bottom).

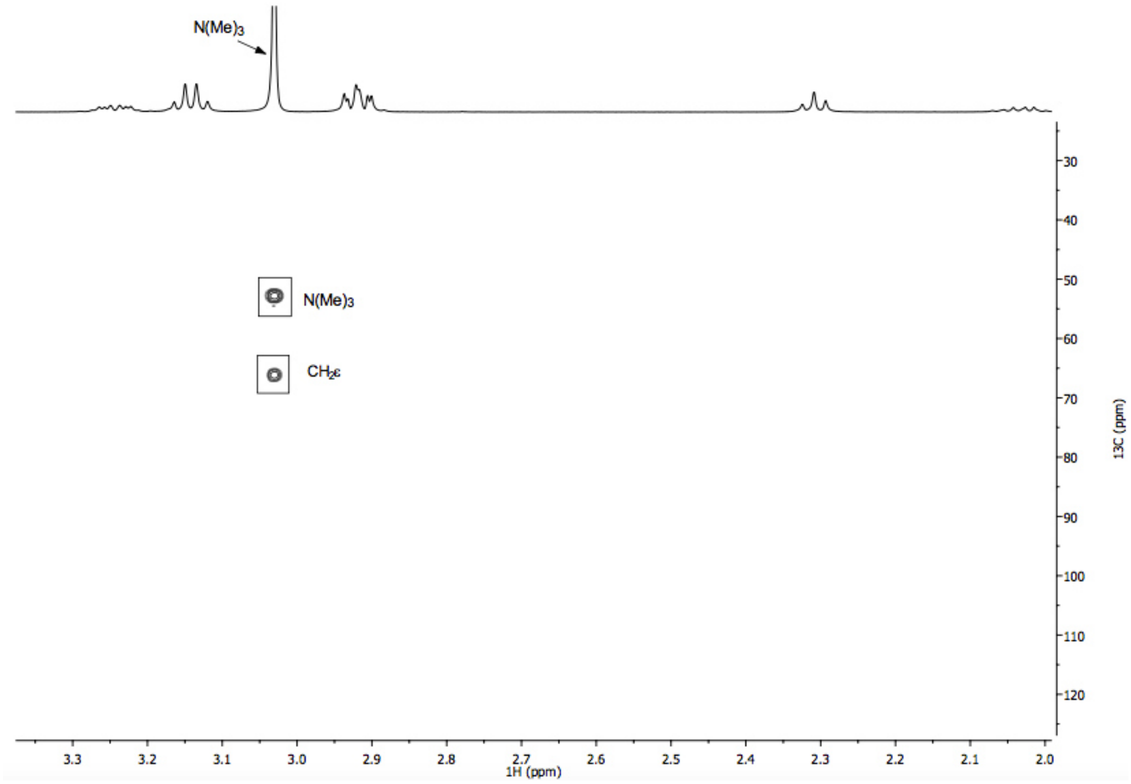

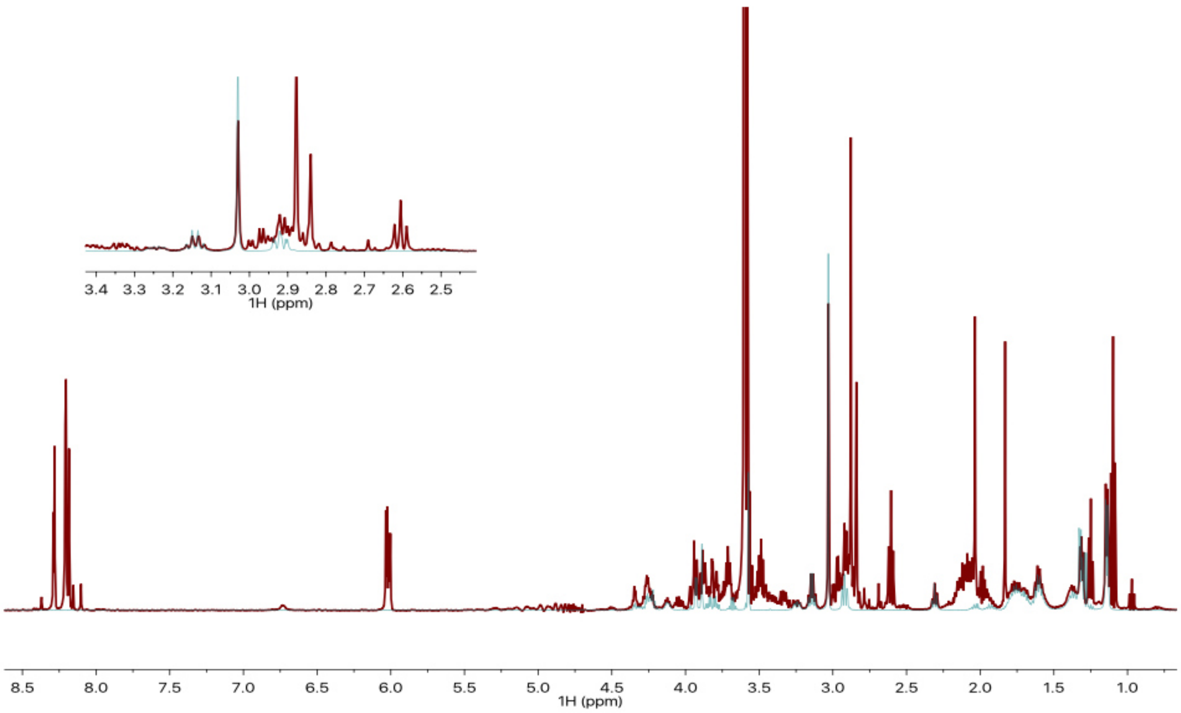

Supplementary Figure S46: HMBC data of H3K9me3 peptide (top). Stacked Presat ^{1}H NMR spectra of GLP-catalyzed trimethylation of H3K9 peptide in red with the synthetic H3K9me3 peptide in green (bottom).

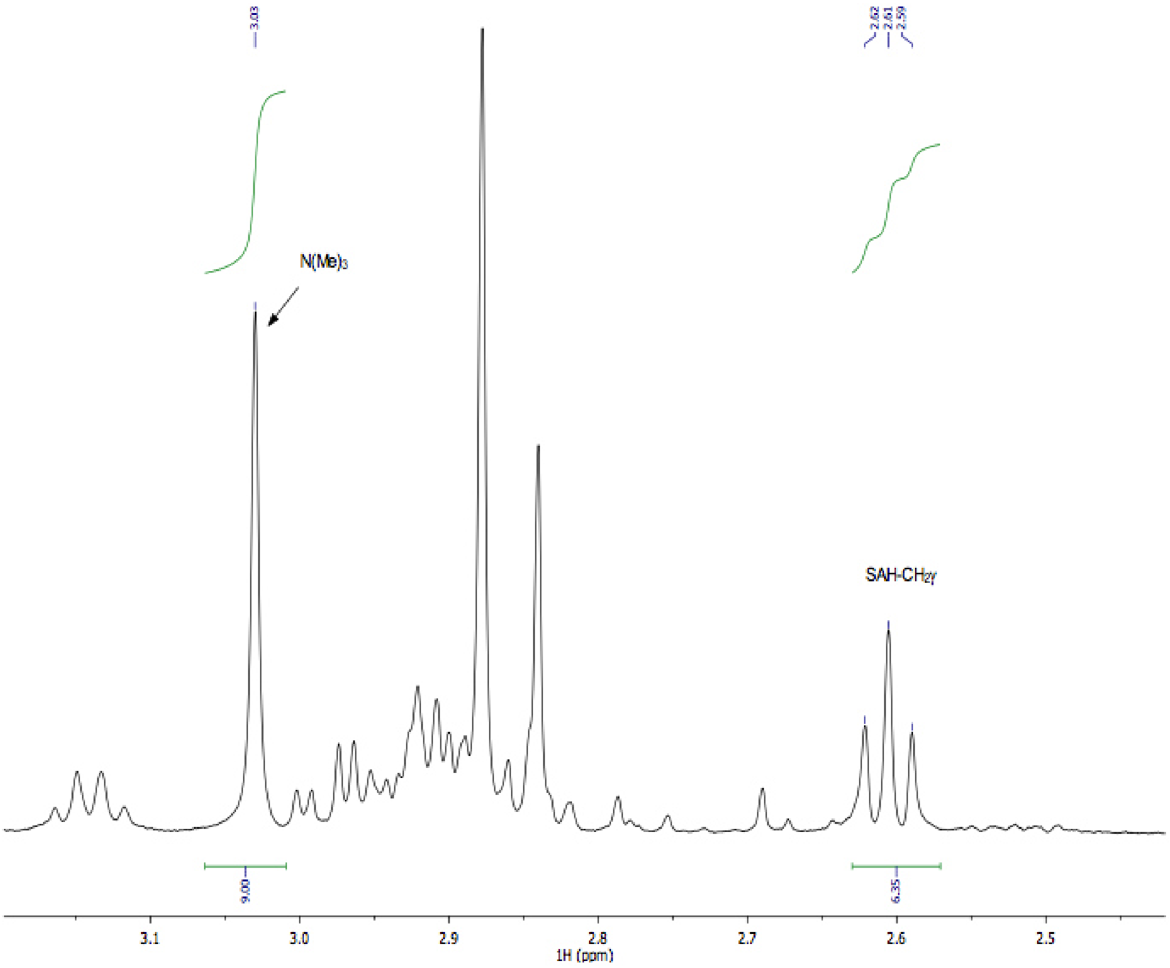

Supplementary Figure S47: A zoomed view on the ^{1}H NMR spectra of GLP-catalyzed trimethylation of H3K9 peptide (9:6 ratio of $N(Me)_3$: SAH-$CH_2\gamma$ indicates that conversion of SAM to SAH is tightly coupled to the GLP-catalyzed trimethylation of H3K9 peptide).

.

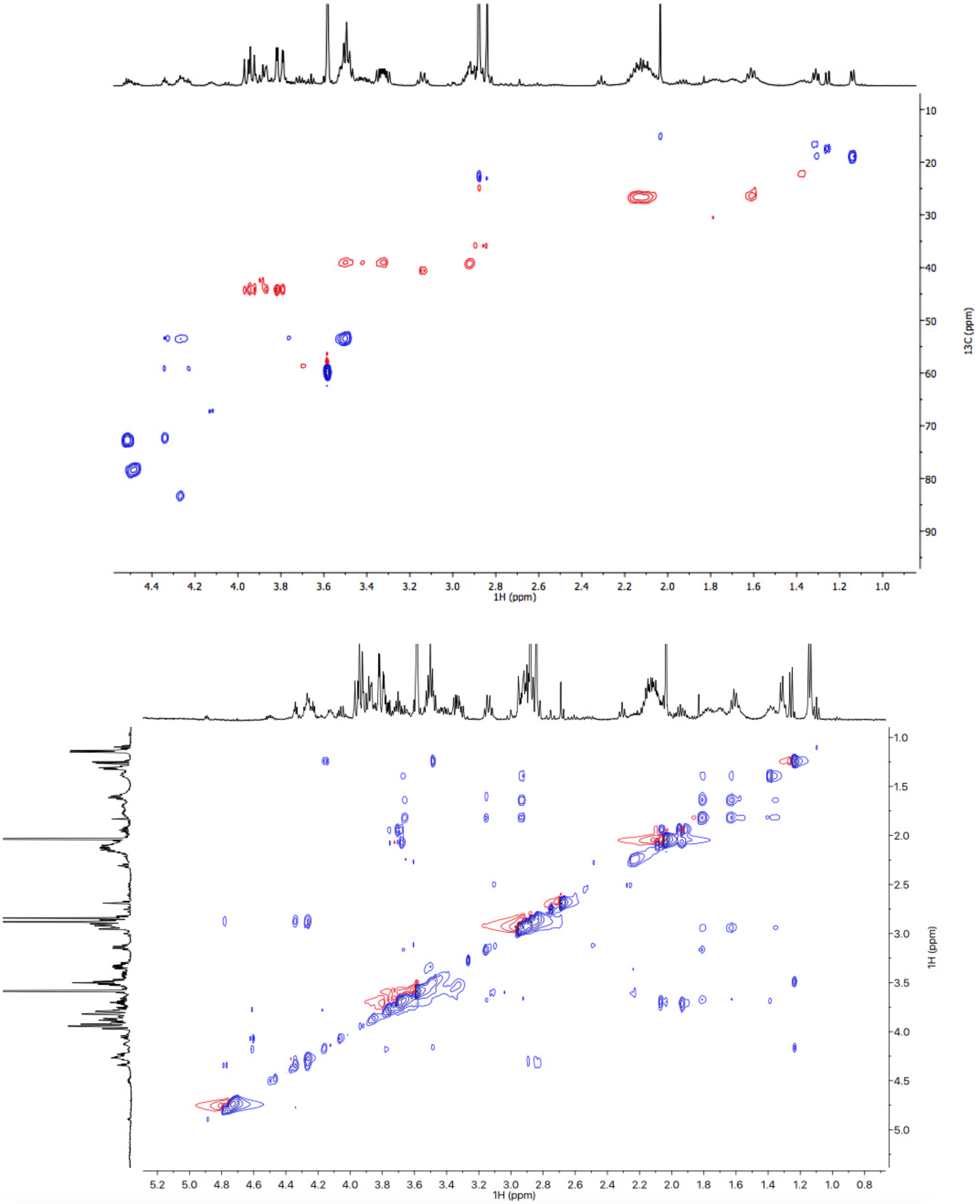

Supplementary Figure S48: Multiplicity-edited HSQC data of the control experiment of methylation of H3K9 peptide in the absence of GLP (top; blue = positive, CH/CH_3, red = negative, CH_2). 2D TOCSY data of controlled experiment of methylation of H3K9 peptide in the absence of GLP (bottom).

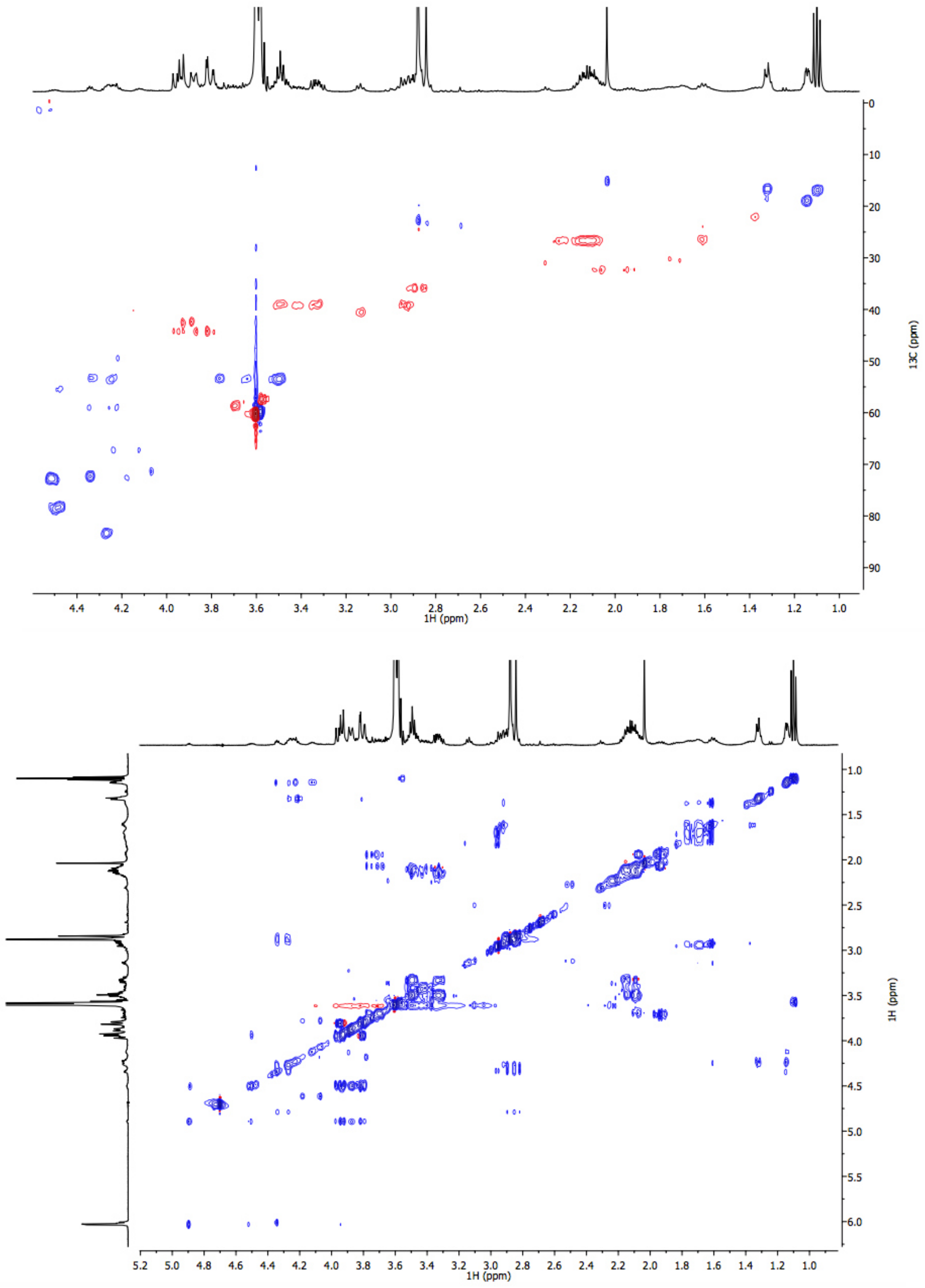

Supplementary Figure S49: Multiplicity-edited HSQC data of GLP-catalyzed methylation of H3Orn9 peptide (top; blue = positive, CH/CH_3, red = negative, CH_2). 2D TOCSY data of GLP-catalyzed methylation of H3Orn9 peptide (bottom).

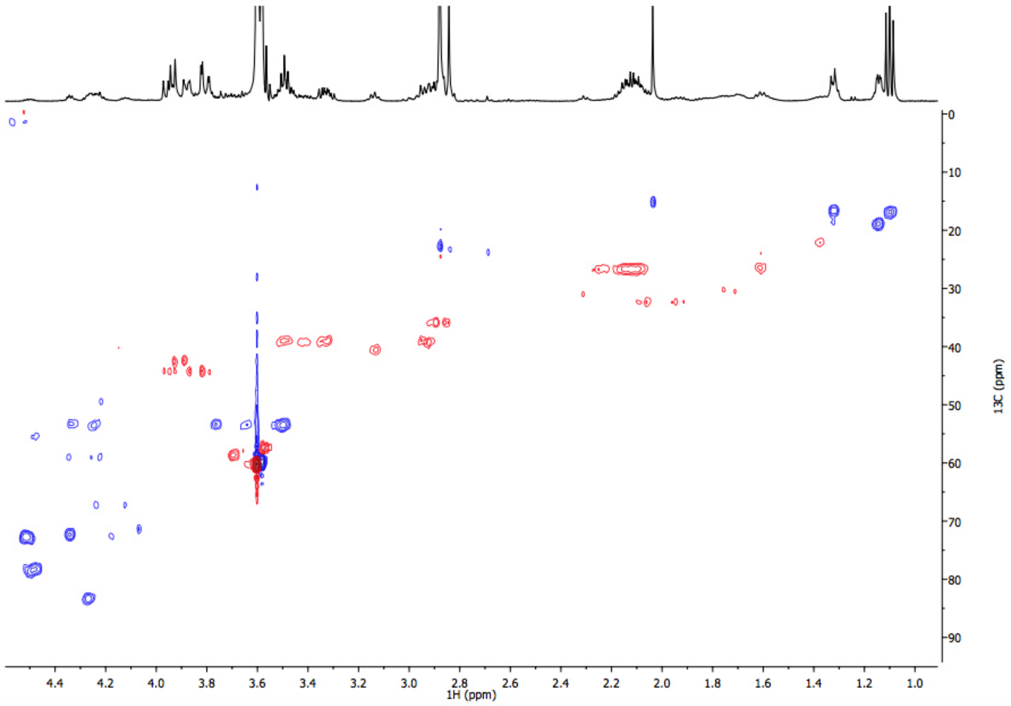

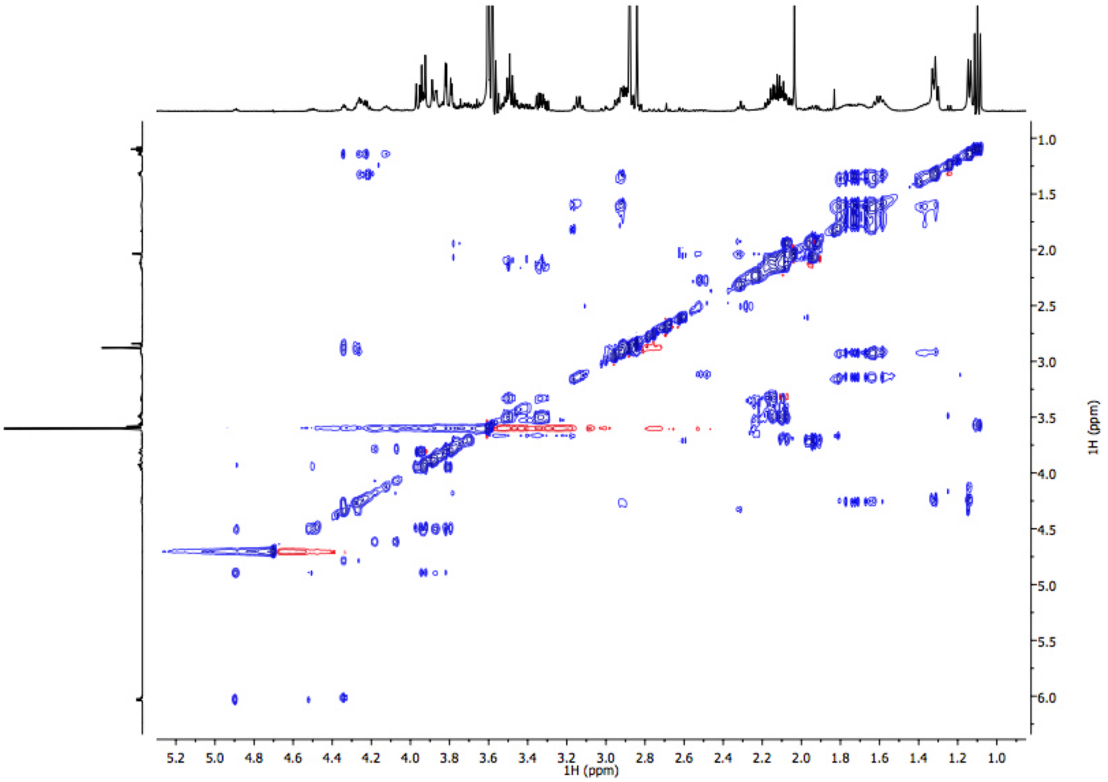

Supplementary Figure S50: Multiplicity-edited HSQC data of GLP-catalyzed methylation of H3hK9 peptide (top; blue = positive, CH/CH_3, red = negative, CH_2). 2D TOCSY data of GLP-catalyzed methylation of H3hK9 peptide (bottom).

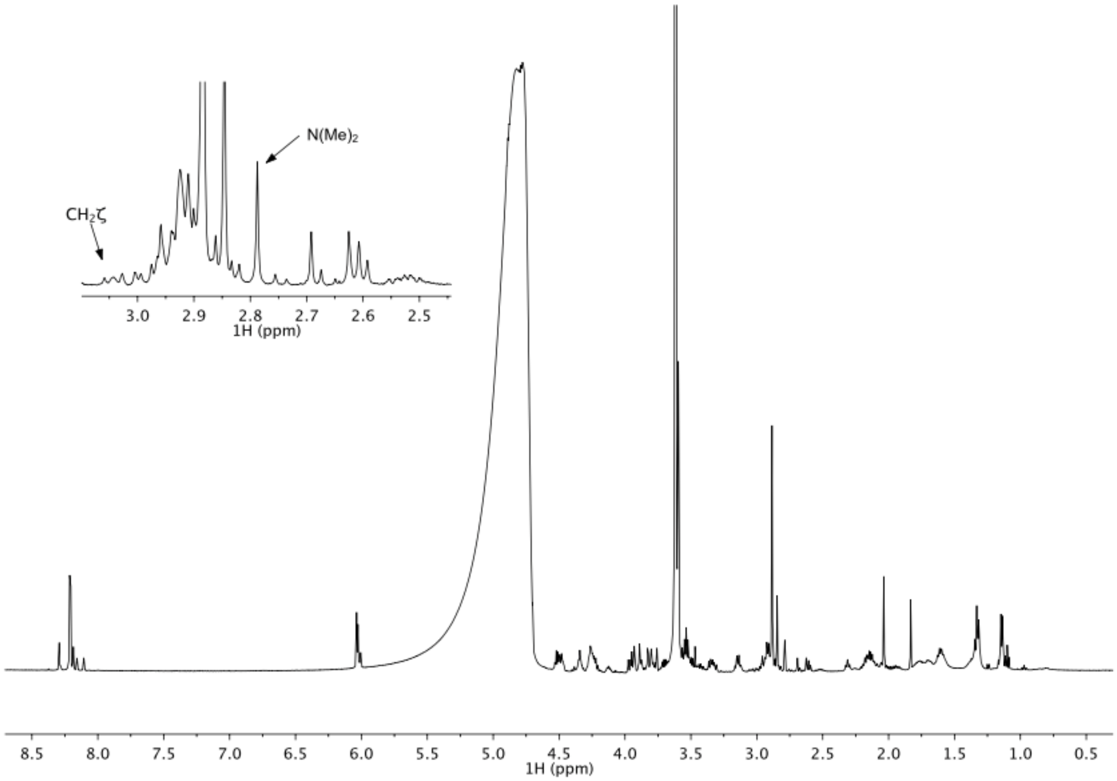

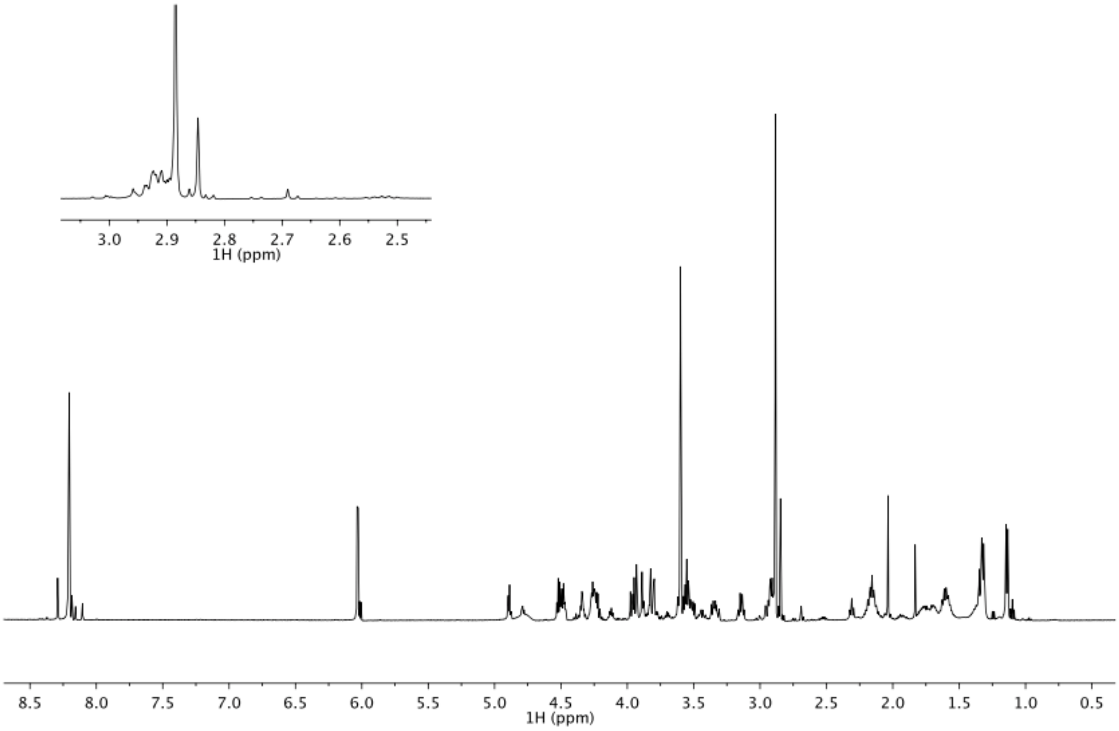

Supplementary Figure S51: ^{1}H NMR spectra of GLP-catalyzed methylation of H3hK9 peptide after 6 h incubation at 37 °C (top; with 15 µM GLP). ^{1}H NMR spectra of the control methylation experiment of H3hK9 peptide in the absence of GLP (bottom).

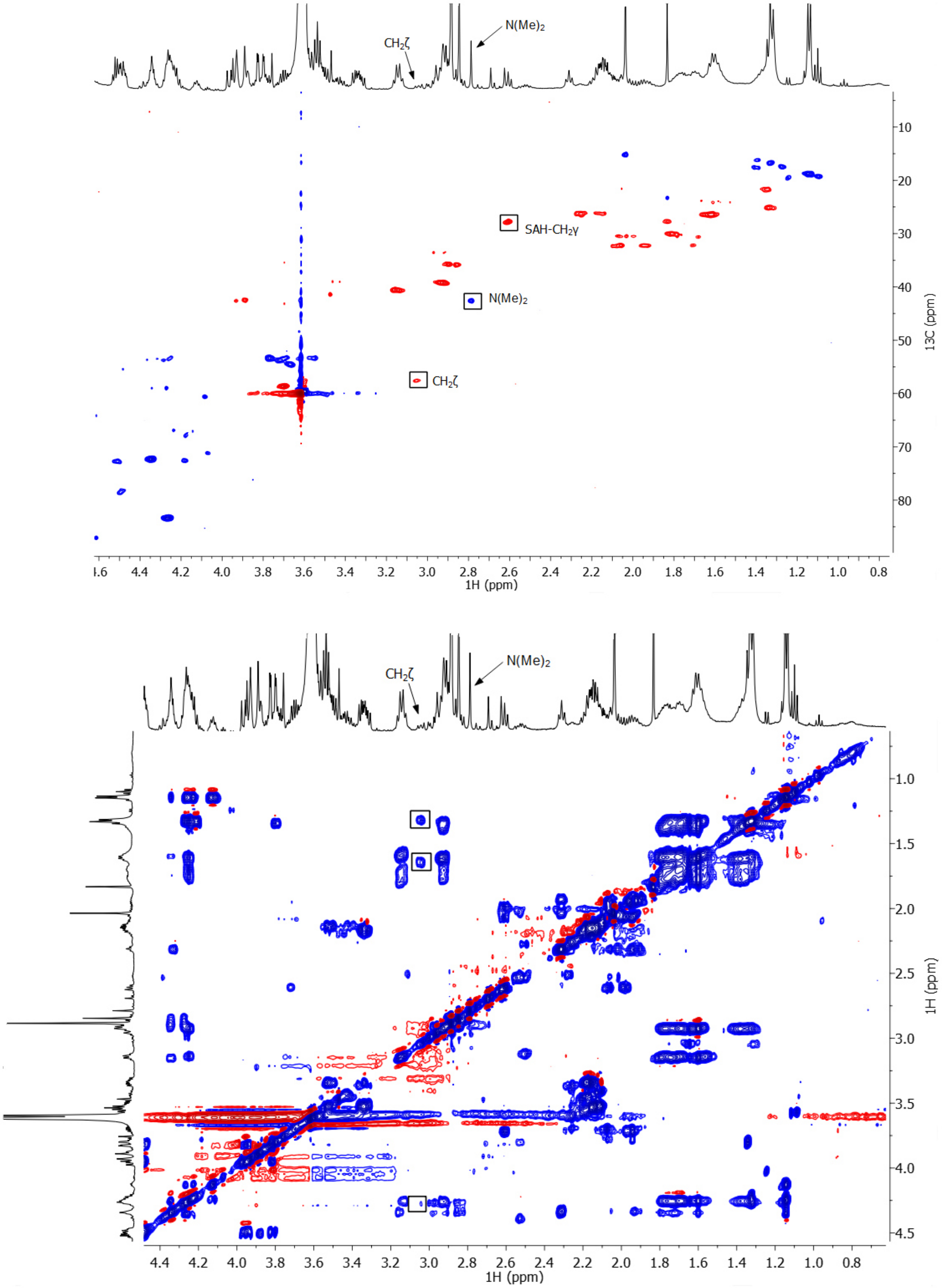

Supplementary Figure S52: Multiplicity-edited HSQC data of GLP-catalyzed methylation of H3hK9 peptide after 6 h incubation at 37 °C (top; blue = positive, CH/CH_3, red = negative, CH_2; with 15 µM GLP). 2D TOCSY data of GLP-catalyzed methylation of H3hK9 peptide after 6 h incubation at 37 °C (bottom; with 15 µM GLP).

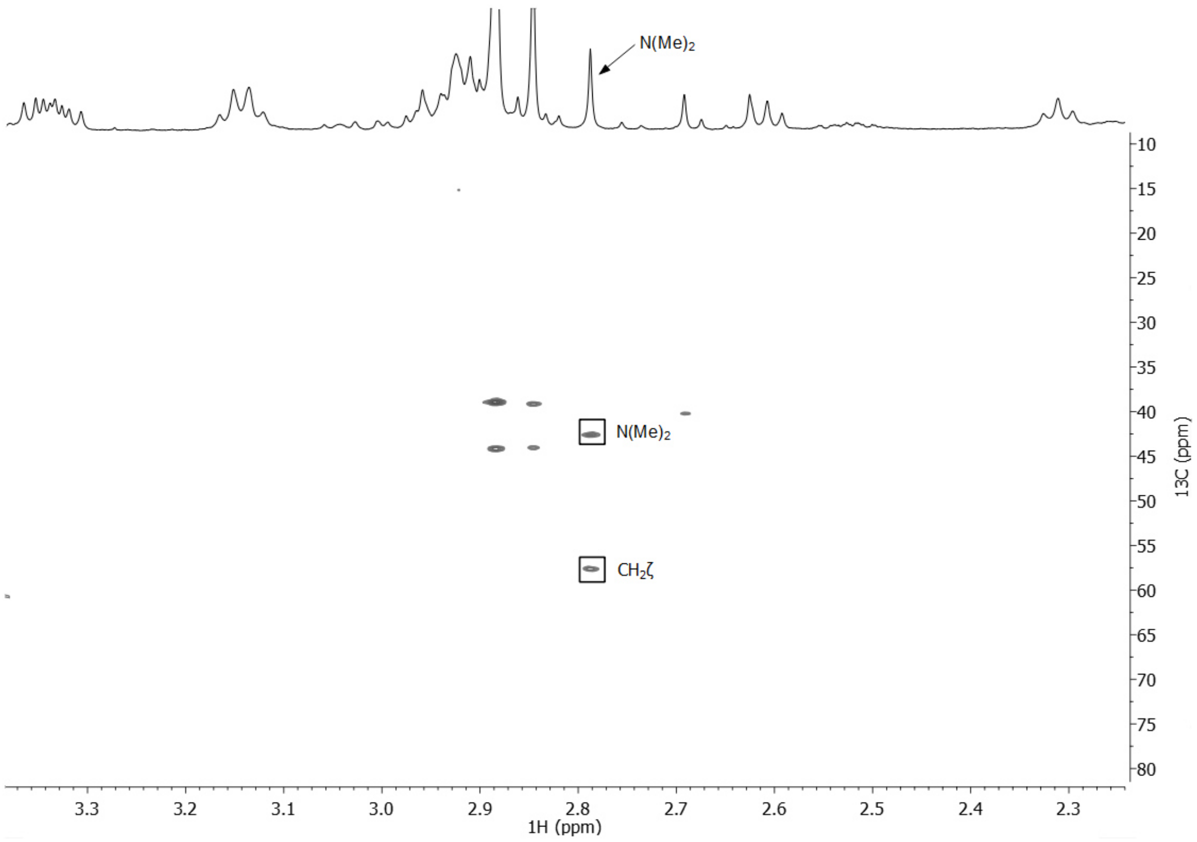

Supplementary Figure S53: HMBC data of GLP-catalyzed methylation of H3hK9 peptide after 6 h incubation at 37 °C in the presence of 15 μM GLP.

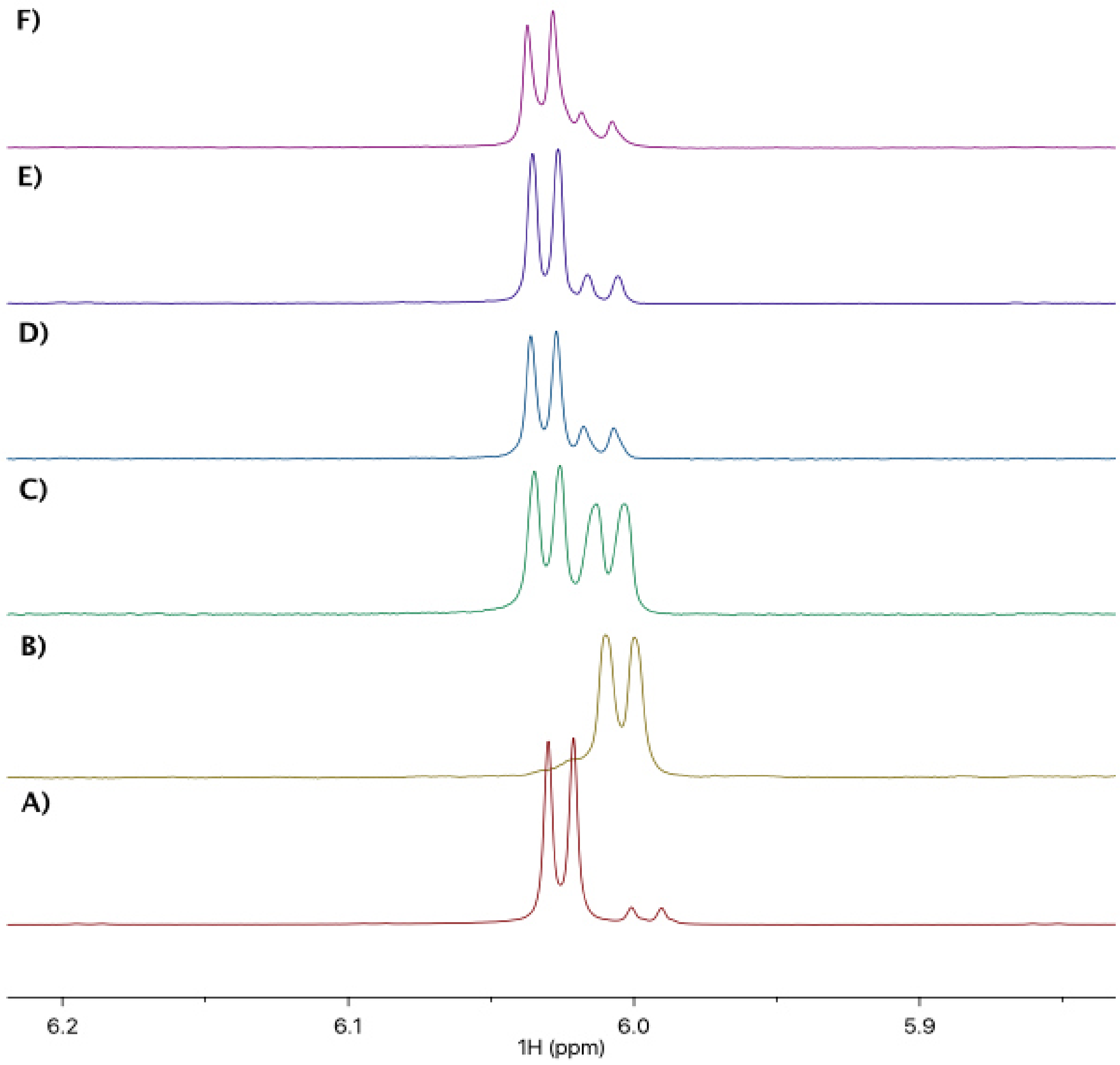

Supplementary Figure S54: Anomeric region of ^{1}H NMR of A) SAM; B) SAH; C) GLP-catalyzed trimethylation of H3K9 peptide; D) controlled experiment of methylation of H3K9 peptide in the absence of GLP; E) GLP-catalyzed methylation of H3Orn9 peptide; F) GLP-catalyzed methylation of H3hK9 peptide.

5. Computational figures

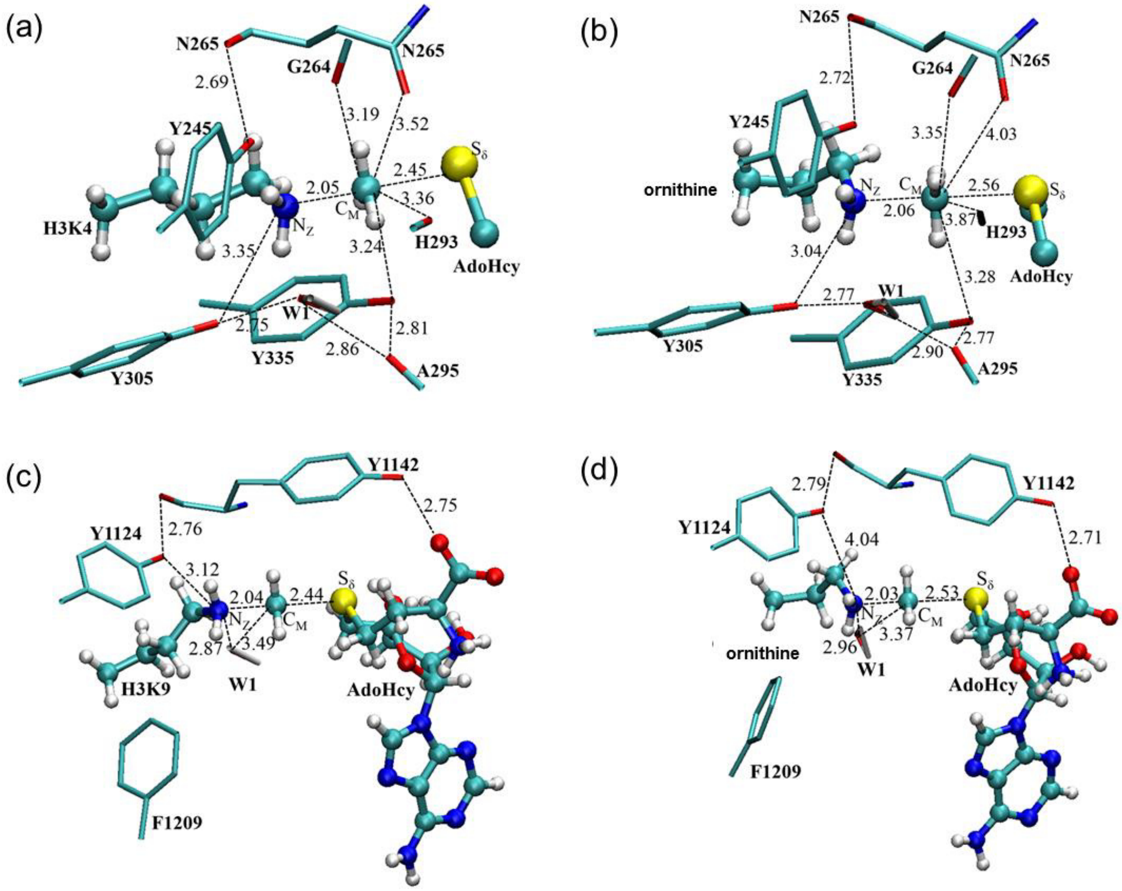

Supplementary Figure S55: (a) Representative active-site structures of near transition state obtained from the free energy simulations for SETD7 with the lysine substrate. (b) Representative active-site structures of near transition state obtained from the free energy simulations for SETD7 with the ornithine substrate. (c) Representative active-site structures of near transition state obtained from the free energy simulations for GLP with the lysine substrate. (d) Representative active-site structures of near transition state obtained from the free energy simulations for GLP with the ornithine substrate.

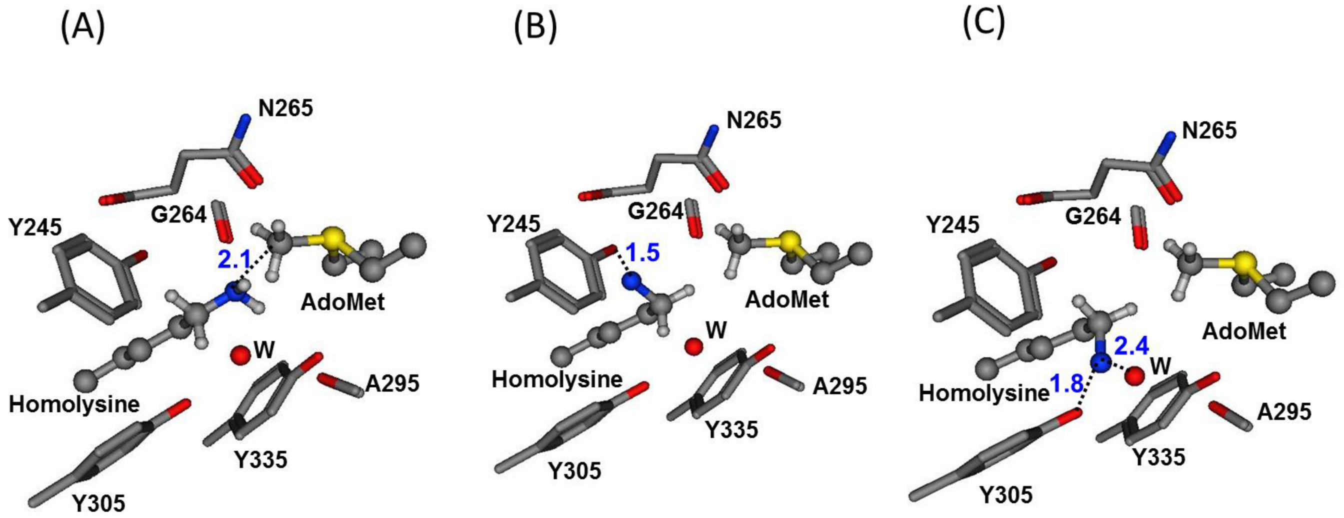

Supplementary Figure S56: Starting with the X-ray structure of SETD7 (1O9S), the possible binding configurations of homolysine at the active site of SETD7 were explored by assuming the Cβ, Cγ, Cδ and Cε atoms were located at the same positions as the corresponding atoms in the SETD7-H3K4me complex. A methyl group was manually added to AdoHcy to change it to AdoMet. The additional carbon atom of homolysine was assumed to occupy the same position as the nitrogen atom of K4me (i.e. changing N to C). The carbon on Me and the two hydrogen atoms were replaced by N along with the change of the corresponding bond distance. This procedure generated three possible conformations of homolysine at the active site. (A) The nitrogen of homolysine at the *tran*s position (i.e., the extended conformation). The nitrogen atom is in bad contacts with the transferable CH_3 group (~2.1 Å) and the transferable methyl points away from its electron lone pair. (B) The nitrogen atom of homolysine at one of the *gauche* positions. The nitrogen atom is in bad contacts with the OH group of Try245 (1.5 Å to O). (C) The nitrogen atom of homolysine at the other *gauche* position. The nitrogen atom is in bad contacts with Tyr305 (1.8 Å) and the water oxygen (2.4 Å).

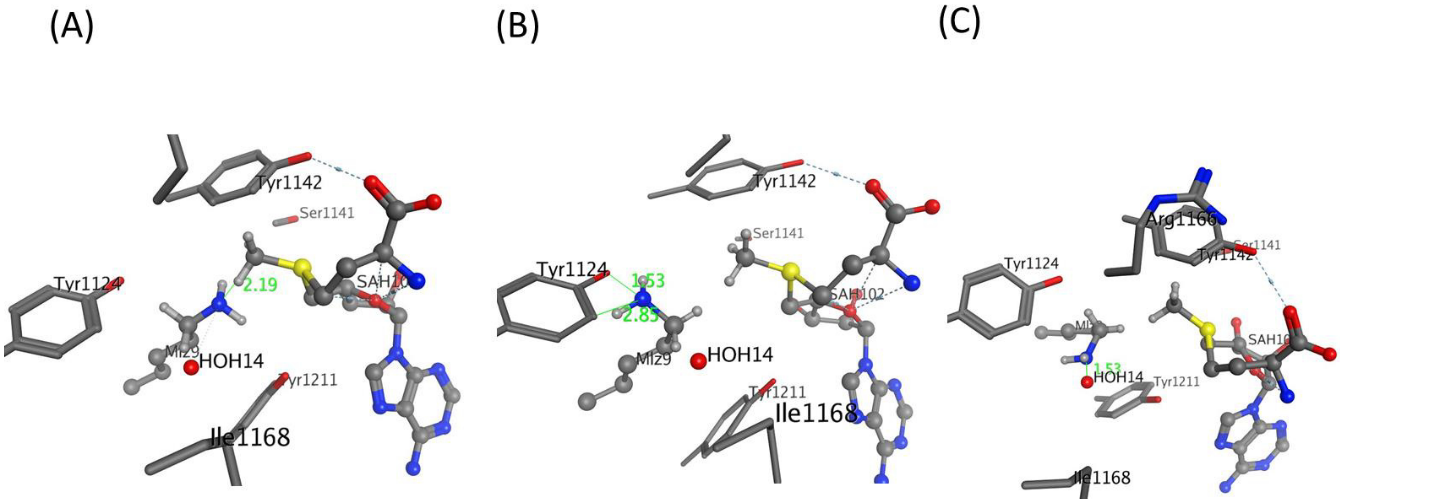

Supplementary Figure S57: Starting with the X-ray structure of GLP (3HNA), the possible binding configurations of homolysine at the active site of GLP were explored by assuming the Cβ, Cγ, Cδ and Cε atoms were located at the same positions as the corresponding atoms in the GLP complex using the same procedure mentioned in Supplementary Figure S56. (A) The nitrogen of homolysine at the *trans* position. The nitrogen atom is in bad contacts with the transferable CH_3 group (~2.2 Å) and the transferable methyl points away from the electron lone pair of N. (B) The nitrogen atom of homolysine at one of the *gauche* positions. The nitrogen atom is in bad contacts with the OH group of Try1124 (1.5 Å to O). (C) The nitrogen atom of homolysine at the other *gauche* position. The nitrogen atom is in bad contacts with the water oxygen (1.5 Å).
